# Evaluation of ketoclomazone and its analogues as inhibitors of 1-deoxy-d-xylulose 5-phosphate synthases and other thiamine diphosphate (ThDP)-dependent enzymes†

**DOI:** 10.1039/d4md00083h

**Published:** 2024-04-02

**Authors:** Alex H. Y. Chan, Terence C. S. Ho, Imam Fathoni, Rawia Hamid, Anna K. H. Hirsch, Kevin J. Saliba, Finian J. Leeper

**Affiliations:** a Yusuf Hamied Department of Chemistry, University of Cambridge Lensfield Road Cambridge CB2 1EW UK fjl1@cam.ac.uk; b Research School of Biology, The Australian National University Canberra ACT 2601 Australia; c Helmholtz Institute for Pharmaceutical Research Saarland (HIPS) – Helmholtz Centre for Infection Research (HZI) Campus Building E8.1 66123 Saarbrücken Germany; d Department of Pharmacy, Saarland University Campus Building E8.1 66123 Saarbrücken Germany

## Abstract

Most pathogenic bacteria, apicomplexan parasites and plants rely on the methylerythritol phosphate (MEP) pathway to obtain precursors of isoprenoids. 1-Deoxy-d-xylulose 5-phosphate synthase (DXPS), a thiamine diphosphate (ThDP)-dependent enzyme, catalyses the first and rate-limiting step of the MEP pathway. Due to its absence in humans, DXPS is considered as an attractive target for the development of anti-infectious agents and herbicides. Ketoclomazone is one of the earliest reported inhibitors of DXPS and antibacterial and herbicidal activities have been documented. This study investigated the activity of ketoclomazone on DXPS from various species, as well as the broader ThDP-dependent enzyme family. To gain further insights into the inhibition, we have prepared analogues of ketoclomazone and evaluated their activity in biochemical and computational studies. Our findings support the potential of ketoclomazone as a selective antibacterial agent.

## Abstract 2

Most pathogenic bacteria, apicomplexan parasites and plants rely on the methylerythritol phosphate (MEP) pathway to obtain precursors of isoprenoids.

## Graphical abstract

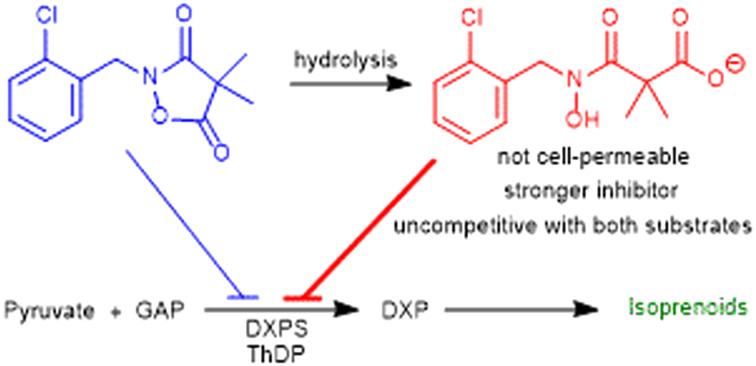

## Introduction

**Fig. 1:**
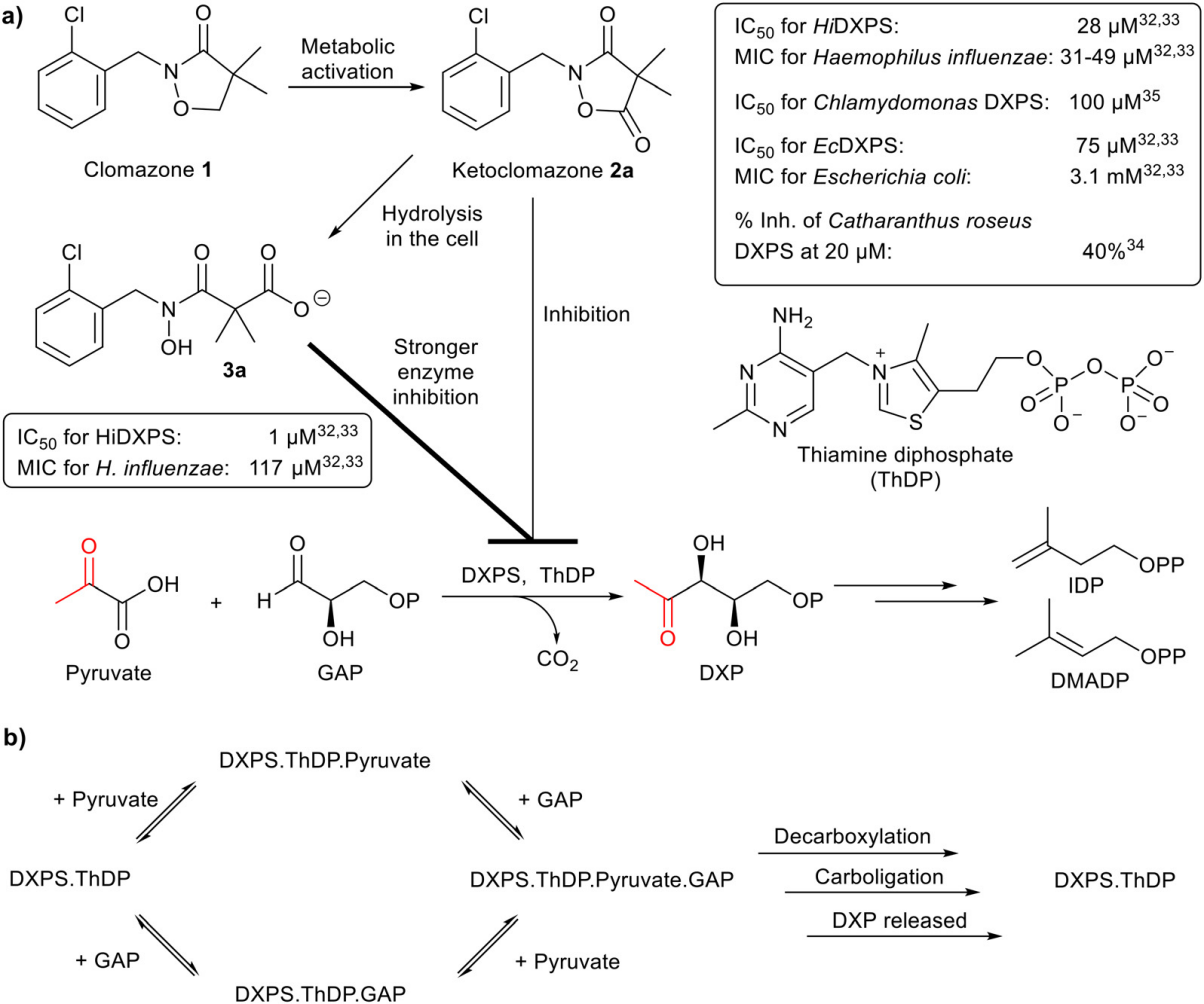
a) Biochemical reaction and inhibition of DXP synthase. DXPS, catalysing the first step of the MEP pathway, is the target of 2a and 3a; their biological activities are summarised in the boxes. MIC, minimum inhibitory concentration. b) Random sequential mechanism of DXPS.

Isoprenoids, comprising a vast family of natural products, are key metabolic components of all organisms.^1^ They are derived from the five-carbon isoprenoid precursors isopentenyl diphosphate (IDP) and dimethylallyl diphosphate (DMADP), which are biosynthesised in nature *via* two pathways, namely the 2-methylerythritol phosphate (MEP) and the mevalonate pathways.^2–5^ The precursors of IDP and DMADP are pyruvate and d-glyceraldehyde 3-phosphate (GAP) in the MEP pathway (Fig. 1a), but solely acetyl coenzyme A in the mevalonate pathway. While mammals exclusively use the mevalonate pathway for isoprenoid biosynthesis, most eubacteria, chloroplast-containing plants and apicomplexan parasites (including *Plasmodium falciparum*) rely on the MEP pathway.^3–6^ In the first and rate-limiting step of the MEP pathway, pyruvate and GAP are converted into 1-deoxy-d-xylulose 5-phosphate (DXP) and CO_2_ by DXP synthase (DXPS), a thiamine diphosphate (ThDP)-dependent enzyme.^5–22^ This makes DXPS an attractive target for the development of anti-infectious agents and herbicides.^5,6^ Thus, there have been many studies of DXPS^7–22^ and its inhibition.^23–33^

Clomazone (1), a soil-applied herbicide, is effective against grass and broadleaf weeds in many crops.^34^ Ketoclomazone (2a), a metabolite of 1, is one of the earliest reported DXPS inhibitors.^32–34^ Clomazone treatment causes bleaching of plant seedlings but to be active, it must be oxidised into 2a. By inhibiting DXPS in the MEP pathway, 2a suppresses isoprenoid biosynthesis in plastids and leads to impaired chloroplast development and pigment loss.^5,6,32–34^

Further studies showed that 2a and its ring-opened form, carboxylate 3a, suppressed the growth of two pathogenic bacteria, *Escherichia coli* and *Haemophilus influenzae*, due to inhibition of DXPS (data summarised in Fig. 1a).^32,33^ In general, 3a is a stronger inhibitor of DXPS but 2a is more potent in cell-based assays.^5,6,32–35^ Presumably this is because 2a is more hydrophobic and diffuses better through the membrane and, once inside the cell, 2a is then hydrolysed to 3a for stronger inhibition of DXPS.^5^ Kinetic studies revealed that the inhibitory action of 2a on *Hi*DXPS and *Ec*DXPS is uncompetitive with respect to pyruvate and mixed type (close to non-competitive) with respect to GAP.^32,33^ The mode of inhibition by 3a has yet to be determined.

In this study, we studied the activities of 2a and 3a against a range of ThDP-dependent enzymes, DXPS enzymes from four species and four other ThDP-dependent enzymes, namely pyruvate dehydrogenase E1-subunit (PDH E1), pyruvate decarboxylase (PDC), pyruvate oxidase (PO) and 2-oxoglutarate dehydrogenase E1-subunit (OGDH E1). Both 2a and 3a inhibited not only *Ec*DXPS (consistent with the earlier findings^32,33^) but also PDH E1, and we determined their modes of inhibition. They showed little or no inhibition of the DXPS enzymes from the other bacteria or of the other ThDP-dependent enzymes. To further understand their inhibition, we prepared analogues 2b–i and 3b–i and evaluated them as inhibitors of *Ec*DXPS and PDH E1 in biochemical and computational studies. These results may provide useful insights to facilitate further development of selective inhibitors of DXPS as anti-infectious agents and herbicides.

## Results and discussion

### Chemical synthesis of compounds 2 and 3 and analogues

**Scheme 1:**
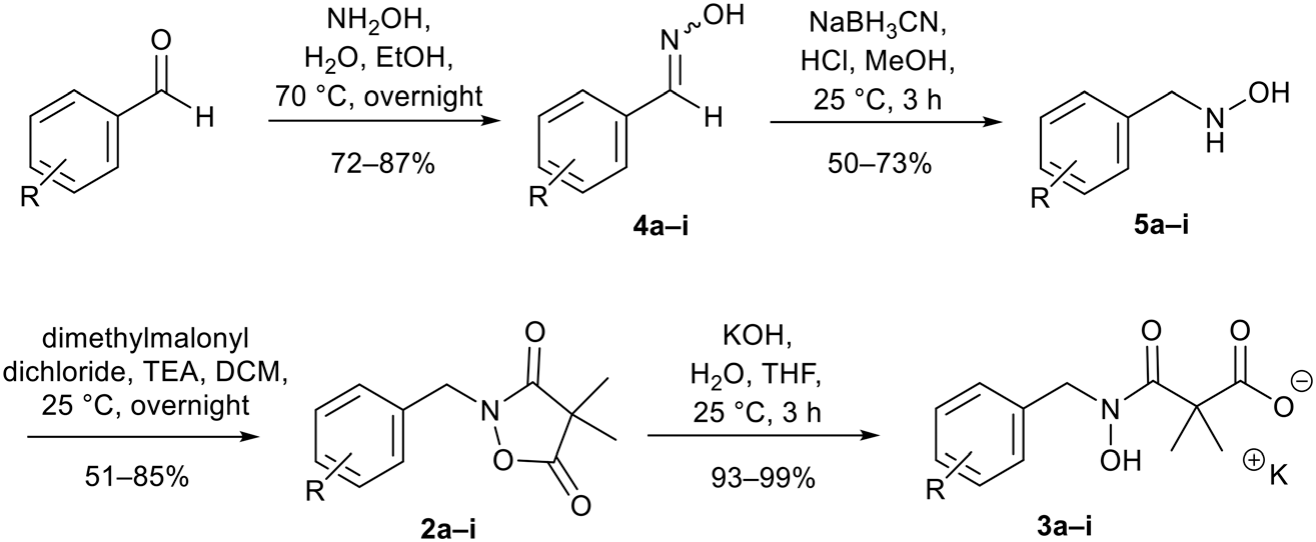
Chemical synthesis of 2a and 3a and analogues. Identity of the R-groups: a: *o*-Cl, b: H, c: *o*-F, d: *o*-Br, e: *p*-Cl, f: *p*-F, g: *p*-Br, h: *o*-Me and i: *p*-^*t*^Bu.

Ketoclomazone 2a and its ring-opened form 3a were synthesised by reported methods.^33^ As shown in Scheme 1, *o*-chlorobenzaldehyde was condensed with NH_2_OH to give oxime 4a, which was reduced with NaBH_3_CN under acidic conditions to give hydroxylamine 5a. Coupling with dimethylmalonyl dichloride yielded ketoclomazone 2a and hydrolysis under basic conditions afforded the ring-opened form 3a.

A decade ago, the Ohkanda group prepared three analogues of 3a as potential antibacterial agents by modifying the acyclic side chain.^33^ However, all three analogues showed greatly reduced inhibition of DXPS and suppression of the growth of *H. influenzae*.^33^ Thus, in this study, we made analogues of 2a and 3a by modifying the aromatic ring. We chose to change the existing *o*-Cl of 2a and 3a to alternative substituents and we also tested some *para*-substituted analogues as this is probably the most exposed position, susceptible to metabolic oxidation. Eight analogues of each (2b–i and 3b–i) were synthesised by the synthetic route in Scheme 1; among these, 2c and 2d were described in a patent in 1981.^36^

### Identifying inhibitory activities of compounds 2 and 3 on *Ec*DXPS and porcine PDH E1

Inhibition by 2a and 3a was evaluated on DXPS enzymes from four different species, namely, *Klebsiella pneumoniae*, *Pseudomonas aeruginosa*, *Deinococcus radiodurans* and *E. coli*. Both were inhibitors of *Ec*DXPS (87–92% inhibition at 1 mM), consistent with earlier reports, but they had low activity on *Kp*DXPS, *Pa*DXPS and *Dr*DXPS (<16% inhibition at 1 mM§§*Kp*DXPS was assayed at 10 mM of inhibitor and showed 64% inhibition by 2a but calculation suggests this would equate to <16% inhibition at 1 mM.) (Table S1†). The selectivity of 2a and 3a as inhibitors of DXPS over other ThDP-dependent enzymes has not yet been reported, so they were also tested against four other ThDP-dependent enzymes: they showed inhibition of porcine PDH E1 (52–71% inhibition at 100 μM) but lacked activity on PDC, PO and OGDH E1 (<5% inhibition at 250 μM).

Although apicomplexan parasites (in addition to most bacteria and plants) rely on the MEP pathway for isoprenoid biosynthesis,^3–6^ only antibacterial and herbicidal activities of ketoclomazone 2a and its ring-opened form 3a have been reported. *Pf*DXPS was not available, so the antiplasmodial activities of 2a and 3a were assessed by determining their effects on *in vitro* proliferation of the 3D7 strain of *P. falciparum*. Infected red blood cells were treated with 2a and 3a, and parasite proliferation was measured by performing SYBR-Safe assay, which correlates fluorescence intensity to parasite DNA.^37–39^ Unfortunately, both compounds showed minimal antiplasmodial activities even at high-micromolar levels (Fig. S1†). Given that fosmidomycin, which targets the second enzyme in the MEP pathway, has potent antimalarial activity,^40^ this negative result led us to suspect that, as with *Kp*DXPS, *Pa*DXPS and *Dr*DXPS, 2a and 3a may not be good inhibitors of *Pf*DXPS, despite assumptions to the contrary.^41^

### Kinetic studies of *Ec*DXPS inhibition

The Kuzuyama group studied the inhibition of *Ec*DXPS by ketoclomazone 2a in 2010.^32^ In their assays, conducted in Tris buffer, DXPS seemed to follow a ping-pong bi–bi mechanism (as is normal for ThDP-dependent enzymes^42^) in which pyruvate reacts with the ThDP-bound DXPS and is decarboxylated and then GAP binds and reacts with the resultant intermediate to yield the DXP product.^32^*K*_M_ values of pyruvate and GAP were 48 and 370 μM, respectively. 2a was found to be uncompetitive with respect to pyruvate (with *K*^pyruvate^_I_ = 75 μM) and mixed-type with respect to GAP (with *K*^GAP^_I_ = 220–460 μM).^32^ More recent studies, using different buffers, demonstrated that both pyruvate and GAP can bind to the free enzyme (DXPS·ThDP in Fig. 1b), arguing against a strictly ordered mechanism, and a random sequential mechanism has been proposed for *Ec*DXPS^8,25^ and several other bacterial and protozoan DXPS enzymes, including *P. falciparum*^12^ and *D. radiodurans*.^16–18^ A buffer-optimisation study conducted by the Freel Meyers group showed that *K*^GAP^_M_ values increased with the Tris concentration and they attributed this to the reactivity of the aldehyde group of GAP towards the amino group of Tris.^8^ Conducting *Ec*DXPS assays in HEPES buffer, they found improved reactivity with *K*^pyruvate^_M_ = 49 μM and *K*^GAP^_M_ = 24 μM.^8^ In our work, using the same buffer, we obtained very similar *K*_M_ values for pyruvate and GAP of 50 and 25 μM, respectively (Fig. S2†). With the more recent findings on the mechanism and the improved assay conditions, we aimed to re-examine the interactions between ketoclomazone (2a) and *Ec*DXPS and extend the kinetic studies to inhibition by 3a and by analogues of both 2a and 3a.

**Fig. 2:**
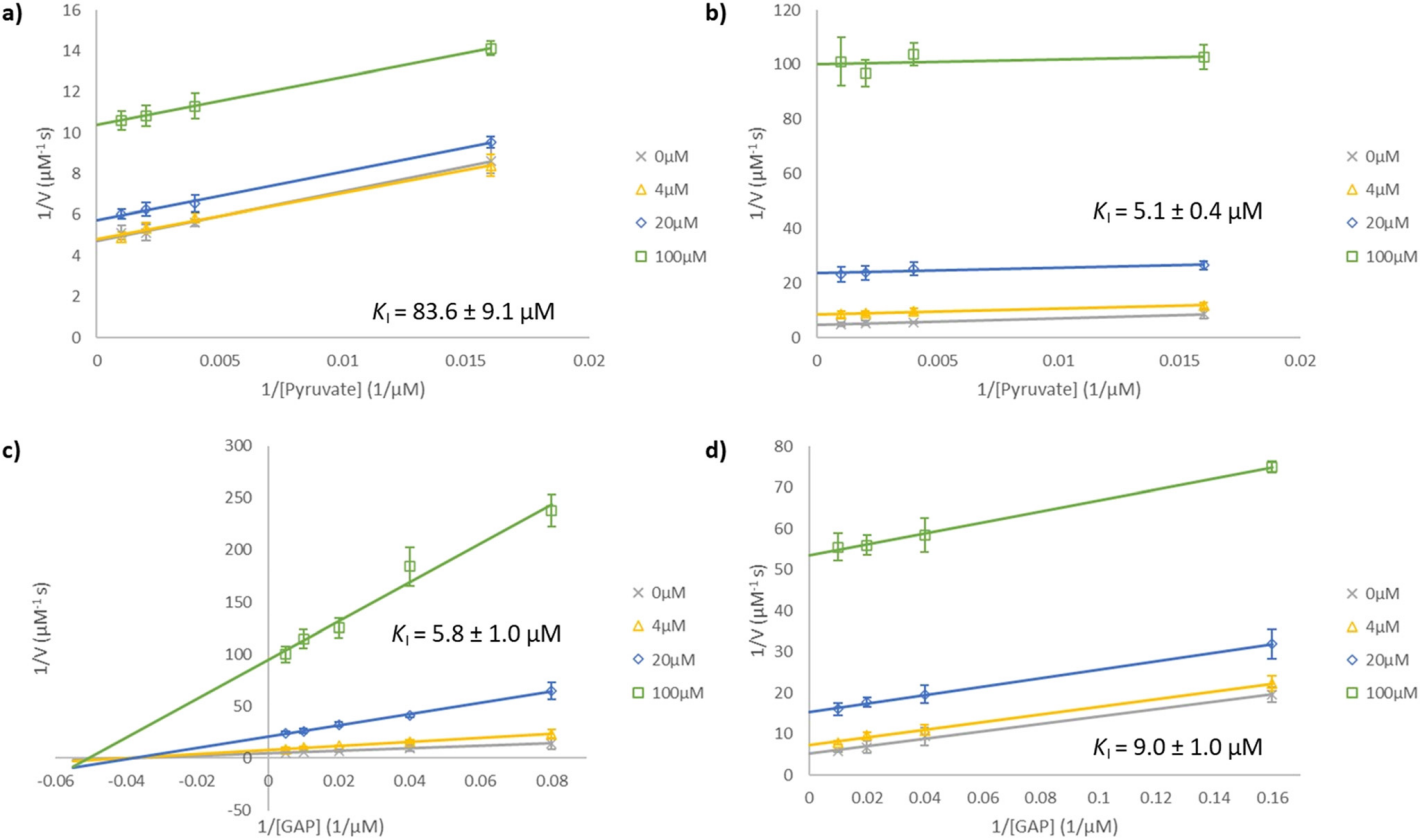
Lineweaver–Burk plots for *Ec*DXPS inhibition. Compounds were assayed at concentrations of 0 μM (×), 4 μM (△), 20 μM (◇) and 100 μM (□). Inhibition by (a) 2a and (b) 3a at a fixed [GAP] of 250 μM (10 *K*_M_) under varying [pyruvate] of 62.5, 250, 500 and 1000 μM. Inhibition by (c) 2a and (d) 3a at a fixed [pyruvate] of 1 mM (20 K_M_) under varying [GAP] of 12.5, 25, 50, 100 and 200 μM and 6.25, 25, 50 and 100 μM respectively. The lines in plot (a), (b) and (d) are essentially parallel, indicating uncompetitive inhibition, while the lines in plot (c) intersect approximately on the *x*-axis (mixed or non-competitive inhibition).

Inhibition of *Ec*DXPS by 2a and 3a in HEPES buffer was evaluated in kinetic studies. As Fig. 2 shows, inhibition by 2a is uncompetitive with respect to pyruvate and mixed (approximately non-competitive) with respect to GAP, with *K*^pyruvate^_I_ measured to be 84 μM at high [GAP] (250 μM = 10 *K*_M_). Our findings on 2a were mostly consistent with those from the Kuzuyama group,^32^ except our *K*^GAP^_I_ at high [pyruvate] (1 mM = 20 *K*_M_) was *ca.* 6 μM compared to their 220–460 μM. This much lower *K*^GAP^_I_ value is presumably due to the different assay conditions used (HEPES buffer and higher [pyruvate]) and is consistent with the much lower *K*^GAP^_M_ in this buffer. The inhibition by 3a was found to be uncompetitive with respect to both pyruvate and GAP, with *K*_I_ values of 5 and 9 μM, respectively (Fig. 2). The shift in inhibition modality with respect to GAP from non-competitive (2a) to uncompetitive (3a), though unexpected, is reasonable given their structural differences.

We then studied how the substitution pattern of the aromatic scaffold affects the inhibition of *Ec*DXPS. The percentage inhibition was measured for compounds at 5 μM under different concentrations of pyruvate and GAP: pyruvate was fixed at either 62.5 μM (= 1.25 *K*_M_, shown as [P]) or 1 mM (= 20 *K*_M_, shown as **[P]**) while GAP was fixed at either 50 μM (= 2 *K*_M_, shown as [G]) or 250 μM (= 10 *K*_M_, shown as **[G]**) (Table 1). The inhibitory activities of analogues 2b–f increased (from 17–27% to 37–48%) with [pyruvate] but did not change with [GAP], consistent with the mode of inhibition by 2a, *i.e.* uncompetitive with respect to pyruvate and non-competitive with respect to GAP. The inhibitory activities of analogues 3b–d increased with both [pyruvate] and [GAP], consistent with the modality of inhibition by 3a, *i.e.* uncompetitive with respect to both pyruvate and GAP. Based on these findings, we speculate that the inhibitory mechanism of ketoclomazone 2a (and 2b–f) is as follows (Fig. 1b): prior to the entry of pyruvate, the inhibitors barely bind to either the DXPS·ThDP free enzyme or the DXPS·ThDP·GAP complex. When pyruvate binds it induces a conformational change in an unidentified inhibitor site (likely to be different from the substrate-binding sites), 2 can then engage the enzyme (with comparable inhibition of both the DXPS·ThDP·pyruvate and the DXPS·ThDP·pyruvate·GAP complexes). In the case of 3a (and 3b–d), it can only engage the enzyme when both substrates are bound as it is uncompetitive towards both, and it exhibits the strongest inhibition when both substrates are bound.

**Table 1:** Summary of inhibitory activity on *Ec*DXPS

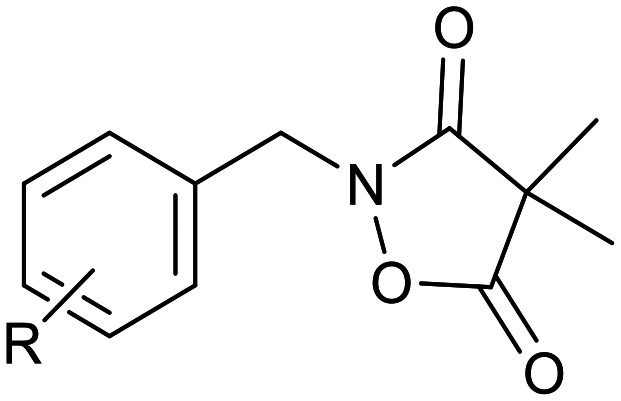					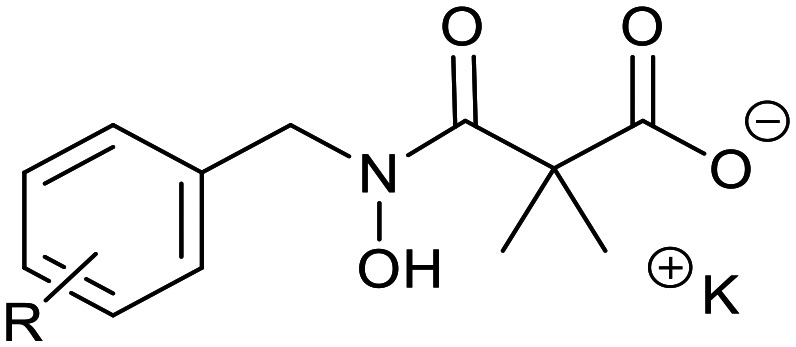				
Compound	Inhibition^a^ (%)				Compound	Inhibition^a^ (%)			
	[P], [G]	**[P]**, [G]	[P], **[G]**	**[P]**, **[G]**		[P], [G]	**[P]**, [G]	[P], **[G]**	**[P]**, **[G]**
2a (*o*-Cl)	24 ± 3	48 ± 2	21 ± 3	47 ± 3	3a (*o*-Cl)	26 ± 2	55 ± 5	48 ± 3	65 ± 5
2b (H)	20 ± 3	37 ± 3	21 ± 2	39 ± 2	3b (H)	26 ± 3	46 ± 5	38 ± 3	53 ± 3
2c (*o*-F)	22 ± 2	48 ± 3	22 ± 3	44 ± 4	3c (*o*-F)	19 ± 2	52 ± 3	30 ± 2	55 ± 2
2d (*o*-Br)	27 ± 2	40 ± 4	27 ± 3	39 ± 4	3d (*o*-Br)	30 ± 2	48 ± 3	37 ± 3	51 ± 4
2e (*p*-Cl)	19 ± 2	42 ± 3	20 ± 4	42 ± 2	3e (*p*-Cl)	14 ± 3	49 ± 2	24 ± 3	23 ± 2
2f (*p*-F)	17 ± 3	37 ± 3	18 ± 2	40 ± 4	3f (*p*-F)	17 ± 3	41 ± 3	28 ± 3	24 ± 2
2g (*p*-Br)	8 ± 2	43 ± 4	19 ± 3	17 ± 4	3g (*p*-Br)	14 ± 2	44 ± 2	24 ± 3	21 ± 4
2h (*o*-Me)	12 ± 2	37 ± 3	21 ± 3	22 ± 2	3h (*o*-Me)	17 ± 3	39 ± 4	23 ± 2	21 ± 2
2i (*p*-^*t*^Bu)	7 ± 2	39 ± 3	15 ± 2	15 ± 3	3i (*p*-^*t*^Bu)	8 ± 3	39 ± 4	18 ± 2	16 ± 3

a Data are the means of measurements in three technical replicates. Percentage inhibition values were determined for compounds at 5 μM with [pyruvate] = 62.5 μM ([P]) or 1 mM (**[P]**) and [GAP] = 50 μM ([G]) or 250 μM (**[G]**).

Analogues 2g–i and 3e–i showed the weakest inhibition (7–17%) under low level of both substrates, improved inhibition (15–28%) under high [GAP], and the strongest inhibition (37–49%) under high [pyruvate] but low [GAP]; such a change in the inhibition profile may have a steric origin as they are all larger than 2a and 3a. As a result, we did not explore any larger substituents in the *ortho*- or *para*-positions. Although none of the *para*-substituted analogues had improved inhibition relative to the unsubstituted 2a, it is worth noting that 2e and 2f (*p*-Cl and *p*-F) have similar activities to 2a. So, if metabolic oxidation at the *para*-position does prove to be a problem, these substituents (in addition to the *o*-Cl) may well provide a solution. While the binding location of 2a and 3a is unknown, with no available crystal structures of them bound to *Ec*DXPS, we believe that their binding sites are likely to be distinct due to their different inhibition modalities. As these inhibitor sites are highly sensitive to structural modifications on ligands and they are probably not in the active site, this may help to explain why 2a and 3a bind poorly to several other DXPS enzymes.

### Kinetic studies on porcine PDH E1 inhibition

**Fig. 3:**
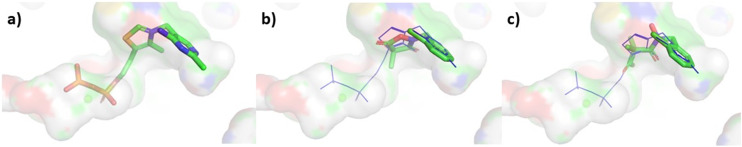
(a) Binding mode of ThDP in PDH E1 showing the V-shaped conformation between the aminopyrimidine and the thiazolium ring. The thiazolium ring is in a relatively hydrophobic region, which facilitates formation of the catalytic ylide. Predicted binding modes of 2a (b) and 3a (c) overlayed with ThDP (blue wires) as in view (a). Molecules were generated using Mercury and dockings were executed using GOLD docking programme with human PDH (PDB: 6CFO) as the target; the reported modelling procedures^42^ have been adopted in this study.

In the preliminary screening on multiple ThDP-dependent enzymes (Table S1†), inhibition of porcine PDH E1 by 2a and 3a was discovered. To gain insights into their binding mode, computational docking was performed: both were predicted to occupy the coenzyme-binding site (Fig. 3). Their competitive relationship with ThDP was confirmed experimentally as the observed potency decreased with increasing [ThDP] (Table 2). With the IC_50_ values of their dose-dependent inhibition determined (Fig. S3†), the affinities of 2a and 3a were found to be comparable to and 2.5 times weaker than that of ThDP, respectively; as the *K*_M_ value for ThDP is 50 nM,^31^ this puts their *K*_I_ values in the nanomolar range (*K*_I_ of 2a = 44 nM and *K*_I_ of 3a = 127 nM).

**Table 2:** Summary of inhibitory activity on porcine PDH E1

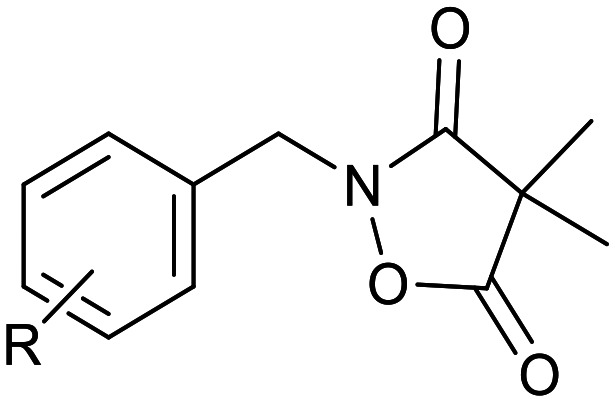				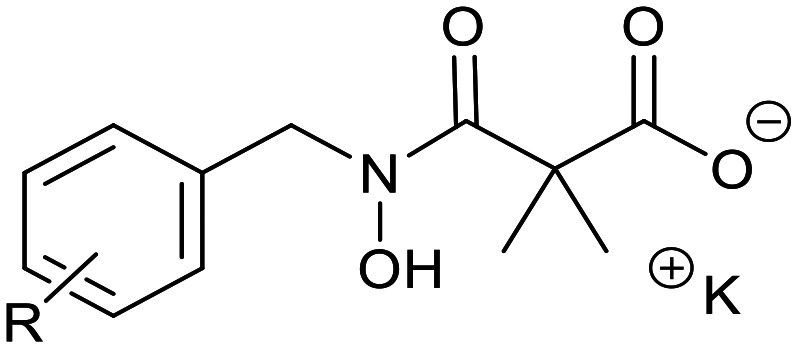			
Compounds	Inhibition^a^ (%)			Compounds	Inhibition^a^ (%)		
	[Compound] : [ThDP]				[Compound] : [ThDP]		
	4 : 1	1 : 1	1 : 4		4 : 1	1 : 1	1 : 4
2a (*o*-Cl)	75 ± 5	53 ± 3	25 ± 4	3a (*o*-Cl)	55 ± 3	32 ± 3	8 ± 3
2b (H)	78 ± 4	54 ± 2	29 ± 2	3b (H)	59 ± 3	31 ± 2	9 ± 2
2c (*o*-F)	74 ± 4	51 ± 3	22 ± 2	3c (*o*-F)	55 ± 4	29 ± 4	<5
2d (*o*-Br)	39 ± 6	18 ± 4	<5	3d (*o*-Br)	17 ± 3	<5	<5
2e (*p*-Cl)	79 ± 4	57 ± 3	19 ± 4	3e (*p*-Cl)	58 ± 2	37 ± 3	<5
2f (*p*-F)	73 ± 4	44 ± 3	13 ± 3	3f (*p*-F)	55 ± 4	23 ± 4	<5
2g (*p*-Br)	37 ± 5	14 ± 4	<5	3g (*p*-Br)	13 ± 4	<5	<5
2h (*o*-Me)	37 ± 4	16 ± 3	<5	3h (*o*-Me)	14 ± 3	<5	<5
2i (*p*-^*t*^Bu)	<5	<5	<5	3i (*p*-^*t*^Bu)	<5	<5	<5

a Data are the means of measurements in three technical replicates. Percentage inhibition values were determined for compounds at 100 μM with [ThDP] = 25 μM (for 4 : 1); at 100 μM with [ThDP] = 100 μM (for 1 : 1); at 25 μM with [ThDP] = 100 μM (for 1 : 4).

The analogues were also tested, to probe the structure–activity relationship (SAR). The ring-opened 3a–h (13–59%) were consistently weaker inhibitors than the cyclic 2a–h (37–79%) at 100 μM with [ThDP] = 25 μM (Table 2). This supports a binding mode in which the non-aromatic ring of our inhibitors occupies the central hydrophobic region (Fig. 3). The charged ring-opened series would suffer a greater desolvation penalty than the cyclic series upon leaving the aqueous environment and binding in this hydrophobic region.^31,43–46^ If the highly polar moiety of the ring-opened series were able to interact with the Mg^2+^ ion in the diphosphate pocket, they would be expected to bind better than the cyclic series, so we suggest that the side chain is not long enough to allow any effective interaction with the Mg^2+^ ion.

Regarding the substitution pattern on the aromatic ring of our inhibitors, the position and identity of the halogen atom (F or Cl) does not seem to affect affinity (compared to the unsubstituted 2b and 3b), but the bromo compounds were weaker binders. For alkyl substituents, having a Me- on the *ortho*-position reduced affinity while introducing a ^*t*^Bu group on the *para*-position abolished binding, presumably for steric reasons. These trends were consistent in both series (Table 2). Our docking models suggested that this *para*-substituent of our inhibitors occupies the same space as the methyl group on the aminopyrimidine ring of ThDP when bound to the enzyme (Fig. 3), and this is supported by the low activity of 2i and 3i: our earlier work^44^ on developing PDH-selective inhibitors showed that substituents larger than a methyl group at this *para*-position led to poor binding. So the predicted binding modes in this study were in line with our understanding of PDH E1's ThDP-binding site.

### Further evaluation – cytotoxicity and membrane-permeability

Regardless of their precise binding modes, 2a and 3a were found to be potent inhibitors of PDH E1. Ligand efficiency (LE), measuring the binding energy to its target (in kcal mol^−1^) per heavy atom of the ligand is a widely applied metric in medicinal chemistry;^47^ drug-discovery efforts often aim to develop clinical candidates with LE > 0.3.^47,48^ As the LEs of 2a and 3a are 0.61 (binding energy = 10.4 kcal mol^−1^; heavy atom count = 17) and 0.54 (binding energy = 9.8 kcal mol^−1^; heavy atom count = 18), respectively, both are highly efficient inhibitors of PDH E1.

Given that the PDH complex provides a link between glycolysis and mitochondrial metabolism for energy production,^49^ concern was raised over possible cytotoxicity due to PDH inhibition. To test this, human foreskin fibroblast (HFF) cells were subjected to compounds 2a and 3a, and the compounds were only weakly cytotoxic, even at concentrations 100 times greater than their *K*_I_ values (Fig. S4†). Although one possible explanation would be that 2a and 3a are weak inhibitors of human PDH E1 despite their potent inhibition on porcine PDH E1, it is generally accepted that the latter is a good model for the former because the sequence of the two enzymes are >95% identical and the residues that differ are located away from the active site.^44^ Another possible explanation would be due to hydrolysis of 2a into 3a.^25^ Extracellular hydrolysis would lead to reduced cell entry of inhibitors as 3a is almost membrane-impermeable (fraction absorbed = 6% in parallel artificial membrane permeability assay, PAMPA,^50^ as shown in Table S2†). Intracellular hydrolysis would lead to reduced entry into the mitochondria, which is where the PDH complex is active.^49^ And in either case inhibition of PDH would be lowered as 3a is a less potent inhibitor than 2a.

Given the inhibitory activity on *Ec*DXPS and the lack of cytotoxicity on HFF cells, the potential of ketoclomazone 2a as an antibacterial agent against susceptible bacteria is enhanced. Previous studies had already established its micromolar activities on suppressing bacterial cell growth,^32,33^ in addition to its herbicidal action. This study provides additional insight into its antibacterial potential. The cyclic form 2a probably serves as a prodrug that facilitates membrane permeability (fraction absorbed = 47% in PAMPA, Table S2†); once inside the cell, it gets hydrolysed to its ring-opened form 3a, which has several important biological consequences: a) this charged form may be trapped within the cells, thus raising the intracellular inhibitor level; b) 3a is a weaker inhibitor of PDH than its parent 2a, thus likely to be less cytotoxic to human cells; and c) although 2a and 3a are both potent inhibitors of DXPS, the shift in inhibition modality with respect to GAP from non-competitive (2a) to uncompetitive (3a) makes the latter a better anti-infectious agent. This is because when an enzyme in a metabolic pathway is inhibited, the upstream metabolic processes continue to make new substrates and this will lead to a build-up of the substrates for that enzyme. As the substrate concentration continues to rise, it is likely to eventually exceed the *K*_M_ value and approach saturating conditions. This will lead to a relief of inhibition by competitive inhibitors but an increase in the affinity of uncompetitive inhibitors; in the latter case, further suppression of the metabolic pathway could eventually be catastrophic to cells.^51^ Our biological data may help put these into context. Equimolar concentrations of 2a and ThDP leads to 53% inhibition of PDH E1 activity; if 2a gets hydrolysed to 3a and the cell increases the ThDP content by four-fold, the percentage inhibition would drop to 8% (Table 2). By contrast, the percentage inhibition of DXPS is 24% for 2a at 5 μM with [pyruvate] and [GAP] close to their respective *K*_M_ values; when hydrolysis occurs and, if the substrates accumulate upon DXPS inhibition to the higher levels used in Table 1, the percentage inhibition would rise to 65%.

## Conclusions

Clomazone 1 is a soil-applied herbicide, and ketoclomazone 2a is believed to be the active species. It has also been showed that 2a can be an antibacterial agent and that 2a and 3a probably suppress bacterial growth through inhibition of DXPS.^32–34^ This study revisited compounds 2a and 3a as inhibitors of a range of DXPS enzymes and also evaluated their activities on the broader ThDP-dependent enzyme family. Among the four tested DXPS enzymes, 2a and 3a only potently inhibited *Ec*DXPS. The kinetics of inhibition of 2a was shown to be uncompetitive with respect to pyruvate and non-competitive with respect to GAP, while that of 3a was found to be uncompetitive with respect to both substrates. Screened on four other ThDP-dependent enzymes, 2a and 3a were identified as ThDP-competitive inhibitors of porcine PDH E1. Despite this, however, 2a and 3a showed little cytotoxicity towards human cells, probably because of hydrolysis of 2a to 3a, which is relatively impermeable towards membranes. Our findings and those of others suggest that the more hydrophobic 2a acts as a prodrug for membrane passage and gets hydrolysed to 3a in cells.^5,6,32,33^ The differences in inhibitory activities and modalities on the DXPS and PDH enzymes between 2a and 3a, established in this study, support the potential of ketoclomazone 2a as a selective antibacterial agent. This study expands our understanding on ketoclomazone as an inhibitor of the ThDP-dependent enzyme family and may aid work on the development of DXPS inhibitors.

## Author contributions

AHYC performed the chemical synthesis. TCSH performed the computational studies. IF performed the cell-based assays. AHYC, TCSH and RH performed the enzyme assays. AHYC and TCSH wrote the first draft. FJL supervised the work from AHYC and TCSH. KJS supervised the work from IF. AKHH supervised the work from RH. All authors edited a draft of the manuscript and approved the final version.

## Conflicts of interest

The authors declare no competing financial interest.

## Supplementary Material

MD-015-D4MD00083H-s001
