# Supplementary material for: Evaluation of ketoclomazone and its analogues as inhibitors of 1-deoxy-d-xylulose 5-phosphate synthases and other thiamine diphosphate (ThDP)-dependent enzymes
Source: RSC Med Chem. 2024 Apr 2;15(5):1773–81. doi: 10.1039/d4md00083h (PMC11110791; doi:10.1039/d4md00083h)
Supplement: MD-015-D4MD00083H-s001 [file MD-015-D4MD00083H-s001.pdf]

Electronic Supplementary Material (ESI) for RSC Medicinal Chemistry.
This journal is © The Royal Society of Chemistry 2024

Evaluation of Ketoclomazone and its Analogues as Inhibitors of 1-Deoxy-D-Xylulose 5-Phosphate Synthases and Other Thiamine Diphosphate (ThDP)-dependent Enzymes

Alex H. Y. Chan,[a‡] Terence C. S. Ho,[a‡] Imam Fathoni,[b] Rawia A. A. Hamid,[c,d] Anna K. H. Hirsch,[c,d] Kevin J. Saliba,[b] and Finian J. Leeper[a*]

[a] Yusuf Hamied Department of Chemistry, University of Cambridge, Lensfield Road, Cambridge CB2 1EW, UK.
[b] Research School of Biology, The Australian National University, Canberra, ACT, 2601, Australia.
[c] Helmholtz Institute for Pharmaceutical Research Saarland (HIPS) – Helmholtz Centre for Infection Research (HZI), Campus Building E8.1, 66123 Saarbrücken, Germany.
[d] Department of Pharmacy, Saarland University, Campus Building E8.1, 66123 Saarbrücken, Germany.
[‡] Equal contribution.
*Corresponding author, e-mail address: fjl1@cam.ac.uk

<u>**Supplementary Information (SI)**</u>

Anti-plasmodial Activity Assays – Methods and Results (Figure S1) **S2**

Enzyme Assays – Methods and Results (Table S1, and Figures S2 and S3) **S3**

Cytotoxicity Assays – Methods and Results (Figure S4) **S6**

Parallel Artificial Membrane Permeability Assay – Methods and Results (Table S2) **S7**

Computational Docking – Methods and Results **S8**

Synthetic Experimental Procedures and NMR spectra **S8**

References **S51**

Anti-plasmodial Activity Assays (Figure S1)

This study used human malaria parasite *P. falciparum* strain 3D7 (chloroquine-sensitive) and the same strain expressing an extra copy of TPK with a GFP-tag (*Pf*TPK-GFP) generated as previously described.[1] The intraerythrocytic stage of the parasites were maintained essentially as previously described.[2] The red blood cells used in these experiments were obtained from anonymous donors and were kindly collected on our behalf by the Canberra Branch of the Australian Red Cross Lifeblood. Compounds were tested at concentrations up to a highest final concentration depending on their solubility. Compound stock solutions were prepared in dimethyl sulfoxide (DMSO) followed by dilution in RPMI 1640 medium in the absence of thiamine. The final concentration of DMSO that the parasites were exposed to never exceeded 0.05%. Two-fold serial dilutions were then performed, with each concentration tested in triplicate. The assay was performed as described[3] with some modifications. Experiments were initiated with parasites in the ring-stage, a parasitemia level of 0.5% and a haematocrit of 2%. Chloroquine (0.5 µM) was used as the positive control (*i.e.* complete inhibition of parasite proliferation), and parasites maintained in the absence of any inhibitor represented 100% parasite proliferation. The final volume in each well was 200 µL. Plates were incubated at 37 °C, under an atmosphere of 96% nitrogen, 3% carbon dioxide and 1% oxygen. Parasite proliferation was measured by performing SYBR-Safe assay.[4]

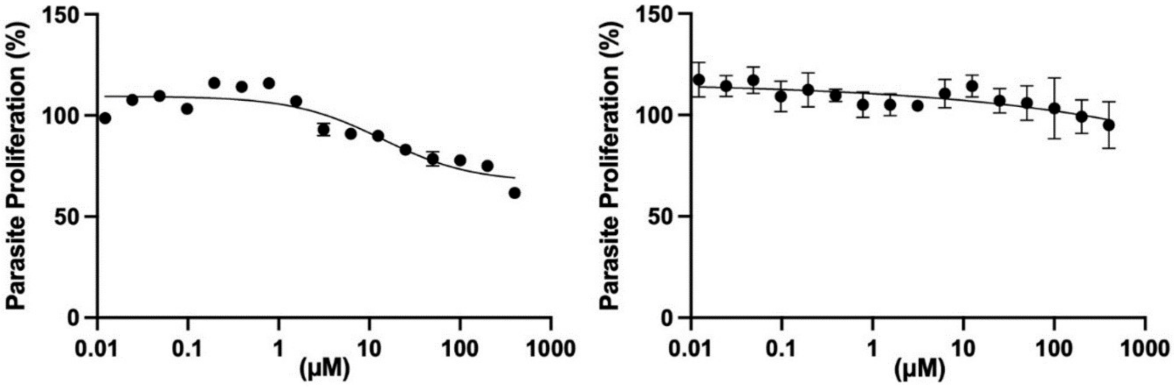

Figure S1. *In vitro* anti-plasmodial activity of compounds **2a** (left) and **3a** (right) against 3D7 parasites in thiamine-free medium (black circles) under assay conditions as described above. Data are averaged from three independent experiments, each carried out in triplicate. Error bars represent SEM and, where not visible, are smaller than the symbols.

Enzyme Assays – Methods and Results (Table S1, and Figures S2 and S3)

Porcine PDH E1 inhibitory activity assay. Porcine PDH E1 was purchased from Sigma. Its activity was determined by monitoring 2,6-dichlorophenolindophenol (DCPIP) reduction at 600 nm using a microplate reader (CLARIOstar) and conducted as described[5,6] with some modifications. The percentage inhibition of compounds against porcine PDH E1 was assayed at final inhibitor concentrations of 25 and 100 µM. The reaction buffer (50 mM KH_2PO_4 and 1 mM $MgCl_2$, pH 7) contained 25 or 100 µM ThDP, 0.25 mM DCPIP, and 2 mg/mL porcine PDH E1, with preliminary screening (Table S1), of compounds at 100 µM and ThDP at 25 µM. The reaction mixture was preincubated at 37 ^{o}C for 30 min, then the reaction was initiated by adding pyruvate to a final concentration of 50 mM. To determine the half-maximal inhibitory concentration (IC_{50}), ThDP concentration was lowered to 10 µM, and inhibitor concentration was varied (1-500 µM). Specific activity was calculated using the molar extinction coefficient of DCPIP, 21 mM^{-1} cm^{-1}.[7] The enzyme IC_{50} values were calculated from non-linear regression curve fitting using GraphPad Prism. K_M(ThDP) was found to be 0.05 µM, consistent with the reported value.[8]

S. cerevisiae **PDC inhibitory activity assay.** *S. cerevisiae* PDC was purchased from Sigma. Its activity was determined by monitoring DCPIP reduction at 600 nm using a microplate reader (CLARIOstar) and conducted as described[5,6] with some modifications. The percentage inhibition of compounds was assayed at a final concentration of 1500 µM. The reaction buffer (50 mM KH_2PO_4 and 1 mM $MgCl_2$, pH 7) contained 300 µM ThDP, 0.27 mM DCPIP, and 0.15 mg/mL *S. cerevisiae* PDC. The reaction mixture was preincubated at 37 ^{o}C for 60 min, then reaction was initiated by adding pyruvate to a final concentration of 70 mM. Specific activity was calculated using the molar extinction coefficient of DCPIP, 21 mM^{-1} cm^{-1}.[7]

E. coli **OGDH E1 inhibitory activity assay.** *E. coli* OGDH E1 was from our previous work[9] and had been donated by R. Frank. Its activity was determined by monitoring DCPIP reduction at 600 nm using a microplate reader (CLARIOstar) and conducted as described[5,6] with some modifications. The percentage inhibition of compounds against *E. coli* OGDH E1 was assayed at a final concentration of 250 µM. The reaction buffer (50 mM KH_2PO_4 and 2 mM $MgCl_2$, pH 7) contained 50 µM ThDP, 0.5 mM DCPIP, and 6.7 mg/mL *E. coli* OGDH E1. The reaction mixture was preincubated at 37 ^{o}C for 60 min, then reaction was initiated by adding α-ketoglutarate to a final concentration of 10 mM. Specific activity was calculated using the molar extinction coefficient of DCPIP, 21 mM^{-1} cm^{-1}.[7]

A. viridans **PO inhibitory activity assay.** *A. viridans* PO and horseradish peroxidase were purchased from Sigma. *A. viridans* PO activity was determined by monitoring appearance of quinoneimine dye at 550 nm using a microplate reader (CLARIOstar) and conducted as described[5] with some modifications. The percentage inhibition of compounds against *A. viridans* PO was assayed at a final concentration of 250 µM. The reaction buffer (50 mM KH_2PO_4 and 10 mM $MgCl_2$, pH 5.9) contained 50 µM ThDP, 10 µM flavin adenine dinucleotide (FAD), 0.15% 4-aminoantipyrine, 0.3% N-ethyl-N-(2-hydroxy-3-sulfopropyl)-m-toluidine (EHSPT), 50 µg/mL horseradish peroxidase and 0.35 U/mL *A. viridans* PO. The reaction mixture was preincubated at 37 ^{o}C for 30 min, then reaction was initiated by adding pyruvate to a final concentration of 50 mM. 1 unit of PO activity is defined as 1 µmol of hydrogen peroxide produced per minute.

DXPS activity assay. DXPS (from *E. coli*, *D. radiodurans*, *P. aeruginosa* and *K. pneumoniae*) and *Ec*IspC were expressed and purified in-house as reported from our previous work.[10-12] DXPS enzyme activity was determined by monitoring NADPH consumption at 340 nm using a microplate reader (CLARIOstar) with some modifications. [10-12] The reaction buffer (100 mM HEPES and 1mM $MgCl_2$, pH 8 for *Ec*DXPS and *Dr*DXPS and pH 7.6 for *Pa*DXPS and *Kp*DXPS) contained 200 µM ThDP, 2.5 mM tris(2-carboxyethyl)phosphine (TCEP), 0.15 mM NAPDH, 0.2 µM DXPS and 1 µM IspC. The reaction mixture was preincubated in the presence or absence of varying inhibitor concentrations at 37 ^{o}C for 10 min, then reaction was initiated by adding varying pyruvate and DL-GAP concentrations. As L-GAP would not affect DXS activity, racemic DL-GAP was used as the substrate of DXPS; the concentration of D-GAP (abbreviated as [GAP] throughout the manuscript and the SI) is calculated as half of the DL-GAP concentration. In the preliminary screening (Table S1), *Ec*DXPS and *Dr*DXPS were tested at [GAP] = 0.2 mM and [Pyruvate] = 1 mM while *Kp*DXPS and *Pa*DXPS were tested at [GAP] = [Pyruvate] = 0.5 mM. Specific activity was calculated using the molar extinction coefficient of NADPH, 6.22 mM^{-1} cm^{-1}.[13]

Table S1. Preliminary screening data.

ThDP-dependent enzymes		Percentage Inhibition	
		Ketoclomazone **2a**	Compound **3a**
DXPS enzymes	*E. coli* DXPS	92% at 1 mM	87% at 1 mM
	D. radiodurans DXPS	13% at 1mM	6% at 1 mM
	K. pneumoniae DXPS	64% at 10 mM	11% at 10 mM
	P. aeruginosa DXPS	11% at 1 mM	10% at 1 mM
Non-DXPS enzymes	Porcine PDH E1	71% at 100 µM	52% at 100 µM
	S. cerevisiae PDC	< 5% at 1.5 mM	< 5% at 1.5 mM
	A. viridans PO	< 5% at 250 µM	< 5% at 250 µM
	E. coli OGDH E1	< 5% at 250 µM	< 5% at 250 µM

Data are the means of measurements in two technical replicates.

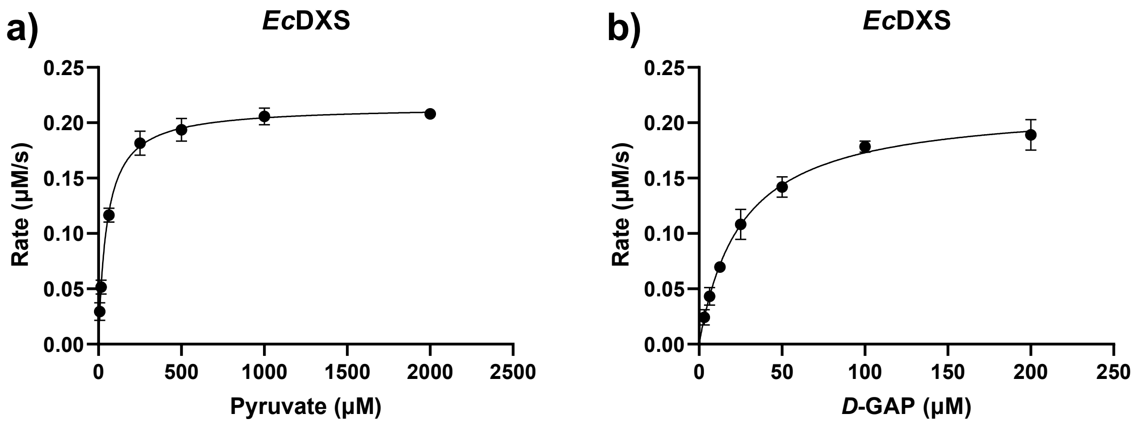

Figure S2. Kinetic analysis of *Ec*DXPS. a) Initial velocities were measured at a fixed [GAP] of 250 µM (10 K_M) under varying [pyruvate] of 8, 16, 63, 250, 500, 1000, 2000 µM. **b)** Initial velocities were measured at a fixed [pyruvate] of 1000 µM (20 K_M) under varying [GAP] of 3, 6, 13, 25, 50, 100, 200 µM. Non-linear regression curve fitting with GraphPad Prism found K_M values of pyruvate and GAP to be 50.5 ± 6.6 µM and 25.4 ± 4.1 µM, respectively. Data are the means of measurements in three technical replicates.

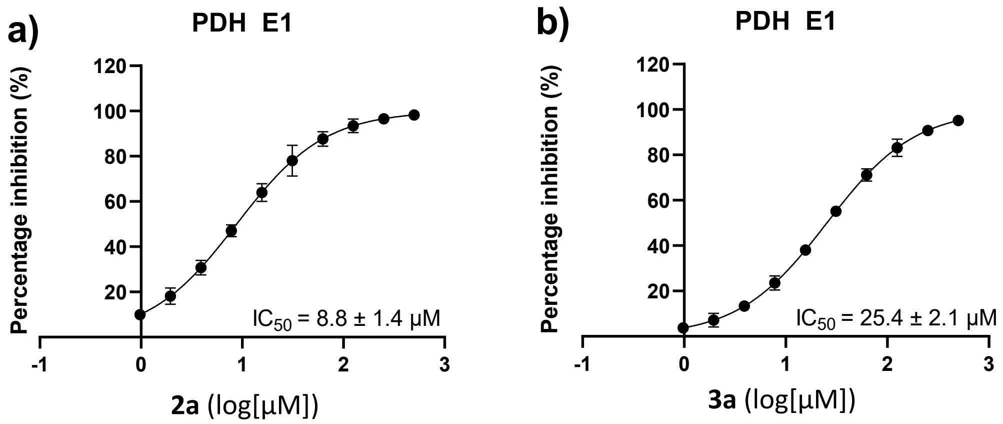

Figure S3. Inhibition of PDH E1 by compounds 2 (a) and 3 (b). IC_{50} values determined at [ThDP] of 10 µM. Data are the means of measurements in three technical replicates. Where error bars are not visible they are smaller than the symbol used.

Cytotoxicity Assays – Methods and Results (Figure S4)

This study used HFF cells (human foreskin fibroblasts cells, kindly supplied by members of the van Dooren Lab, ANU) as described[14] with some modifications. The HFF cells were seeded in 96-well plates at a density of about 13 x 10^4 cells/mL. Cycloheximide (10 µM) was used as a control to indicate complete inhibition of HFF cell proliferation. Plates were incubated at 37 °C in a humidified 5% carbon dioxide incubator for 96 h. A sample of the supernatant (150 µL) was then carefully aspirated from each well and discarded. The plates were then stored at -80 °C. SYBR- Safe assay was used. The plates were thawed, SYBR-Safe lysis solution (150 µL) was added to each well and mixed via pipetting to ensure the HFF cells were detached from the plate and lysed. The plates were then processed as described for the anti-plasmodial assay.

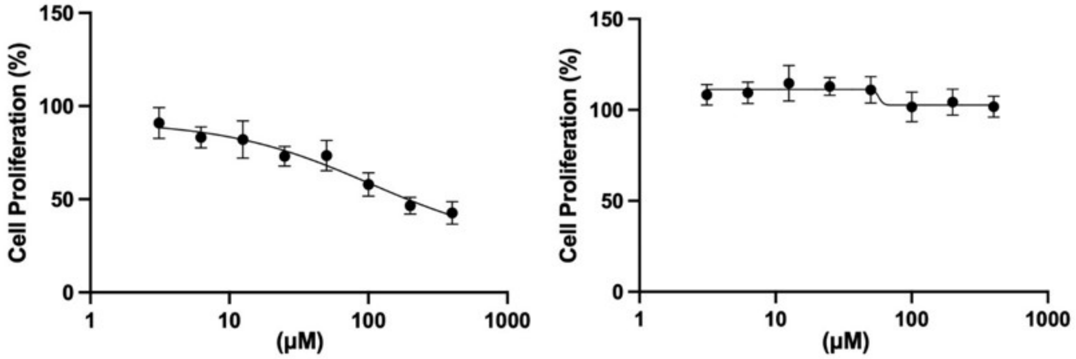

Figure S4. *In vitro* cytotoxicity result of compounds **2a** (left) and **3a** (right) against HFF cells. Data are averaged from three independent experiments, each carried out in triplicate. Error bars represent SEM and, where not visible, are smaller than the symbols.

PAMPA (Parallel Artificial Membrane Permeability Assay) – Methods and Results (Table S2)

PAMPA was carried out in 96-well microtiter filter plates obtained from Millipore as described[15] with some modifications. Each well of the filter plate was impregnated with 15 µL of 5% hexadecane dissolved in hexane (*i.e.* total amount of hexadecane: 0.75 µL) for at least 10 minutes in ventilated environment to allow for complete evaporation of hexane. Donor compartments were filled with 300 µL compound-containing donor solutions of compounds dissolved in 5% DMSO, phosphate buffered saline (PBS) and connected to acceptor plate prefilled with buffer (5% DMSO in PBS, pH 7.4). The resulting sandwich was incubated at room temperature under gentle shaking and wrapped in wet paper towels to avoid evaporation. After 10 hours, the sandwich was disassembled and the solutions in the acceptor and donor were transferred to a disposable UV-transparent plate. UV absorption was measured at wavelengths between 220 and 340 nm using a microplate reader (CLARIOstar). Compounds were tested at 500 µM. Calibration to determine concentration of compounds were performed with varying compound concentrations in buffer (5% DMSO in PBS).

The artificial membrane permeability is expressed as fraction absorbed (Fa%)[16] or log P_e (log of the effective permeability)[15].

$$Fa\% = 100 \cdot C_A \cdot V_A / C_{D0} \cdot V_D$$

where:
C_A = final drug concentration in the acceptor well (µM)
V_A = volume in the acceptor well (cm^3)
C_{D0} = Initial drug concentration in the donor well (µM)
V_D = volume in the donor well (cm^3)

$$\log P_e\ (cm/s) = \log\left[\frac{-\ln[1 - C_A/C_{equilibrium}]}{S(1/V_D + 1/V_A)t}\right]$$

where:
C_A = final drug concentration in the acceptor well (µM)
V_A = volume in the acceptor well (cm^3)
V_D = volume in the donor well (cm^3)
S = surface area (cm^2), typically 0.268 cm^2
t = incubation time(s)
$C_{equilibrium}$ = theoretical equilibrium concentration = $[C_D \cdot V_D + C_A \cdot V_A] / [V_D + V_A]$
where:
C_D = final drug concentration in the donor well (µM)
V_D = volume in the donor well (cm^3)
C_A = final drug concentration in the acceptor well (µM)
V_A = volume in the acceptor well (cm^3)

Table S2. Permeability data.

Compounds	Fa (%)	PAMPA log P_e (cm/s)
**2a**	47.2	-4.8
**3a**	6.2	-5.9

Computational Docking – Methods and Results

Docking of compounds were executed using CCDC GOLD docking program with PDB:6CFO of human PDH E1.[17] The ThDP or equivalent ligand were selected as the binding site. Molecules were generated using Mercury. GA runs were set at 50 and was user defined with population size of 200 and 200000 number of operations. No early termination was permitted. Scaffold constraint to the original ligand was implemented on our compounds to mimic their binding positions. CHEMPLP and GoldScore were the docking scoring and rescoring respectively. For all other GOLD-specific docking options the default settings were used.

Synthetic Experimental Procedures and NMR spectra

General synthesis methods

Oxygen- and moisture-sensitive reactions were carried out in flame-dried glassware under a nitrogen atmosphere. Unless otherwise stated, all chemicals and reagents were purchased from commercial suppliers and used without further purification.

Reaction progress was monitored by analytical thin-layer chromatography (TLC). TLC was conducted using Merck glass plates with silica Kieselgel 60 F254 of thickness 0.25 mm and visualised under 254 nm UV lamp or potassium permanganate staining solution (with light heating).

Flash column chromatography was carried out in the indicated solvent system using prepacked silica gel cartridges for use on the Biotage Purification System. All solvents were removed under reduced pressure using a Büchi rotary evaporator with dry ice traps.

All yields refer to chromatographically and spectroscopically pure compounds unless otherwise stated.

Melting points of compounds were measured using a Reichert machine and are uncorrected.

Compounds were characterised by, at minimum, ^{1}H NMR spectroscopy, ^{13}C NMR spectroscopy and HRMS, unless otherwise stated.

^{1}H NMR spectra were recorded at 400 MHz in $CDCl_3$, CD_3OD, or CD_3SOCD_3 solution on a Bruker 400 MHz spectrometer and chemical shifts were recorded in parts per million (ppm). ^{13}C NMR spectra were recorded at 100 MHz. ^{19}F NMR spectra were recorded at 400 MHz. Resonances are described using the following abbreviations: s (singlet), d (doublet), t (triplet), q (quartet), qnt (quintet), sext (sextet), m (multiplet), br (broad), dd (doublet of doublets), *etc*. Coupling constants (*J*) are given in Hz and are rounded to the nearest 0.1 Hz. All NMR data were collected at 25 °C.

Mass spectra used electrospray ionisation (ESI).

The synthesis and characterisation data for ketoclomazone **2a**[18] and its ring-opened form **3a**[18] have been described.

Experimental procedures – Synthesis

Synthetic scheme:

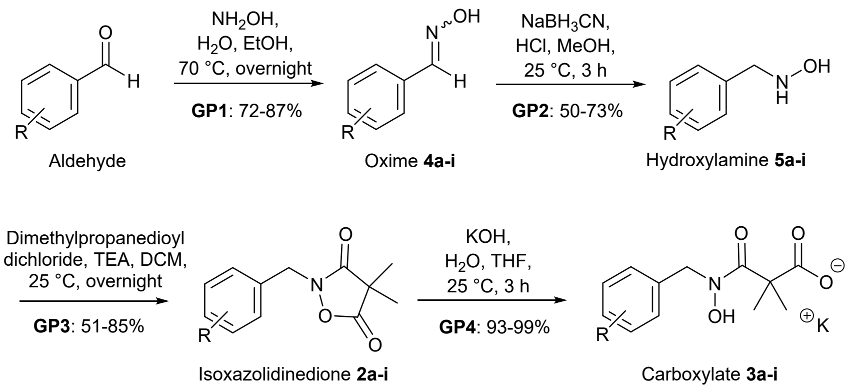

General procedure 1 (GP1) for the preparation of oximes 4a-i:

To a stirred solution of hydroxylamine hydrochloride (6 mmol) and NaOH (6 mmol) in water (3.5 mL, 1.7 M) was added the corresponding aldehyde (0.6 M in EtOH, 8.3 mL, 5 mmol). The reaction mixture was stirred at 70 °C overnight, concentrated under reduced pressure, diluted in EtOAc (100 mL), washed with aqueous phosphate buffer (pH 7) (100 mL), dried over $MgSO_4$, filtered, and evaporated under reduced pressure. The residue was purified by silica flash chromatography (20% EtOAc in hexane) to yield **4a-i**.

General procedure 2 (GP2) for the preparation of hydroxylamines 5a-i:

To a stirred solution of oxime **4a-i** (3 mmol) in MeOH (6 mL, 0.5 M) at 0 °C was added $NaBH_3CN$ (4.5 mmol). The reaction mixture was acidified with 12 M HCl (0.75 mL, 9 mmol), stirred at 25 °C for 3 h, concentrated under reduced pressure, diluted in water (20 mL), basified with K_2CO_3 to pH 10, and extracted with DCM (100 mL). The organic phase was dried over $MgSO_4$, filtered, and evaporated under reduced pressure. The residue was purified by silica flash chromatography (30-80% EtOAc in hexane) to yield **5a-i**.

General procedure 3 (GP3) for the preparation of isoxazolidinediones 2a-i:

To a stirred solution of hydroxylamine **5a-i** (1 mmol) in DCM (5 mL, 0.2 M) at 0 °C was added dimethyl-propanedioyl dichloride (0.14 mL, 1 mmol) and TEA (0.28 mL, 2 mmol) dropwise. The reaction mixture was stirred at 25 °C overnight, diluted in DCM (50 mL), washed with aqueous phosphate buffer (pH 7) (30 mL), dried over $MgSO_4$, filtered, and evaporated under reduced pressure. The residue was purified by silica flash chromatography (20% EtOAc in hexane) to yield **2a-i**.

General procedure 4 (GP4) for the preparation of carboxylates 3a-i:

To a stirred solution of isoxazolidinedione **2a-i** (0.5 mmol) in THF (5 mL, 0.1 M) at 0 °C was added KOH (1 M in water, 0.5 mL, 0.5 mmol) dropwise. The reaction mixture was stirred at 25 °C for 3 h and then concentrated under reduced pressure to yield **3a-i**.

N-(Phenylmethylidene)hydroxylamine **4b**

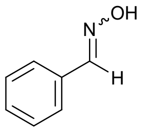

Prepared from benzaldehyde using **GP1**. Thick colourless oil (527 mg, 87%).

^{1}H NMR (400 MHz, CD_3SOCD_3) δ 11.26 (s, 1H), 8.13 (s, 1H), 7.57-7.60 (m, 2H), 7.36-7.42 (m, 3H).

^{13}C NMR (100 MHz, CD_3SOCD_3) δ 148.6, 133.4, 129.7, 129.1, 126.8.

HRMS (ESI) *m/z*: [M+H$^+$] calculated for C_7H_7NO: 122.0600; found: 122.0612.

Analytical data are consistent with those previously reported.[19]

^{1}H NMR of **4b** in CD_3SOCD_3:

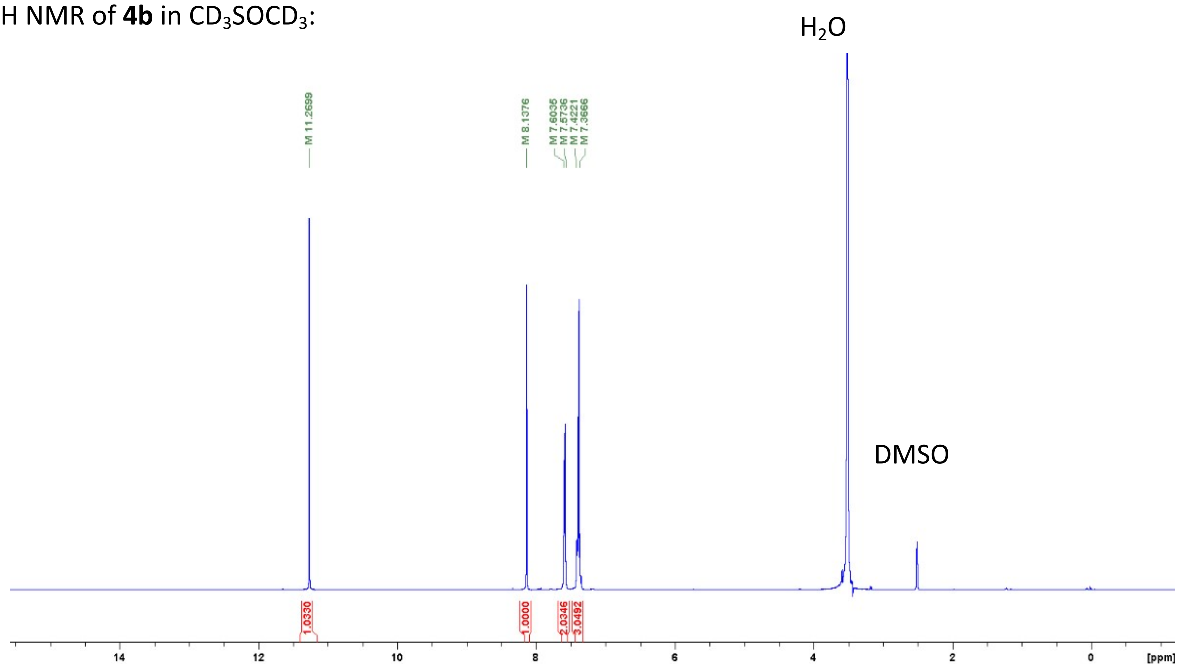

^{13}C NMR of **4b** in CD_3SOCD_3:

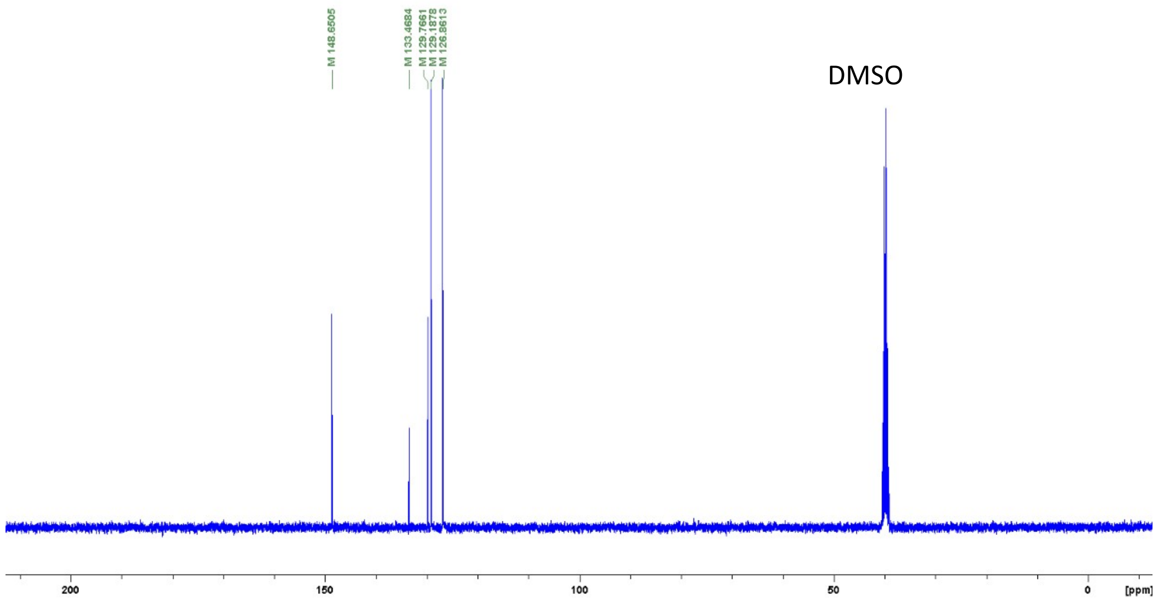

M 148.6505
M 133.4684
M 129.7661
M 129.1878
M 126.8613
DMSO
200
150
100
50
0
[ppm]

N-[(2-Fluorophenyl)methylidene]hydroxylamine **4c**

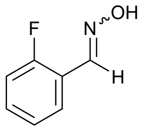

Prepared from 2-fluorobenzaldehyde using **GP1**. Thick colourless oil (576 mg, 83%).

^{1}H NMR (400 MHz, $CDCl_3$) δ 8.84 (br, 1H, OH), 8.41 (s, 1H), 7.74 (m, 1H), 7.39 (m, 1H), 7.19 (m, 1H), 7.12 (m, 1H).

^{13}C NMR (100 MHz, CD_3SOCD_3) δ 160.7 (d, *J* = 253.3 Hz), 144.4 (d, *J* = 3.5 Hz), 131.5 (d, *J* = 8.7 Hz), 127.2 (d, *J* = 2.7 Hz), 124.4 (d, *J* = 3.5 Hz), 119.8 (d, *J* = 10.7 Hz), 116.0 (d, *J* = 21.2 Hz).

^{19}F NMR (400 MHz, $CDCl_3$) δ -118.1.

HRMS (ESI) *m/z*: [M+H$^+$] calculated for C_7H_6FNO: 140.0506; found: 140.0518.

Analytical data are consistent with those previously reported.[19]

^{1}H NMR of **4c** in $CDCl_3$:

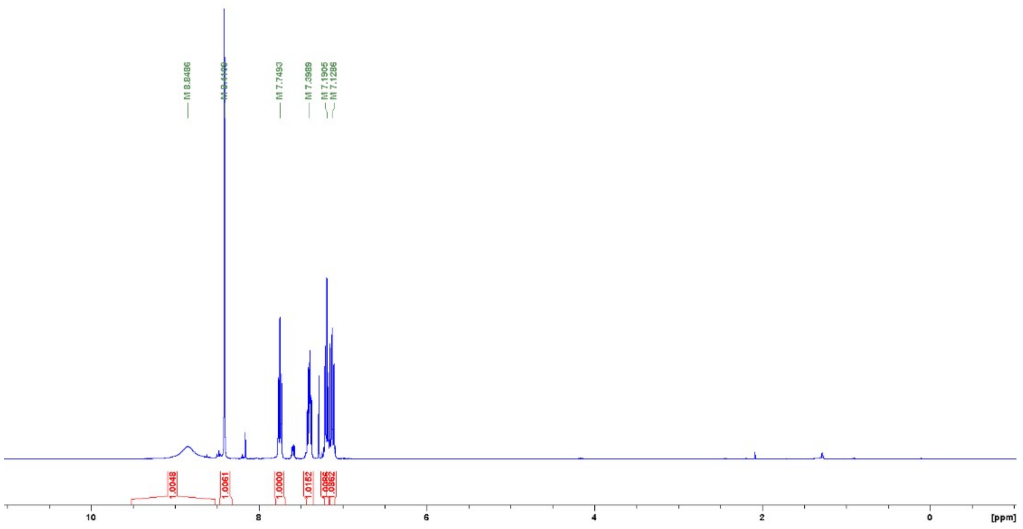

^{13}C NMR of **4c** in $CDCl_3$:

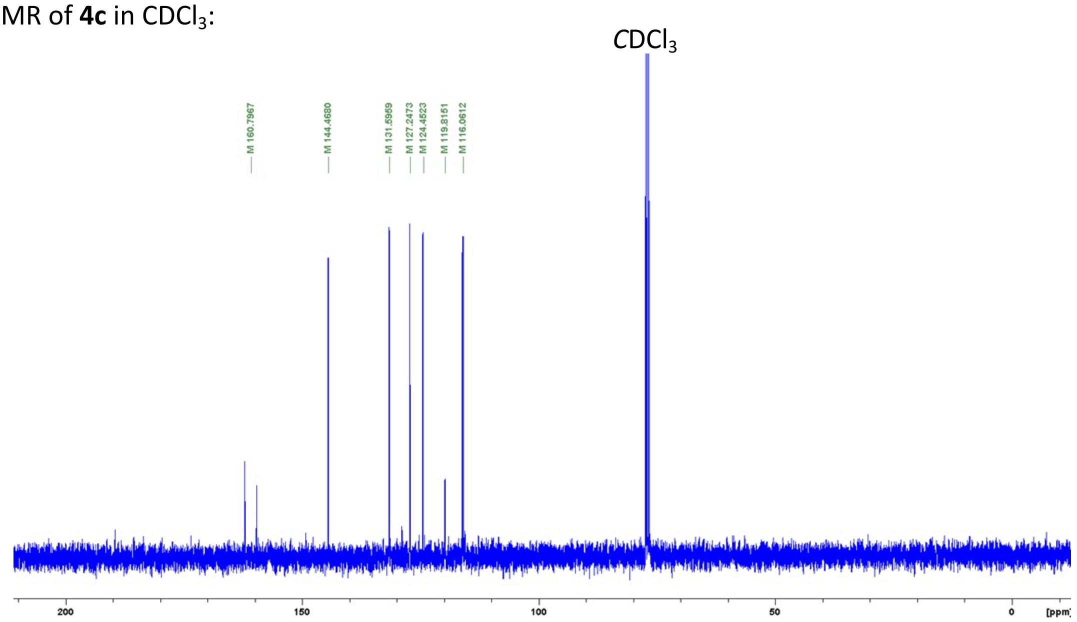

^{13}C DEPT-135 NMR of **4c** in $CDCl_3$:

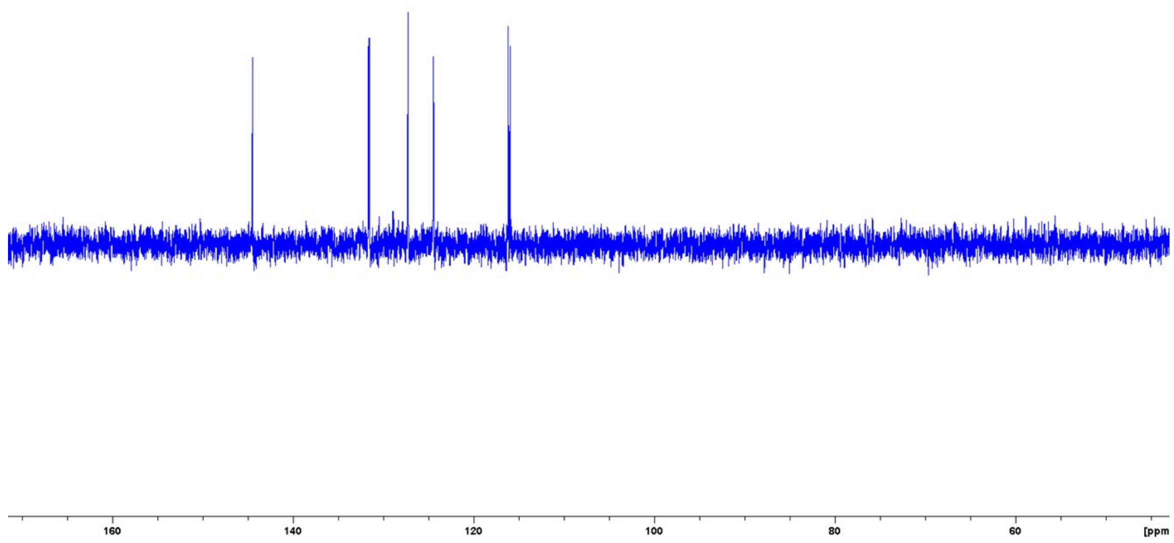

^{19}F NMR of **4c** in $CDCl_3$:

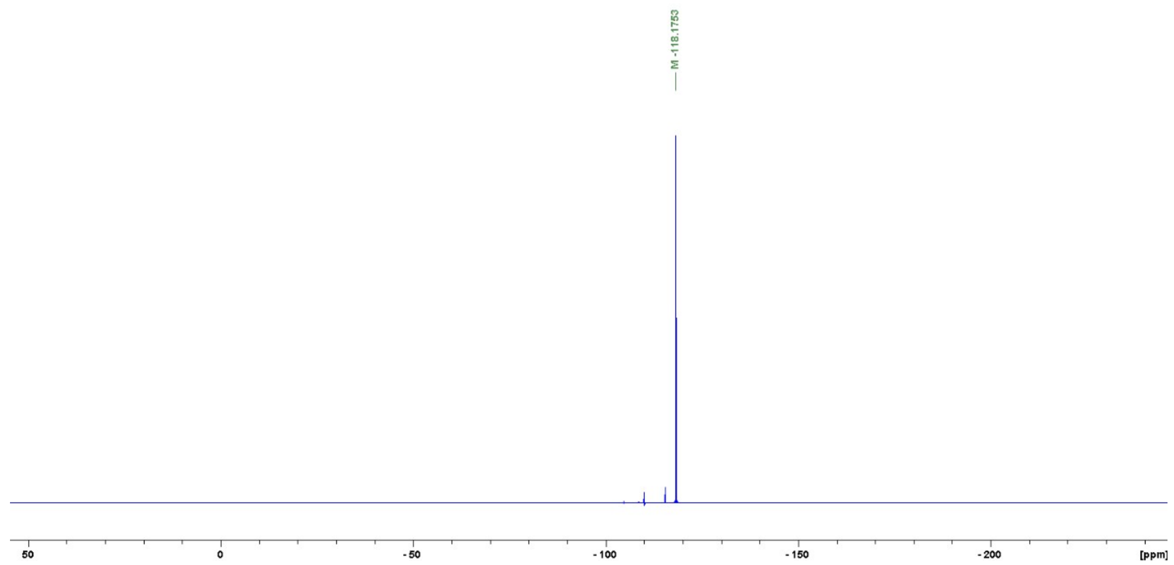

N-[(2-Bromophenyl)methylidene]hydroxylamine **4d**

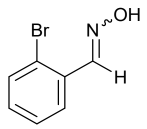

Prepared from 2-bromobenzaldehyde using **GP1**. Thick colourless oil (721 mg, 72%).

^{1}H NMR (400 MHz, CD_3SOCD_3) δ 11.69 (s, 1H), 8.32 (s, 1H), 7.80 (dd, *J* = 1.7, 7.8 Hz, 1H), 7.66 (dd, *J* = 1.1, 7.8 Hz, 1H), 7.41 (dd, *J* = 1.1, 7.8 Hz, 1H), 7.33 (dd, *J* = 1.7, 7.8 Hz, 1H).

^{13}C NMR (100 MHz, CD_3SOCD_3) δ 147.3, 133.4, 132.2, 131.6, 128.5, 127.5, 123.0.

HRMS (ESI) *m/z*: [M+H$^+$] calculated for C_7H_6BrNO: 199.9705; found: 199.9715.

Analytical data are consistent with those previously reported.[19]

^{1}H NMR of **4d** in CD_3SOCD_3:

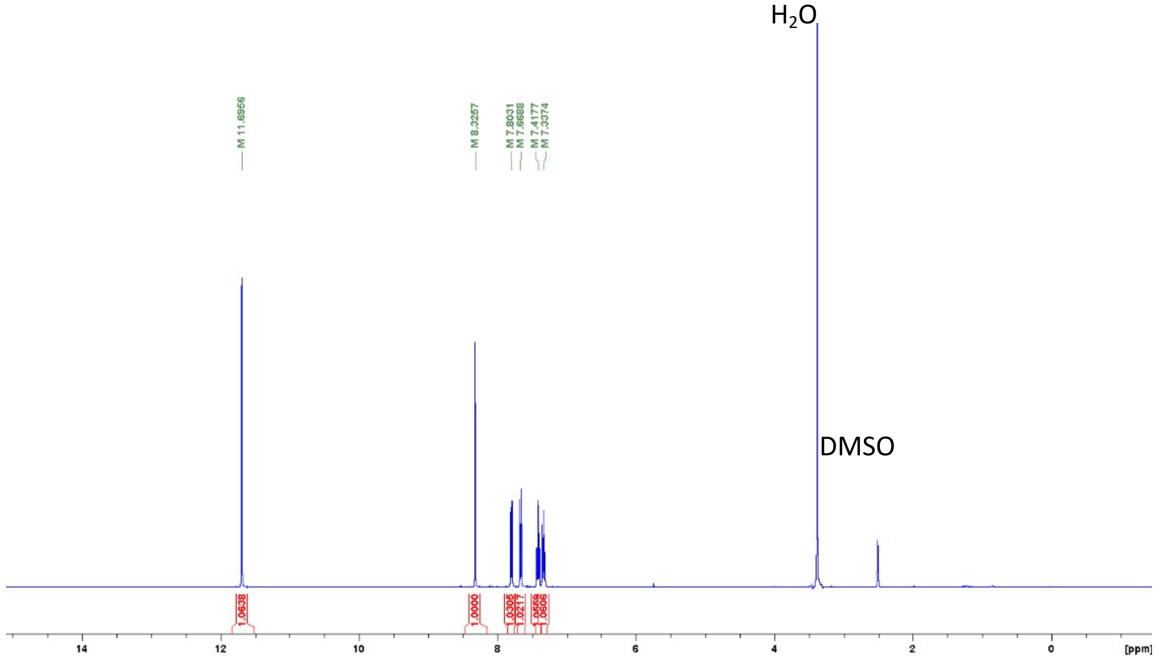

^{13}C NMR of **4d** in CD_3SOCD_3:

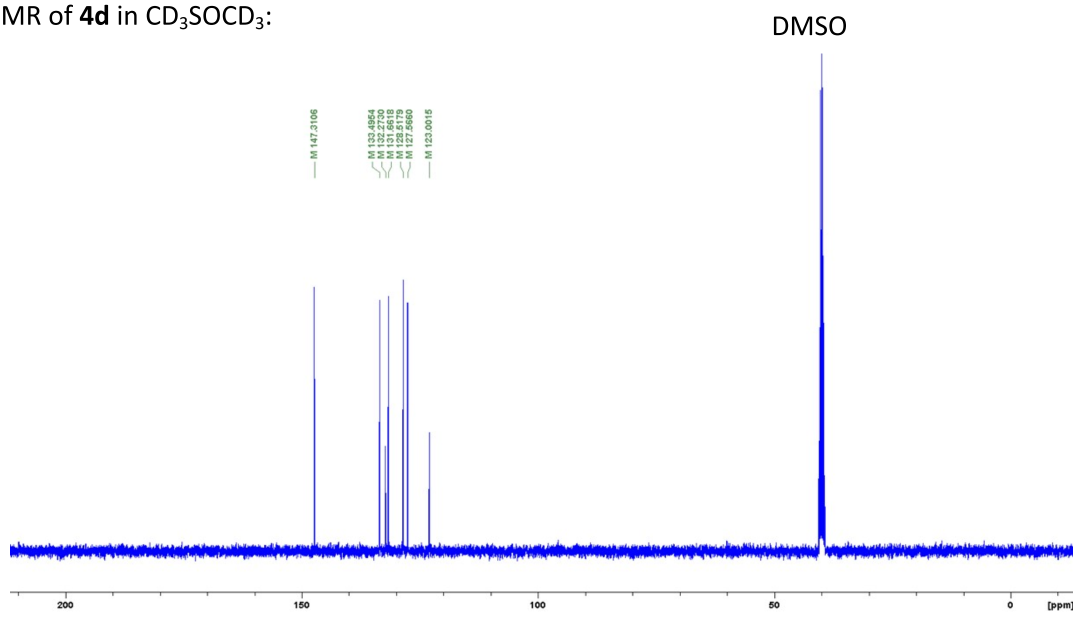

N-[(4-Chlorophenyl)methylidene]hydroxylamine **4e**

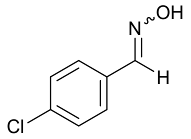

Prepared from 4-chlorobenzaldehyde using **GP1**. Thick colourless oil (584 mg, 75%).

^{1}H NMR (400 MHz, $CDCl_3$) δ 8.14 (s, 1H and br, 1H, OH), 7.53 (d, *J* = 8.5 Hz, 2H), 7.38 (d, *J* = 8.5 Hz, 2H).

^{13}C NMR (100 MHz, $CDCl_3$) δ 149.3, 136.0, 130.4, 129.1, 128.2.

HRMS (ESI) *m/z*: [M+H$^+$] calculated for C_7H_6ClNO: 156.0211; found: 156.0222.

Analytical data are consistent with those previously reported.[20]

^{1}H NMR of **4e** in $CDCl_3$:

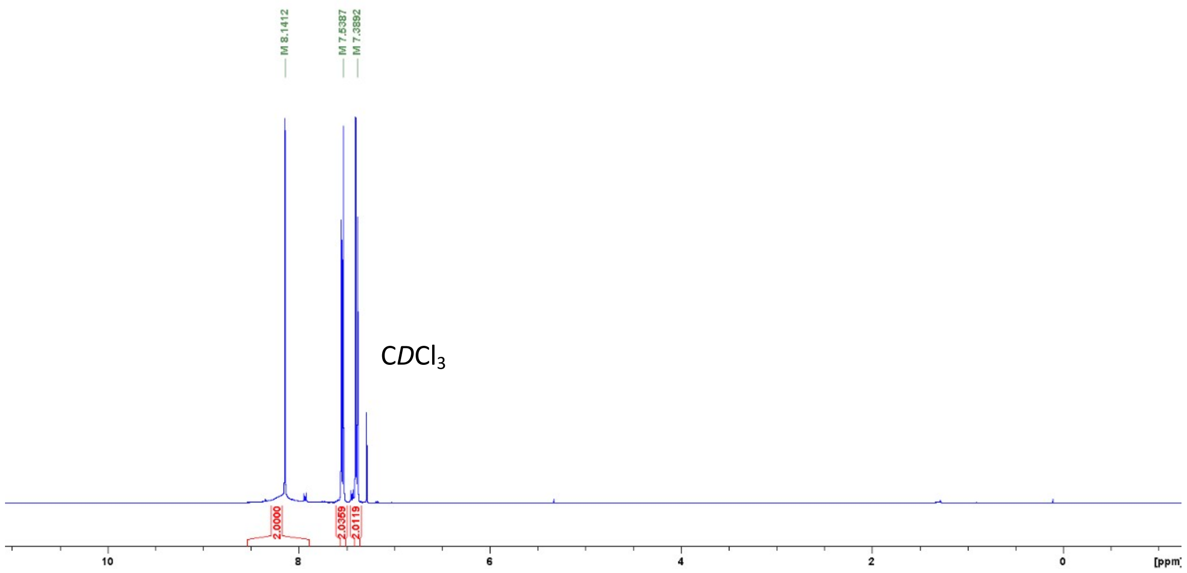

^{13}C NMR of **4e** in $CDCl_3$:

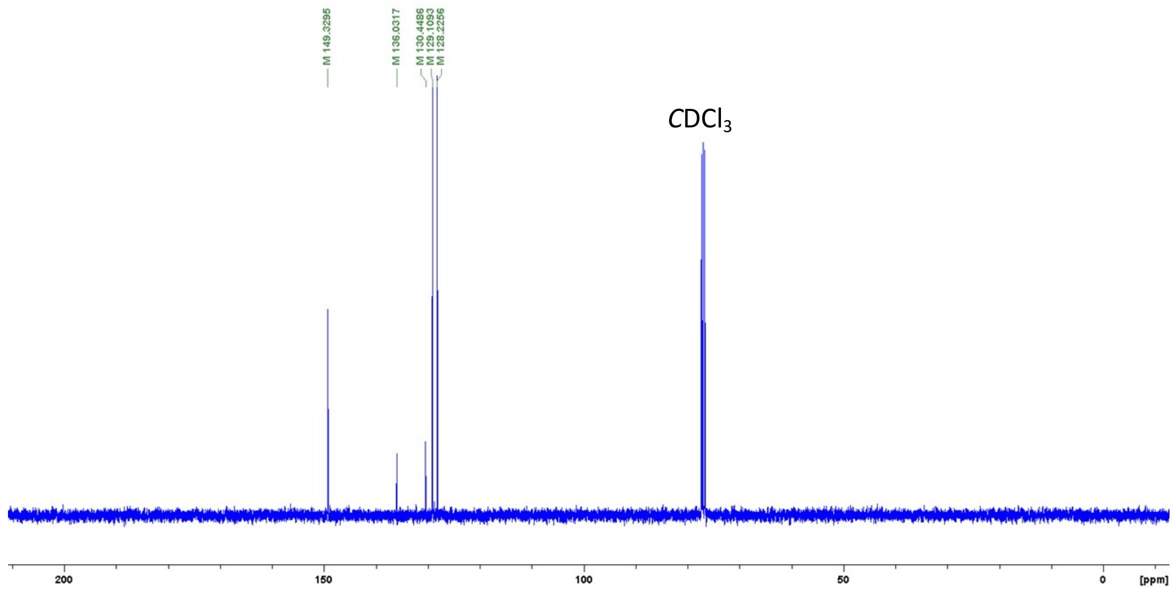

N-[(4-Fluorophenyl)methylidene]hydroxylamine **4f**

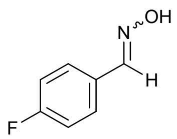

Prepared from 4-fluorobenzaldehyde using **GP1**. Thick colourless oil (577 mg, 83%).

^{1}H NMR (400 MHz, $CDCl_3$) δ 8.39 (br, 1H, OH), 8.15 (s, 1H), 7.59 (m, 2H), 7.10 (m, 2H).

^{13}C NMR (100 MHz, $CDCl_3$) δ 163.7 (d, *J* = 250.5 Hz), 149.2, 128.8 (d, *J* = 8.3 Hz), 128.1 (d, *J* = 3.2 Hz), 115.9 (d, *J* = 21.9 Hz).

^{19}F NMR (400 MHz, $CDCl_3$) δ -110.0.

HRMS (ESI) *m/z*: [M+H$^+$] calculated for C_7H_6FNO: 140.0506; found: 140.0517.

Analytical data are consistent with those previously reported.[20]

^{1}H NMR of **4f** in $CDCl_3$:

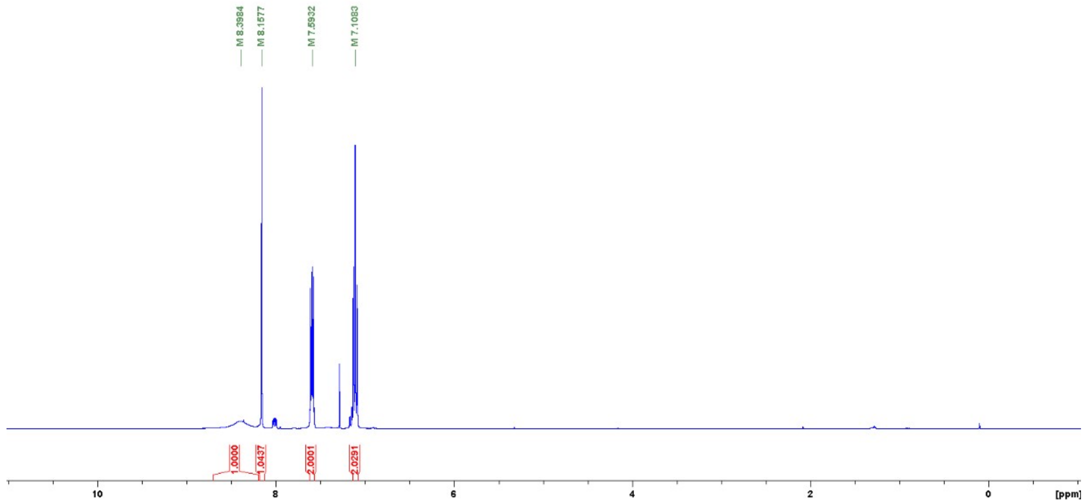

^{13}C NMR of **4f** in $CDCl_3$:

*C*DCl$_3$

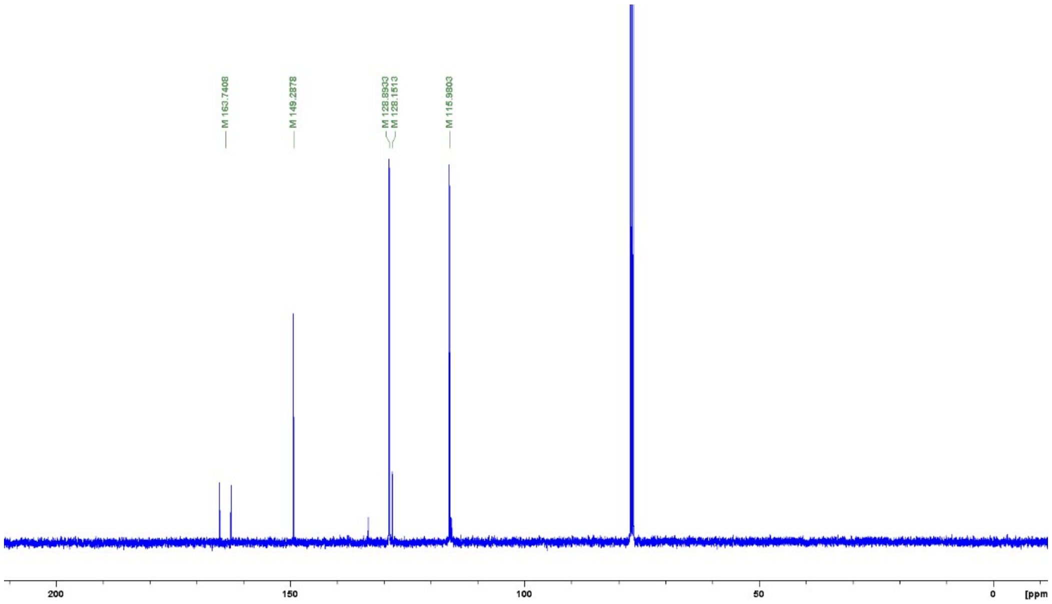

^{13}C DEPT-135 NMR of **4f** in $CDCl_3$:

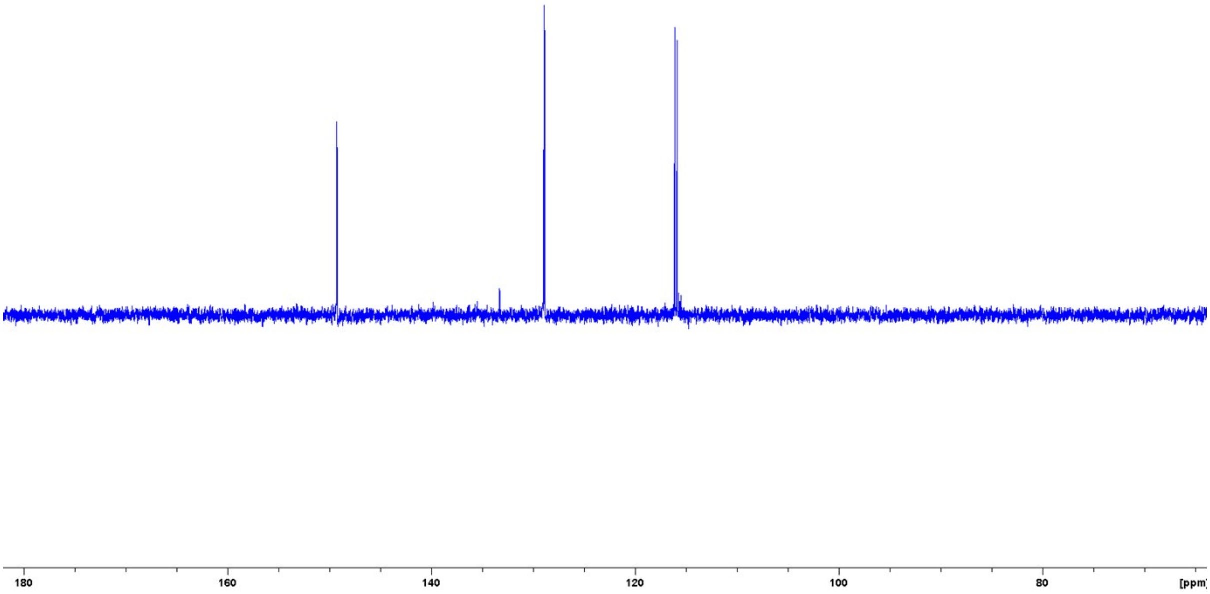

^{19}F NMR of **4f** in $CDCl_3$:

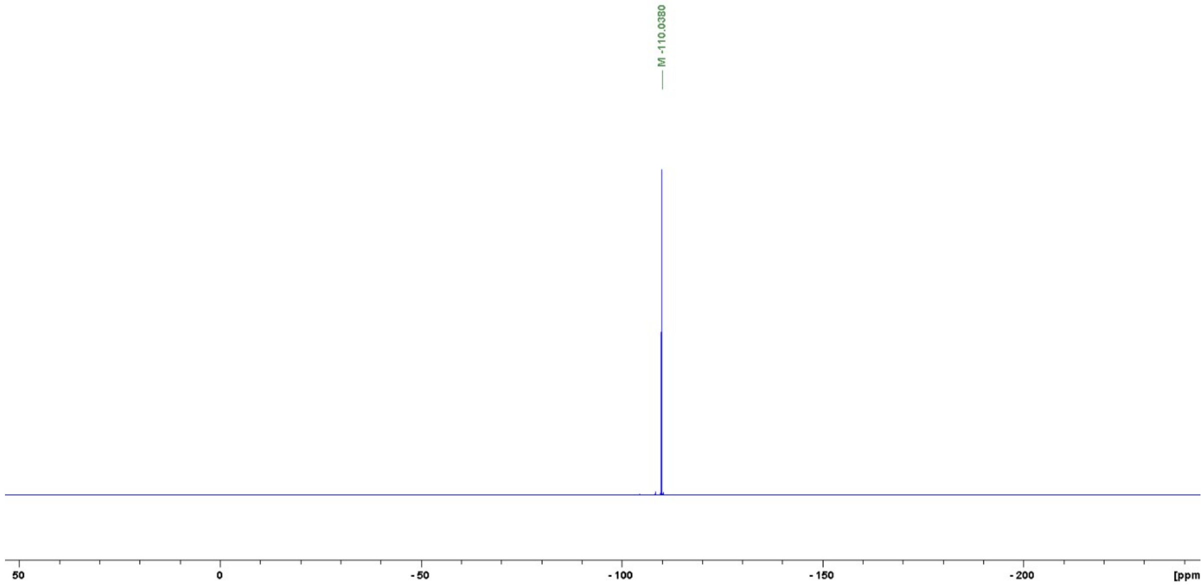
M -110.0380
50
0
- 50
- 100
- 150
- 200
[ppm

N-[(4-Bromophenyl)methylidene]hydroxylamine **4g**

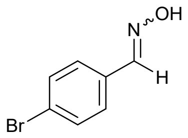

Prepared from 4-bromobenzaldehyde using **GP1**. Thick colourless oil (780 mg, 78%).

^{1}H NMR (400 MHz, CD_3SOCD_3) δ 11.40 (s, 1H), 8.13 (s, 1H), 7.59 (d, *J* = 8.5 Hz, 2H), 7.53 (d, *J* = 8.5 Hz, 2H).

^{13}C NMR (100 MHz, CD_3SOCD_3) δ 147.7, 132.7, 132.1, 128.7, 122.8.

HRMS (ESI) *m/z*: [M+H$^+$] calculated for C_7H_6BrNO: 199.9704; found: 199.9719.

Analytical data are consistent with those previously reported.[20]

^{1}H NMR of **4g** in CD_3SOCD_3:

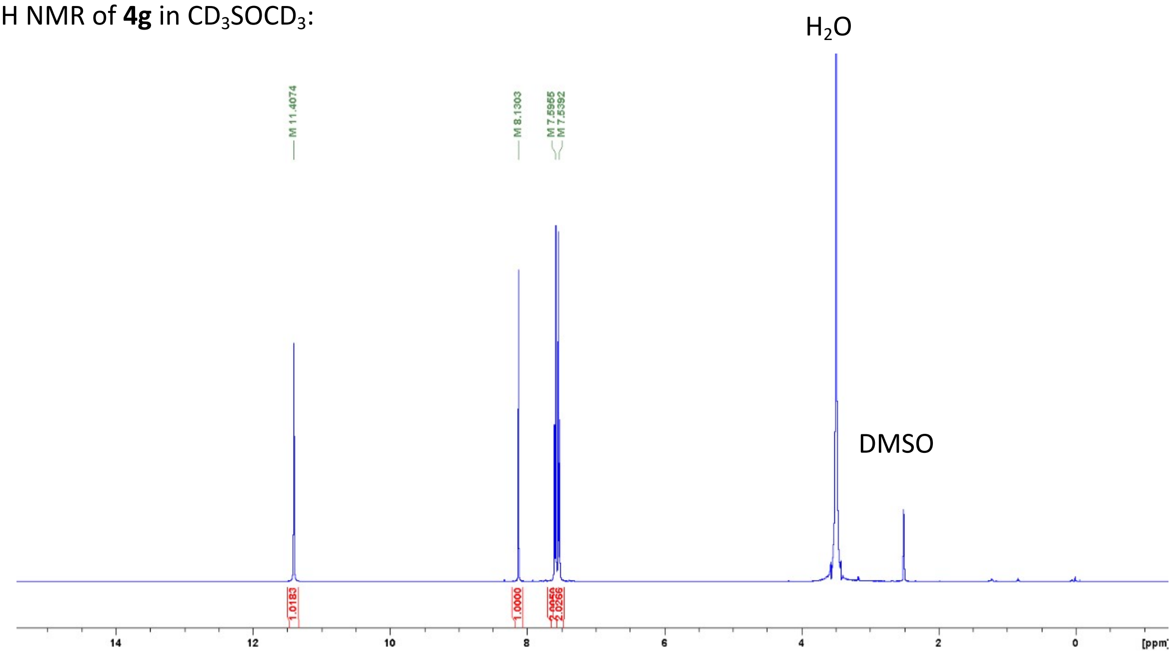

^{13}C NMR of **4g** in CD_3SOCD_3:

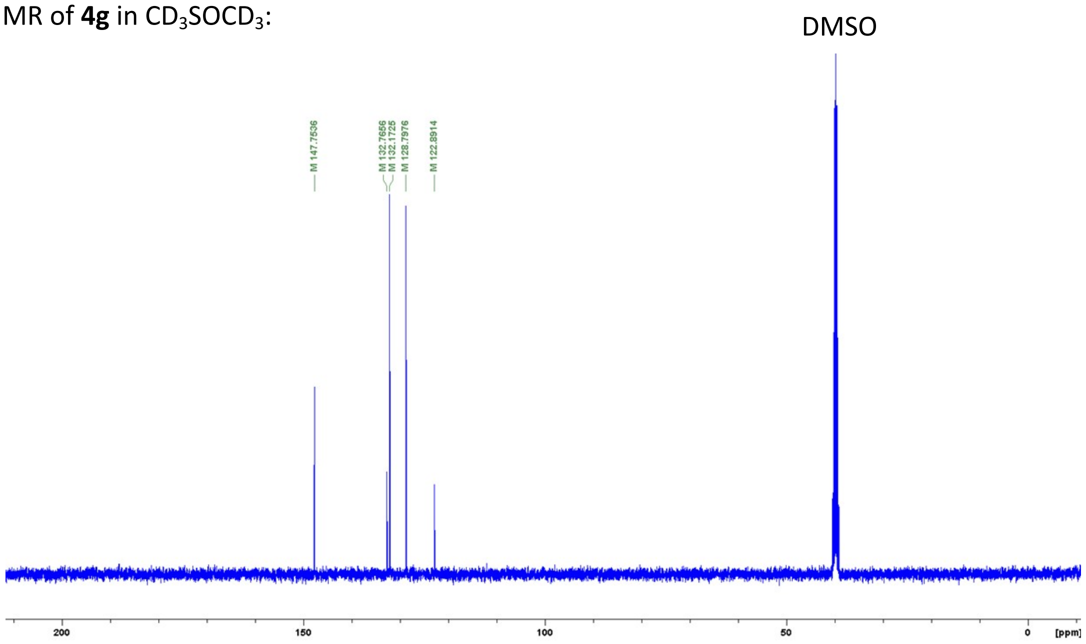

N-[(2-Methylphenyl)methylidene]hydroxylamine **4h**

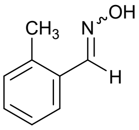

Prepared from 2-methylbenzaldehyde using **GP1**. Thick pale yellow oil (540 mg, 80%).

^{1}H NMR (400 MHz, $CDCl_3$) δ 8.70 (br, 1H, OH), 8.46 (s, 1H), 7.70 (dd, *J* = 1.2, 7.8 Hz, 1H), 7.32 (td, *J* = 1.5, 7.8, 7.8 Hz, 1H), 7.22-7.28 (m, 2H), 2.46 (s, 3H).

^{13}C NMR (100 MHz, $CDCl_3$) δ 149.3, 136.8, 130.8, 130.2, 129.9, 126.7, 126.2, 19.7.

HRMS (ESI) *m/z*: [M+H$^+$] calculated for C_8H_9NO: 136.0757; found: 136.0766.

Analytical data are consistent with those previously reported.[19]

^{1}H NMR of **4h** in $CDCl_3$:

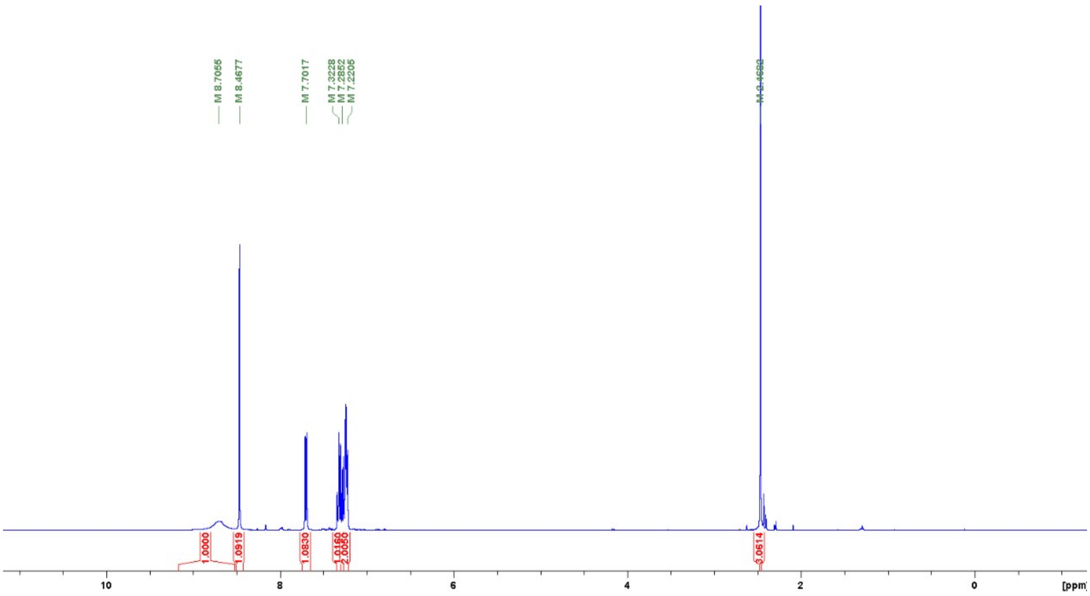

^{13}C NMR of **4h** in $CDCl_3$:

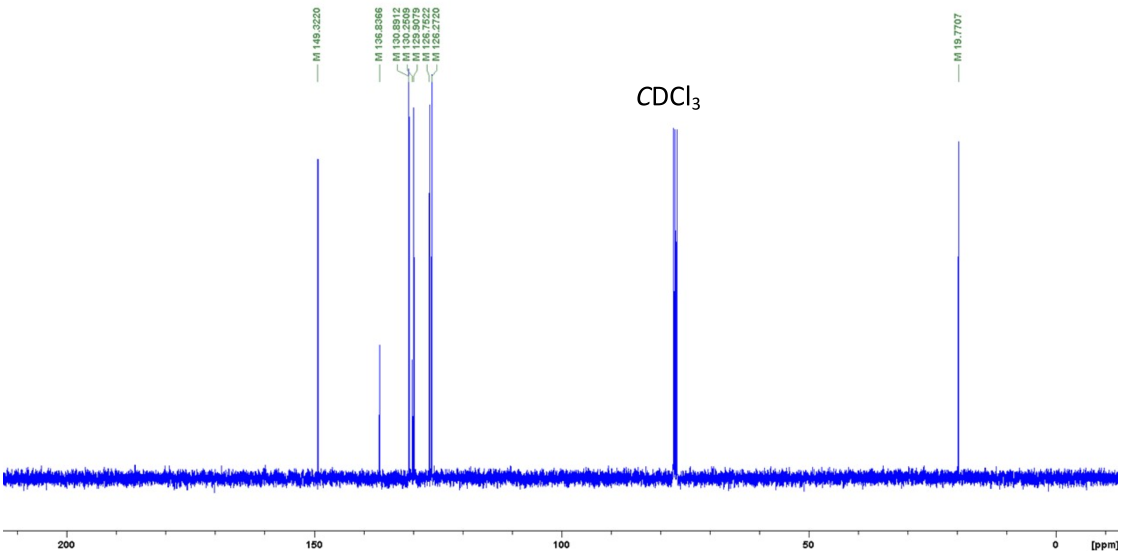

N-[(4-tert-Butylphenyl)methylidene]hydroxylamine **4i**

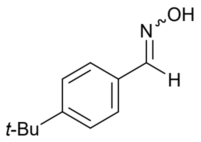

Prepared from 4-*tert*-butylbenzaldehyde using **GP1**. Thick colourless oil (780 mg, 78%).

^{1}H NMR (400 MHz, CD_3SOCD_3) δ 11.15 (s, 1H), 8.09 (s, 1H), 7.51 (d, *J* = 8.3 Hz, 2H), 7.40 (d, *J* = 8.3 Hz, 2H), 1.26 (s, 9H).

^{13}C NMR (100 MHz, CD_3SOCD_3) δ 152.4, 148.4, 130.7, 126.6, 125.9, 34.9, 31.4.

HRMS (ESI) *m/z*: [M+H^{+}] calculated for $C_{11}H_{15}NO$: 178.1226; found: 178.1232.

Analytical data are consistent with those previously reported.[20]

^{1}H NMR of **4i** in CD_3SOCD_3:

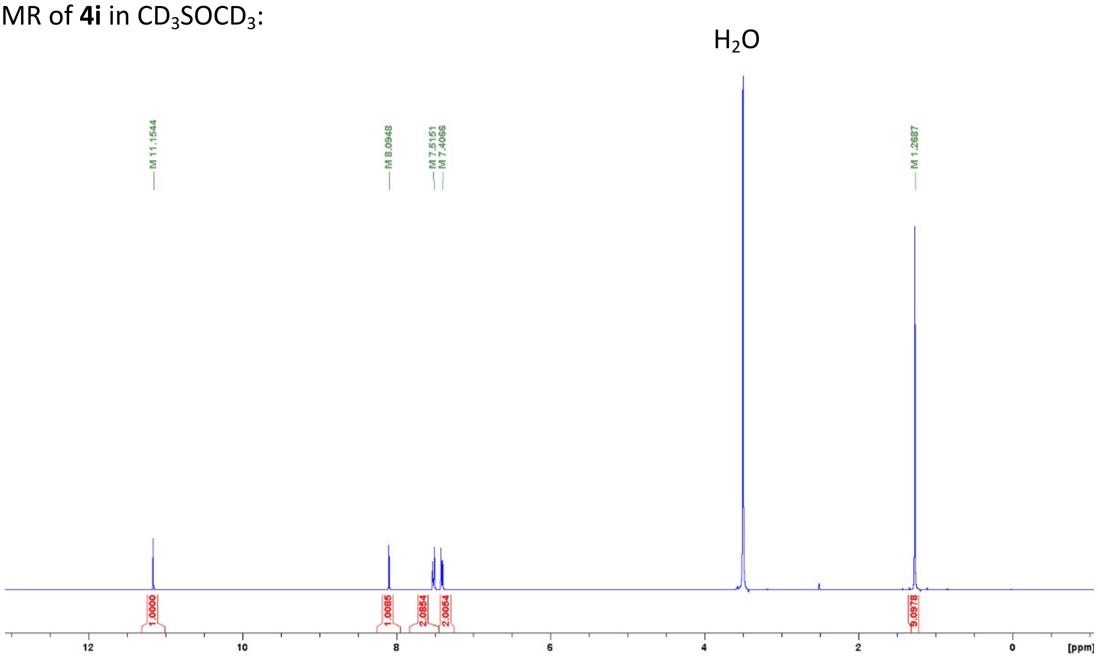

^{13}C NMR of **4i** in CD_3SOCD_3:

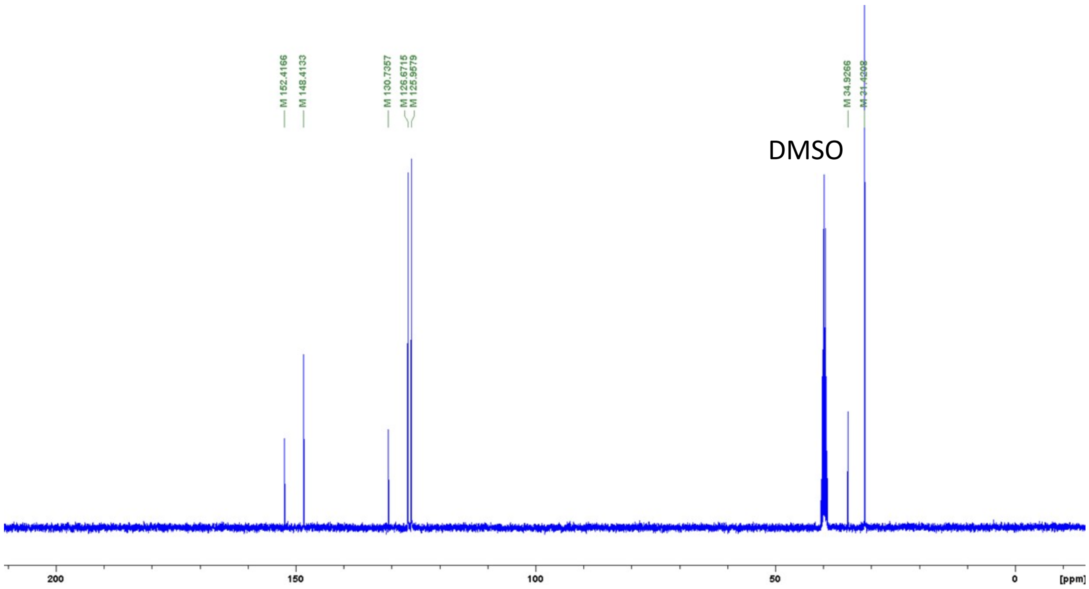

N-Benzylhydroxylamine **5b**

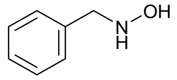

Prepared from **oxime 4b** using **GP2**. Thick colourless oil (203 mg, 55%).

^{1}H NMR (400 MHz, CD_3SOCD_3) δ 7.21-7.35 (m, 6H), 5.97 (br, 1H, OH), 3.87 (s, 2H).

^{13}C NMR (100 MHz, CD_3SOCD_3) δ 139.5, 129.1, 128.4, 127.0, 57.9.

HRMS (ESI) *m/z*: [M+H^{+}] calculated for C_7H_9NO: 124.0757; found: 124.0766.

Analytical data are consistent with those previously reported.[19]

^{1}H NMR of **5b** in CD_3SOCD_3:

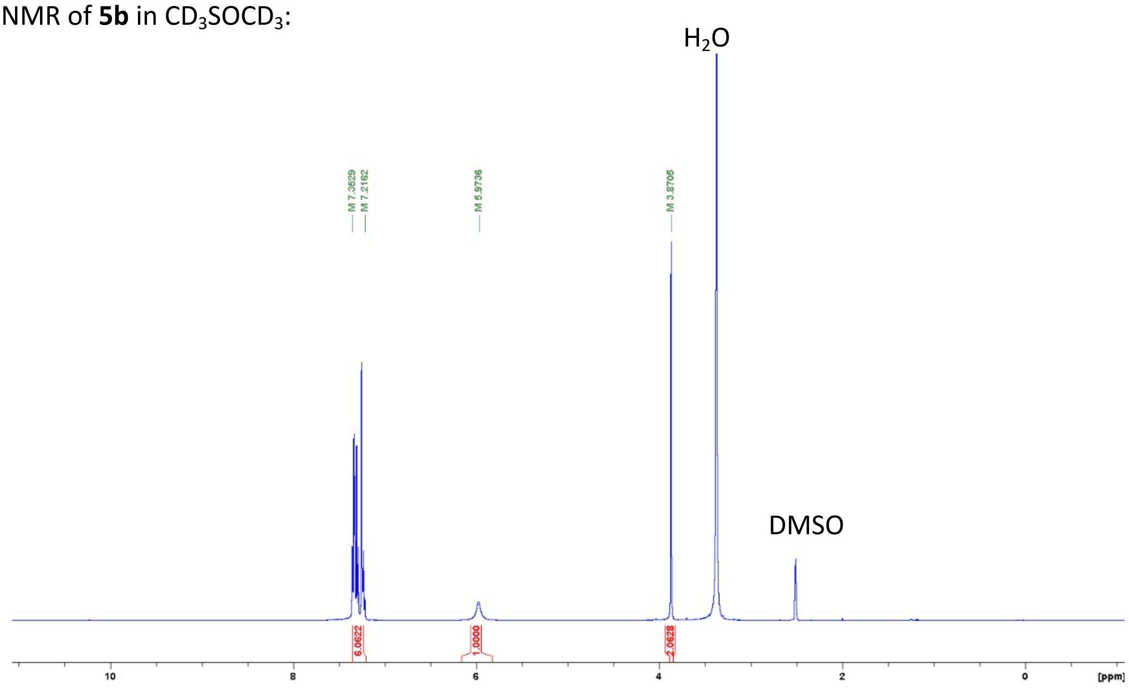

^{13}C NMR of **5b** in CD_3SOCD_3:

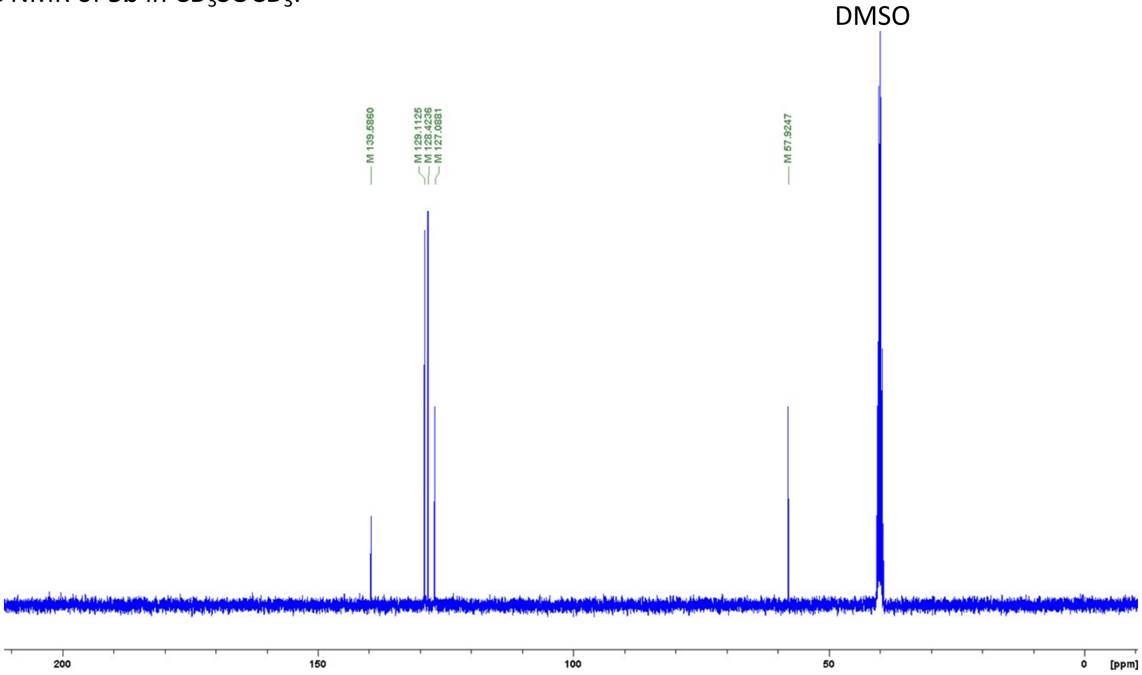

N-[(2-Fluorophenyl)methyl]hydroxylamine **5c**

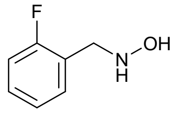

Prepared from **oxime 4c** using **GP2**. Thick colourless oil (212 mg, 50%).

^{1}H NMR (400 MHz, $CDCl_3$) δ 7.36 (td, *J* = 1.6, 7.4, 7.4 Hz, 1H), 7.29 (m, 1H), 7.13 (td, *J* = 1.1, 7.4, 7.4 Hz, 1H), 7.08 (m, 1H), 5.96 (br, 2H, NH and OH), 4.08 (s, 2H).

^{13}C NMR (100 MHz, $CDCl_3$) δ 161.4 (d, *J* = 160 Hz), 131.6 (d, *J* = 4.3 Hz), 129.5 (d, *J* = 8.2 Hz), 124.0 (d, *J* = 3.5 Hz), 123.9 (d, *J* = 11.8 Hz), 115.4 (d, *J* = 21.6 Hz), 51.7 (d, *J* = 2.6 Hz).

^{19}F NMR (400 MHz, $CDCl_3$) δ -119.8.

HRMS (ESI) *m/z*: [M+H$^+$] calculated for C_7H_8FNO: 142.0662; found: 142.0671.

Analytical data are consistent with those previously reported.[19]

^{1}H NMR of **5c** in $CDCl_3$:

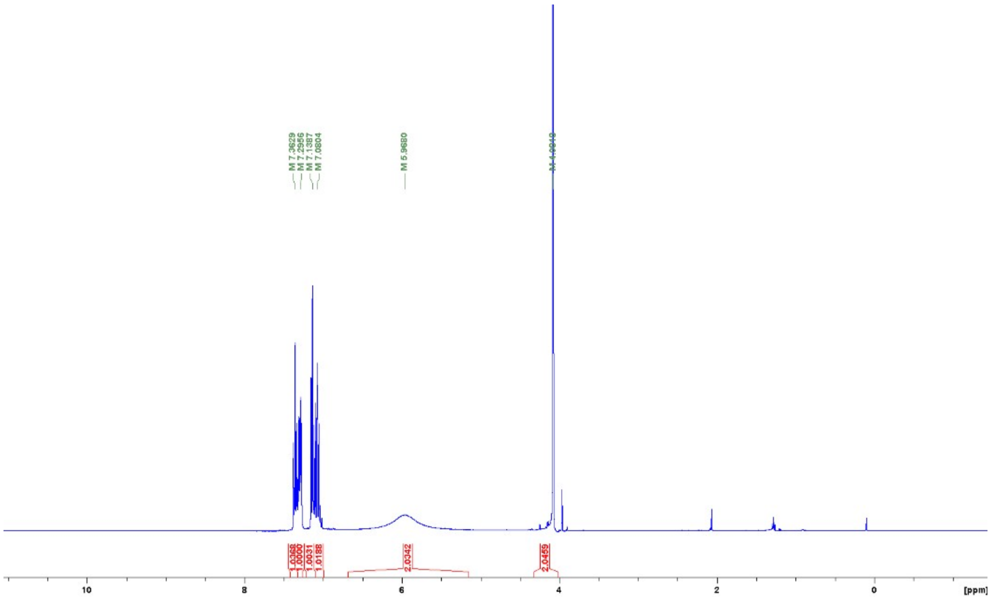

^{13}C NMR of **5c** in $CDCl_3$:

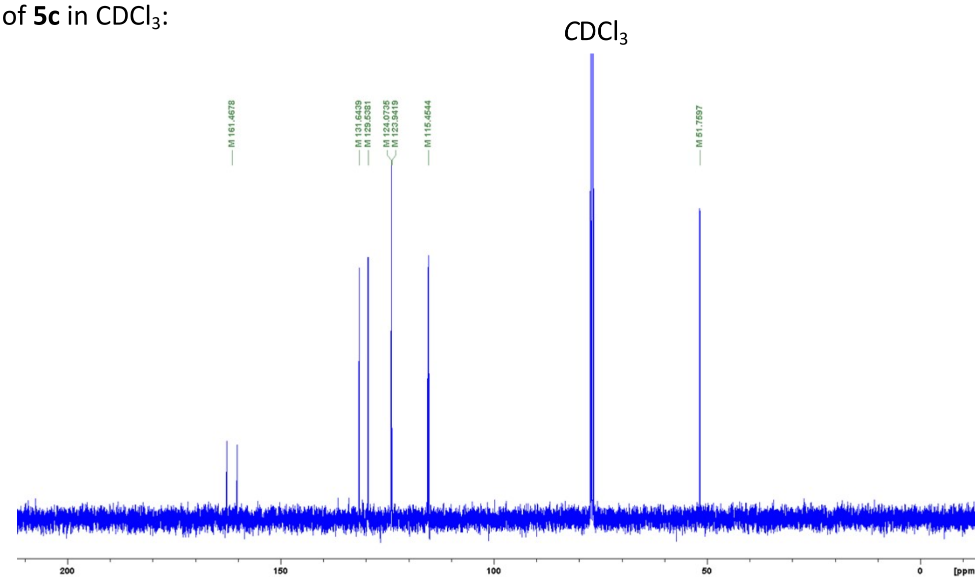

^{13}C DEPT-135 NMR of **5c** in $CDCl_3$:

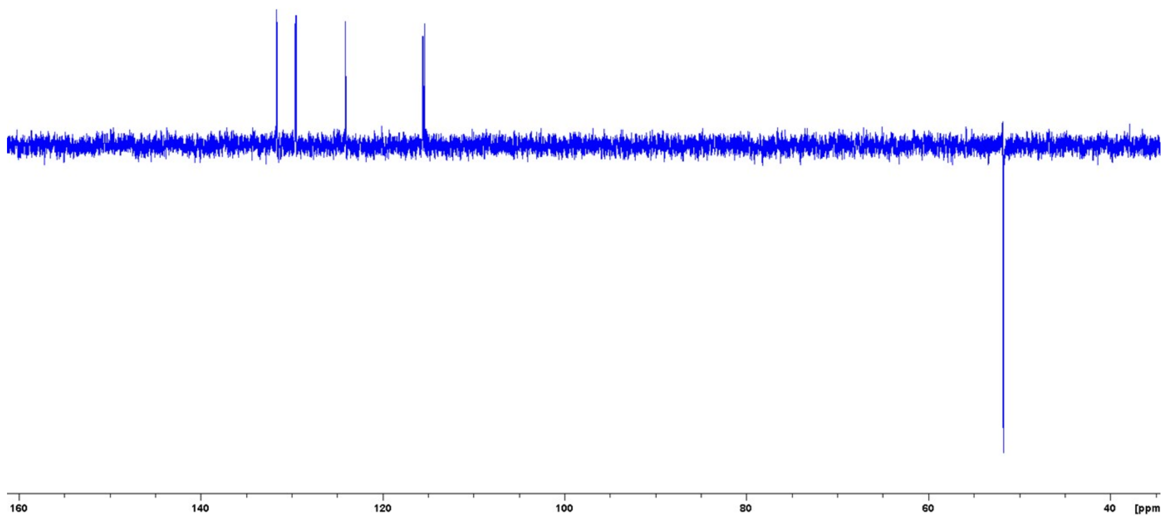

^{19}F NMR of **5c** in $CDCl_3$:

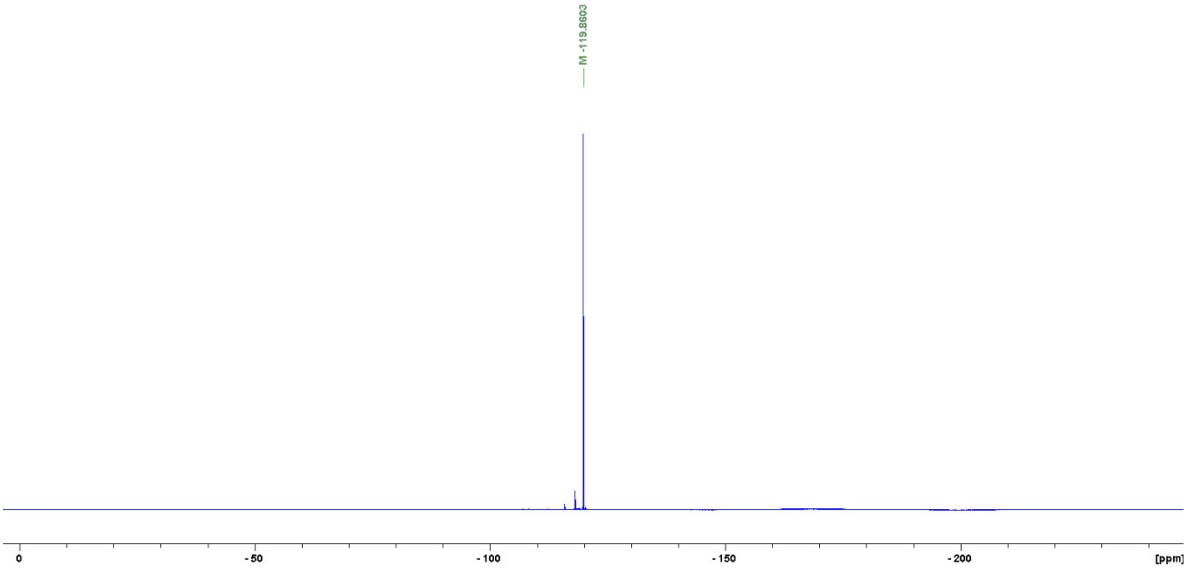

N-[(2-Bromophenyl)methyl]hydroxylamine **5d**

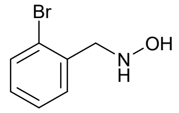

Prepared from **oxime 4d** using **GP2**. Thick colourless oil (400 mg, 66%).

^{1}H NMR (400 MHz, CD_3SOCD_3) δ 7.56 (m, 2H), 7.39 (s, 1H), 7.35 (td, *J* = 1.1, 7.7, 7.7 Hz, 1H), 7.19 (td, *J* = 1.8, 7.7, 7.7 Hz, 1H), 6.10 (br, 1H, OH), 3.94 (s, 2H).

^{13}C NMR (100 MHz, CD_3SOCD_3) δ 138.5, 132.5, 131.0, 129.1, 127.8, 123.8, 57.3.

HRMS (ESI) *m/z*: [M+H$^+$] calculated for C_7H_8BrNO: 201.9862; found: 201.9869.

Analytical data are consistent with those previously reported.[19]

^{1}H NMR of **5d** in CD_3SOCD_3:

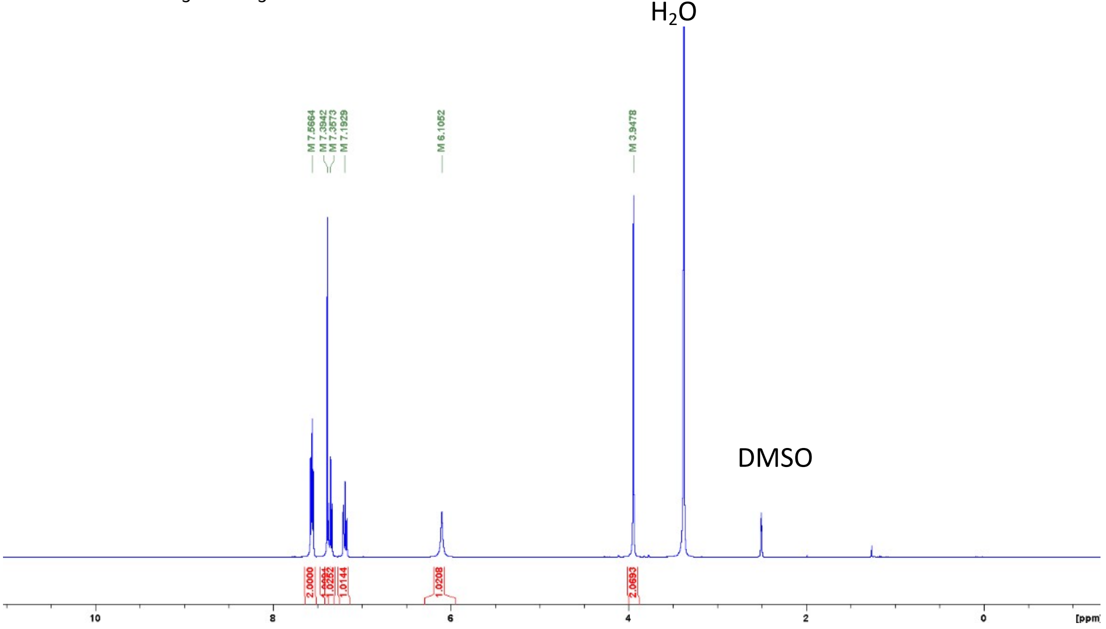

^{13}C NMR of **5d** in CD_3SOCD_3:

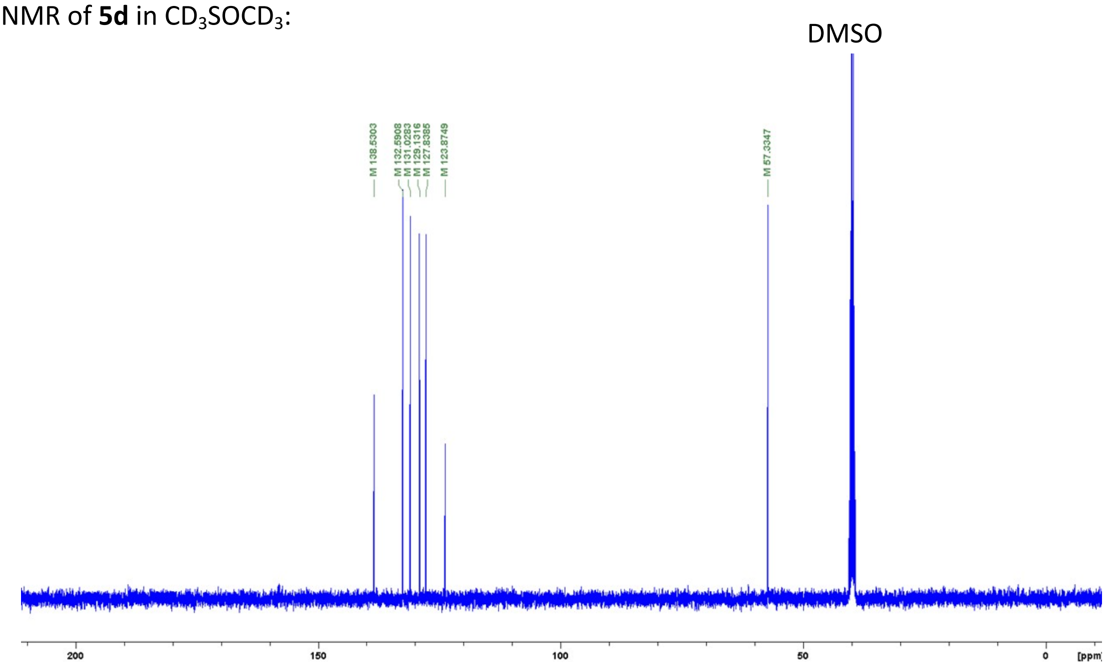

N-[(4-Chlorophenyl)methyl]hydroxylamine **5e**

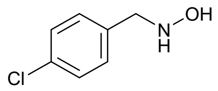

Prepared from **oxime 4e** using **GP2**. Thick colourless oil (307 mg, 65%).

^{1}H NMR (400 MHz, $CDCl_3$) δ 7.33 (d, *J* = 8.6 Hz, 2H), 7.27 (d, *J* = 8.6 Hz, 2H), 5.85 (br, 2H, NH and OH), 3.97 (s, 2H).

^{13}C NMR (100 MHz, $CDCl_3$) δ 135.6, 133.5, 130.5, 128.8, 57.3.

HRMS (ESI) *m/z*: [M+H$^+$] calculated for C_7H_8ClNO: 158.0367; found: 158.0376.

Analytical data are consistent with those previously reported.[21]

^{1}H NMR of **5e** in $CDCl_3$:

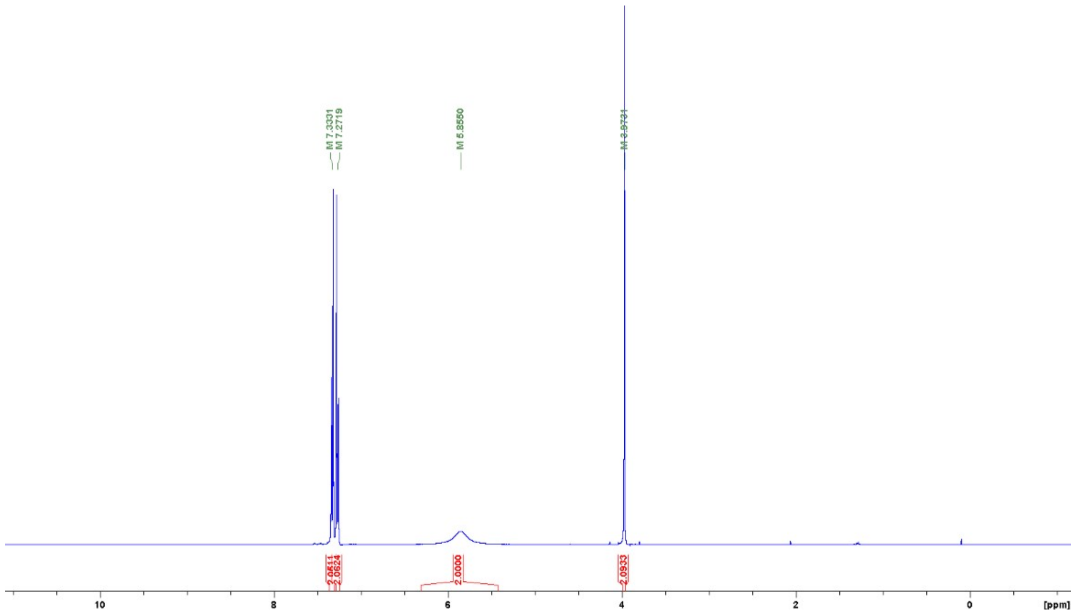

^{13}C NMR of **5e** in $CDCl_3$:

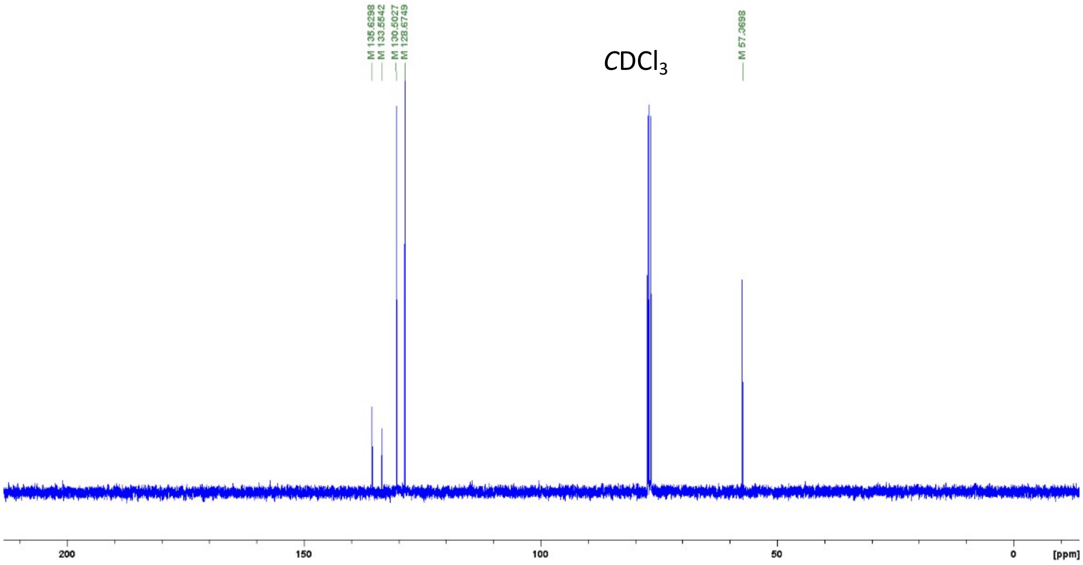

N-[(4-Fluorophenyl)methyl]hydroxylamine **5f**

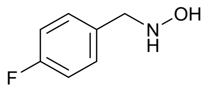

Prepared from **oxime 4f** using **GP2**. Thick colourless oil (300 mg, 73%).

^{1}H NMR (400 MHz, $CDCl_3$) δ 7.30 (m, 2H), 7.04 (m, 2H), 6.01 (br, 2H, NH and OH), 3.96 (s, 2H).

^{13}C NMR (100 MHz, $CDCl_3$) δ 162.3 (d, *J* = 246.4 Hz), 132.7 (d, *J* = 3.3 Hz), 130.8 (d, *J* = 8.2 Hz), 115.3 (d, *J* = 21.3 Hz), 57.3.

^{19}F NMR (400 MHz, $CDCl_3$) δ -114.8.

HRMS (ESI) *m/z*: [M+H$^+$] calculated for C_7H_8FNO: 142.0663; found: 142.0669.

Analytical data are consistent with those previously reported.[21]

^{1}H NMR of **5f** in $CDCl_3$:

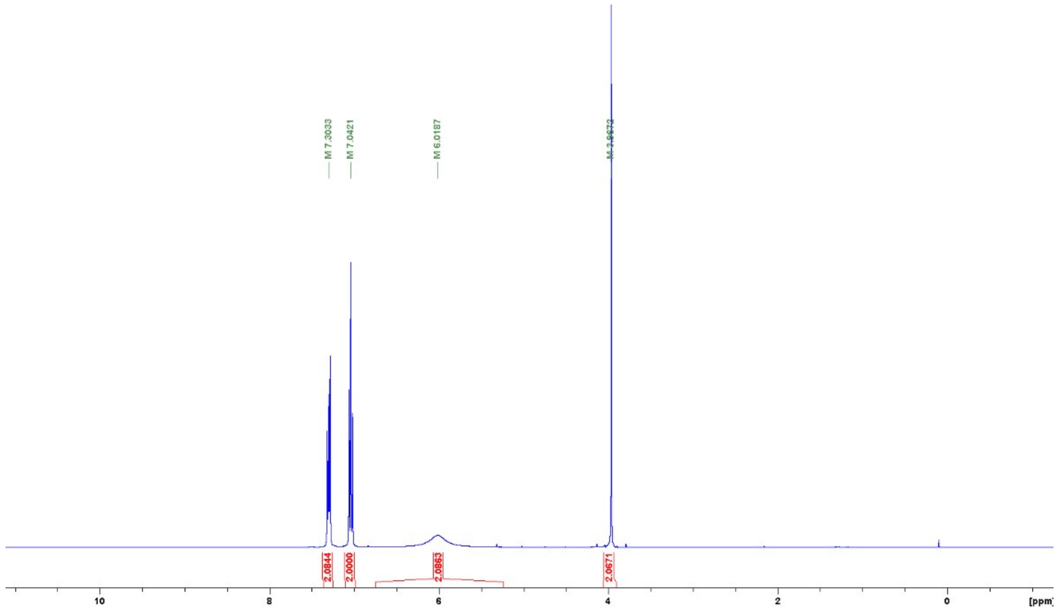

^{13}C NMR of **5f** in $CDCl_3$:

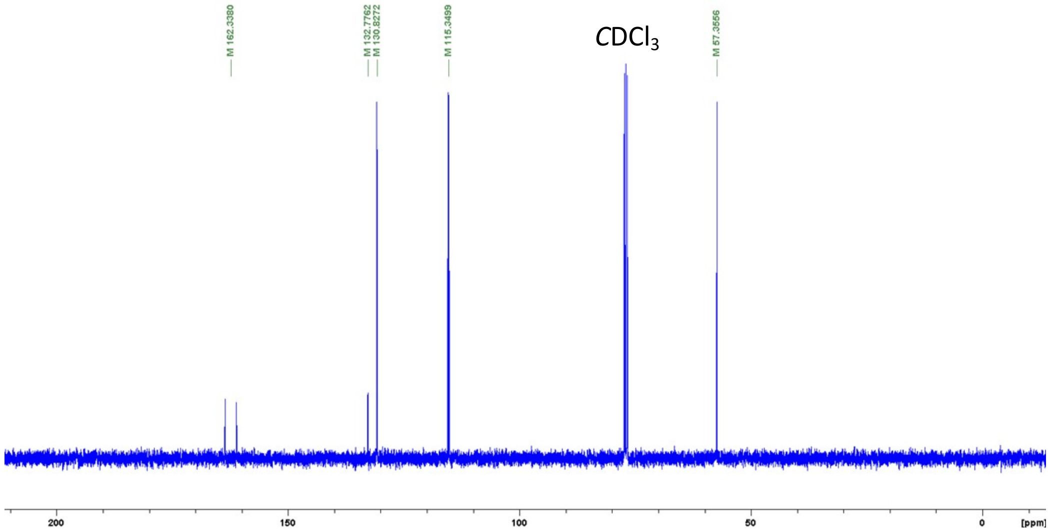

^{13}C DEPT-135 NMR of **5f** in $CDCl_3$:

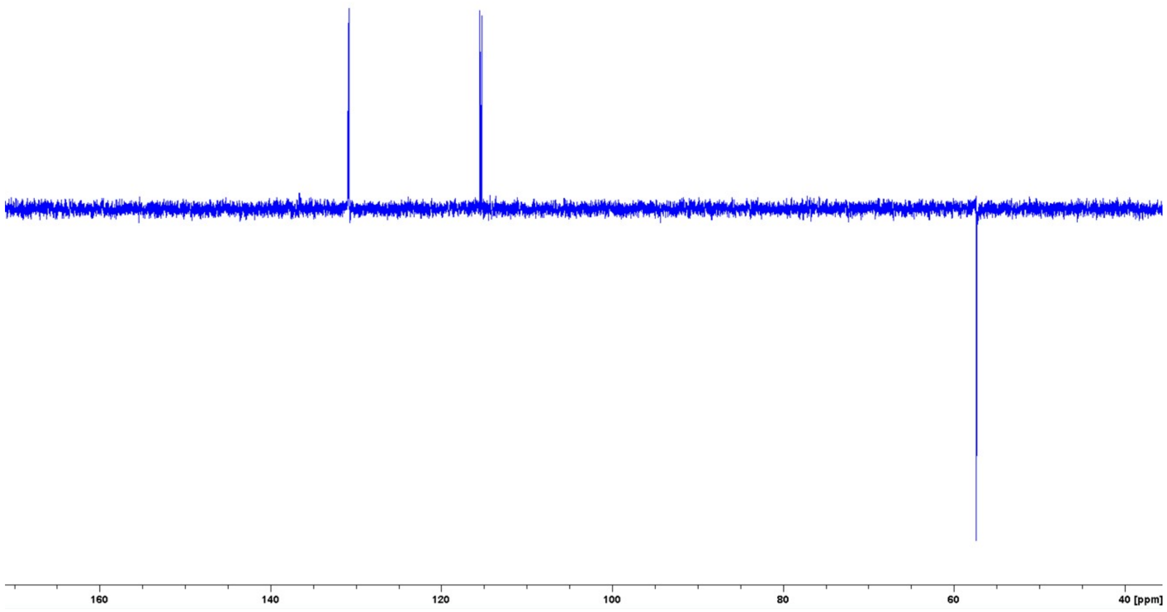

^{19}F NMR of **5f** in $CDCl_3$:

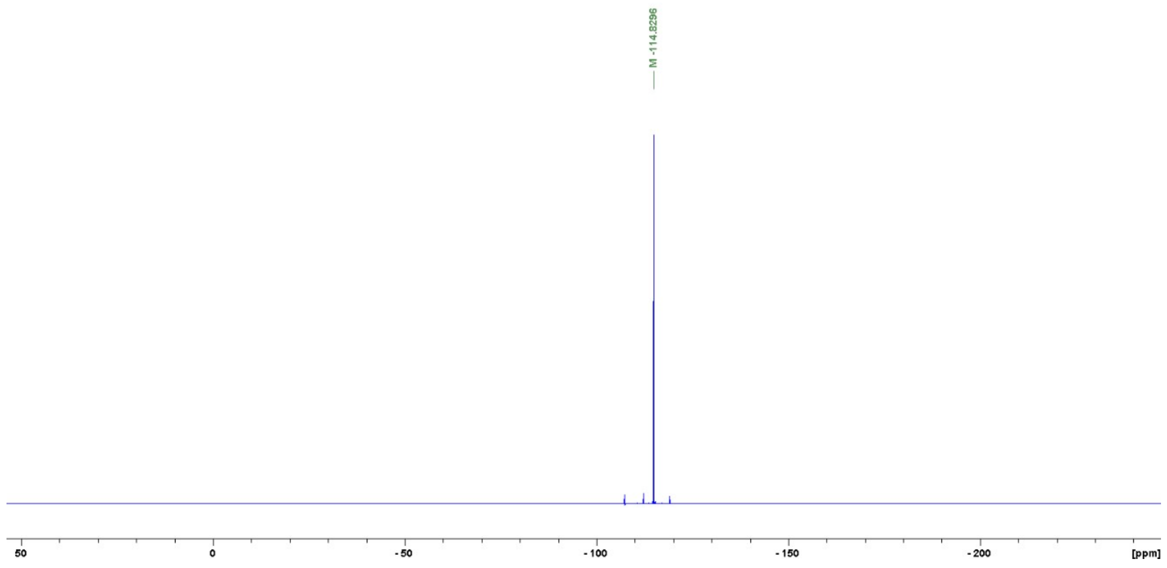

N-[(4-Bromophenyl)methyl]hydroxylamine **5g**

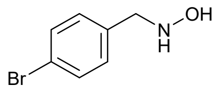

Prepared from **oxime 4g** using **GP2**. Thick colourless oil (394 mg, 65%).

^{1}H NMR (400 MHz, CD_3SOCD_3) δ 7.48 (d, *J* = 8.3 Hz, 2H), 7.30 (d, *J* = 8.3 Hz, 2H), 7.28 (s, 1H, NH), 6.05 (br, 1H, OH), 3.83 (s, 2H).

^{13}C NMR (100 MHz, CD_3SOCD_3) δ 139.3, 131.27, 131.23, 120.1, 57.0.

HRMS (ESI) *m/z*: [$M+H^+$] calculated for C_7H_8BrNO: 201.9862; found: 201.9868.

Analytical data are consistent with those previously reported.[21]

^{1}H NMR of **5g** in CD_3SOCD_3:

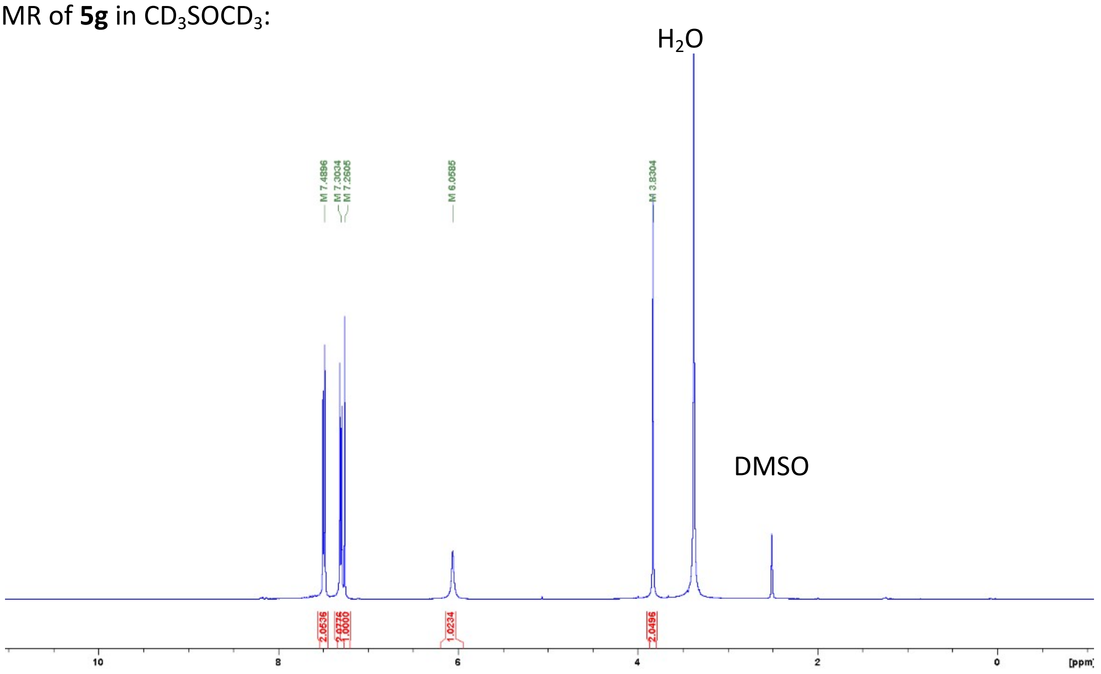

^{13}C NMR of **5g** in CD_3SOCD_3:

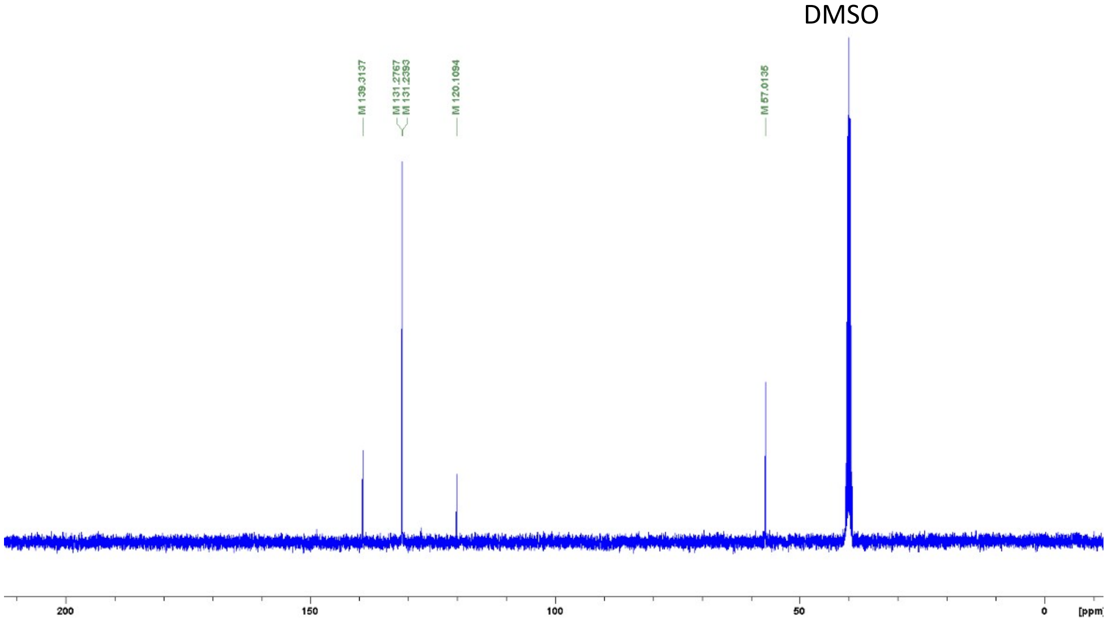

N-[(2-Methylphenyl)methyl]hydroxylamine **5h**

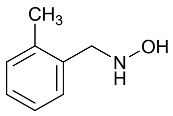

Prepared from **oxime 4h** using **GP2**. Thick colourless oil (243 mg, 59%).

^{1}H NMR (400 MHz, $CDCl_3$) δ 7.17-7.29 (m, 4H), 5.97 (br, 2H, NH and OH), 4.05 (s, 2H), 2.38 (s, 3H).

^{13}C NMR (100 MHz, $CDCl_3$) δ 137.1, 134.6, 130.4, 130.0, 127.8, 126.0, 55.7, 19.0.

HRMS (ESI) *m/z*: [M+H^{+}] calculated for $C_8H_{11}NO$: 138.0913; found: 138.0919.

Analytical data are consistent with those previously reported.[19]

^{1}H NMR of **5h** in $CDCl_3$:

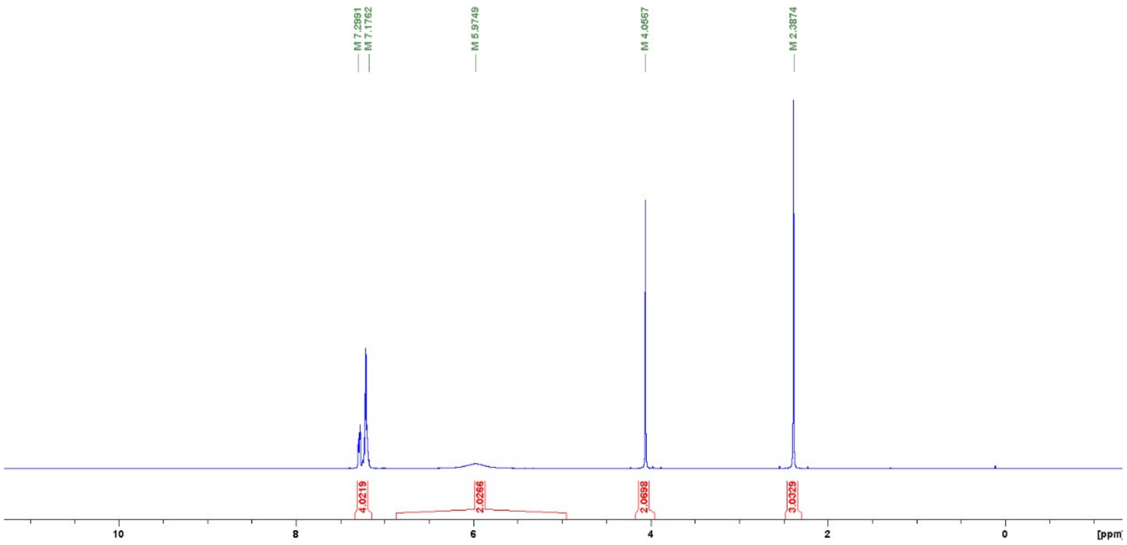

^{13}C NMR of **5h** in $CDCl_3$:

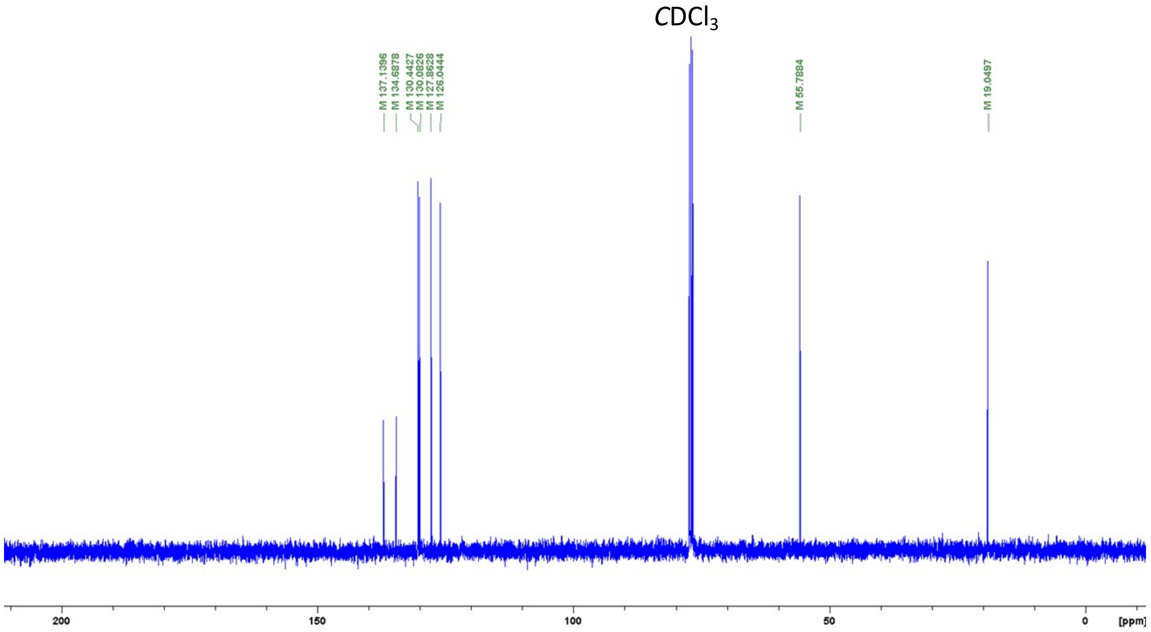

N-[(4-tert-Butylphenyl)methyl]hydroxylamine **5i**

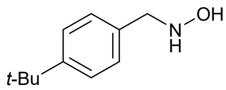

Prepared from **oxime 4i** using **GP2**. Thick colourless oil (311 mg, 58%).

^{1}H NMR (400 MHz, $CDCl_3$) δ 7.39 (d, *J* = 8.2 Hz, 2H), 7.27 (d, *J* = 8.2 Hz, 2H), 5.72 (br, 2H, NH and OH), 3.98 (s, 2H), 1.33 (s, 9H).

^{13}C NMR (100 MHz, $CDCl_3$) δ 150.6, 133.7, 128.9, 125.4, 57.8, 34.5, 31.3.

HRMS (ESI) *m/z*: [M+H$^+$] calculated for $C_{11}H_{17}NO$: 180.1383; found: 180.1379.

Analytical data are consistent with those previously reported.[21]

^{1}H NMR of **5i** in $CDCl_3$:

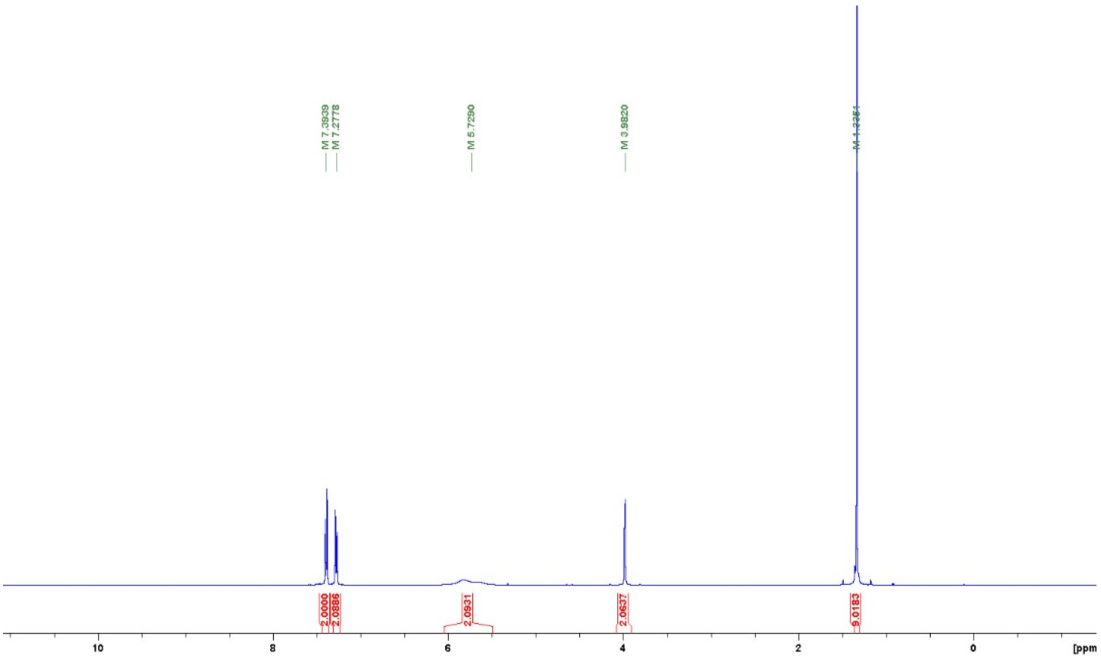

^{13}C NMR of **5i** in $CDCl_3$:

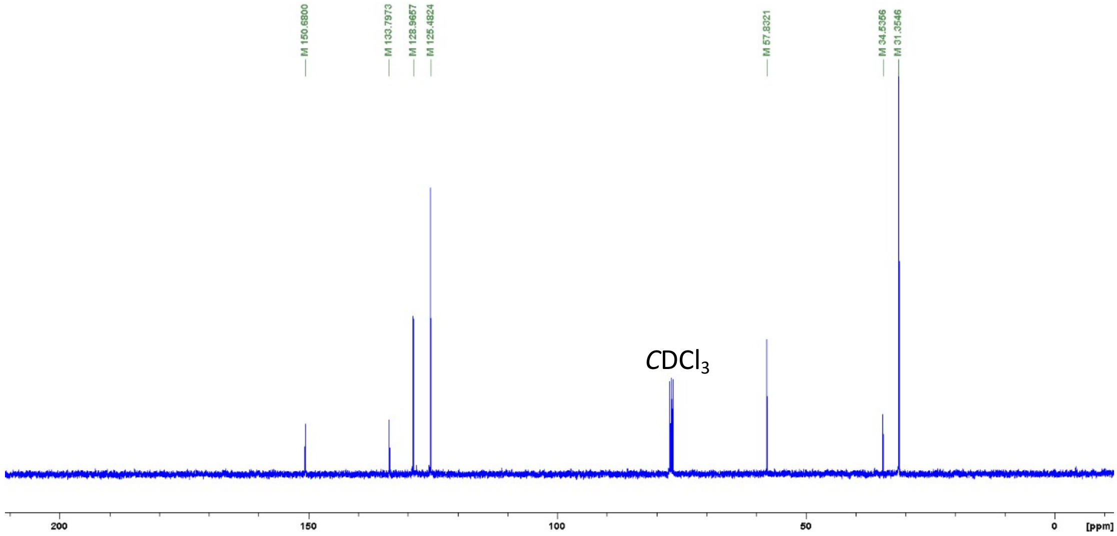

2-Benzyl-4,4-dimethyl-1,2-oxazolidine-3,5-dione **2b**

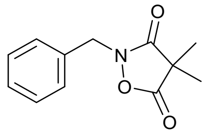

Prepared from **hydroxylamine 5b** using **GP3**. Colourless oil (169 mg, 77%).

^{1}H NMR (400 MHz, $CDCl_3$) δ 7.34-7.41 (m, 5H), 4.92 (s, 2H), 1.42 (s, 6H).

^{13}C NMR (100 MHz, $CDCl_3$) δ 173.7, 172.6, 133.7, 128.9, 128.6, 128.5, 49.8, 41.9, 21.1.

HRMS (ESI) *m/z*: [M+H$^+$] calculated for $C_{12}H_{13}NO_3$: 220.0968; found: 220.0967.

^{1}H NMR of **2b** in $CDCl_3$:

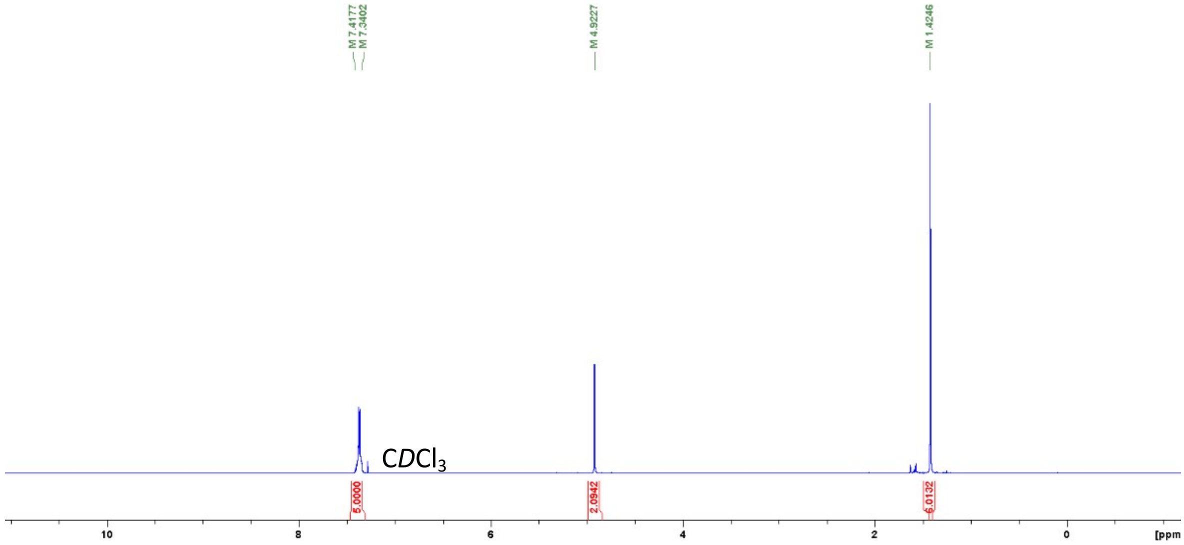

^{13}C NMR of **2b** in $CDCl_3$:

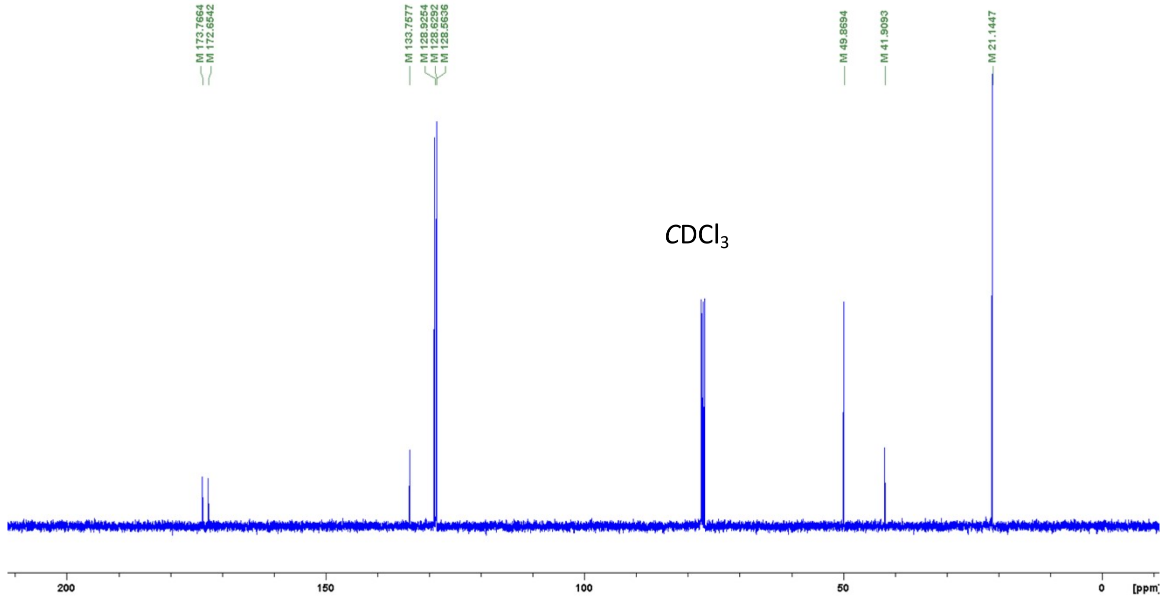

2-[(2-Fluorophenyl)methyl]-4,4-dimethyl-1,2-oxazolidine-3,5-dione **2c**

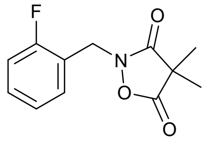

Prepared from **hydroxylamine 5c** using **GP3**. Colourless oil (121 mg, 51%).

^{1}H NMR (400 MHz, $CDCl_3$) δ 7.32-7.38 (m, 2H), 7.16 (td, *J* = 1.0, 7.6 Hz, 1H), 7.10 (td, *J* = 0.8 and 8.6 Hz, 1H), 5.01 (s, 2H), 1.44 (s, 6H).

^{13}C NMR (100 MHz, $CDCl_3$) δ 173.6, 172.4, 160.9 (d, *J* = 248.8 Hz), 130.6 (d, *J* = 5.2 Hz), 130.5 (d, *J* = 3.2 Hz), 124.5 (d, *J* = 3.9 Hz), 120.7 (d, *J* = 14.3 Hz), 115.8 (d, *J* = 21.2 Hz), 43.4 (d, *J* = 4.1 Hz), 41.8, 21.2.

^{19}F NMR (400 MHz, $CDCl_3$) δ -117.9.

HRMS (ESI) *m/z*: [M+H^{+}] calculated for $C_{12}H_{12}FNO_3$: 238.0874; found: 238.0872.

Analytical data are consistent with those previously reported.[22]

^{1}H NMR of **2c** in $CDCl_3$:

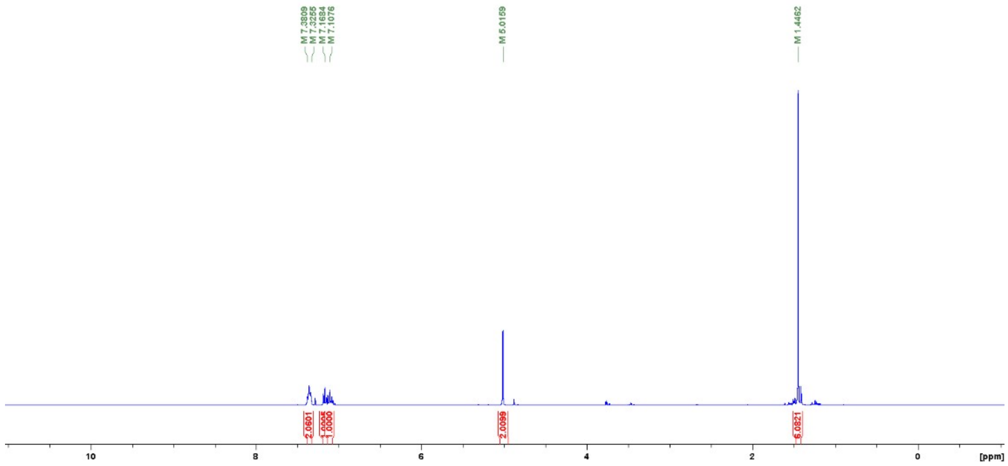

^{13}C NMR of **2c** in $CDCl_3$:

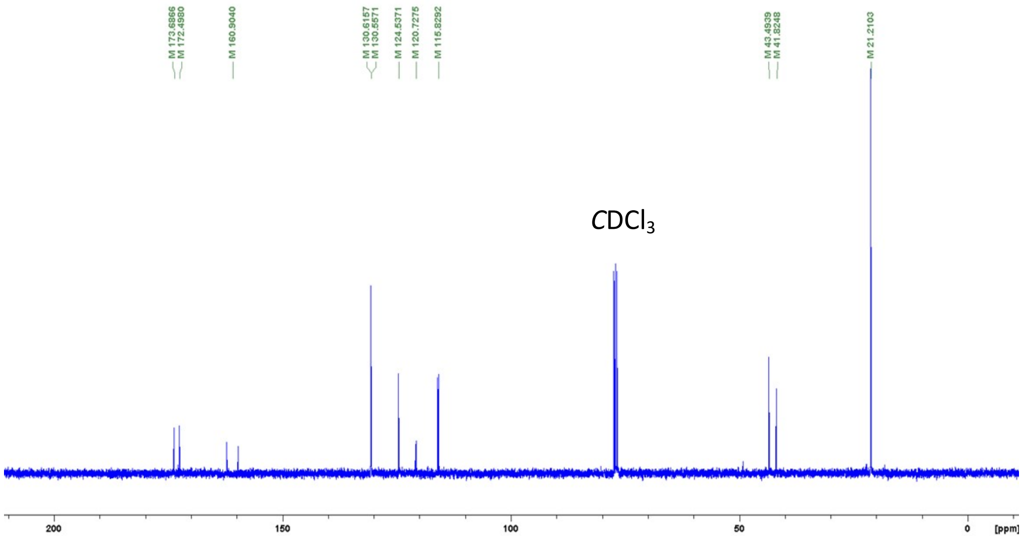

^{13}C DEPT-135 NMR of **2c** in $CDCl_3$:

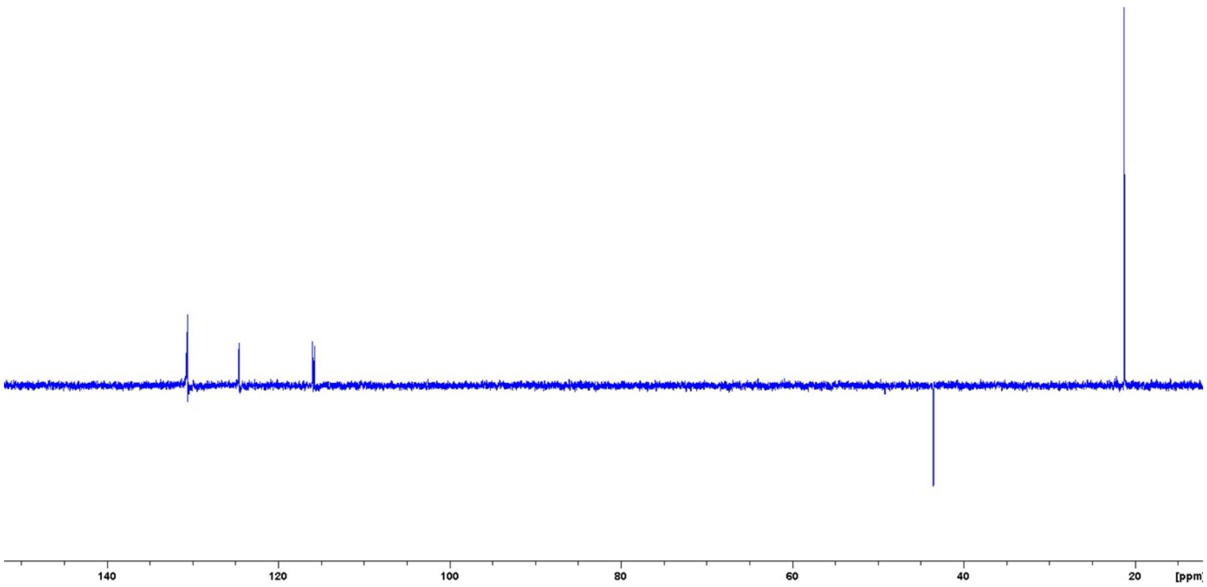

^{19}F NMR of **2c** in $CDCl_3$:

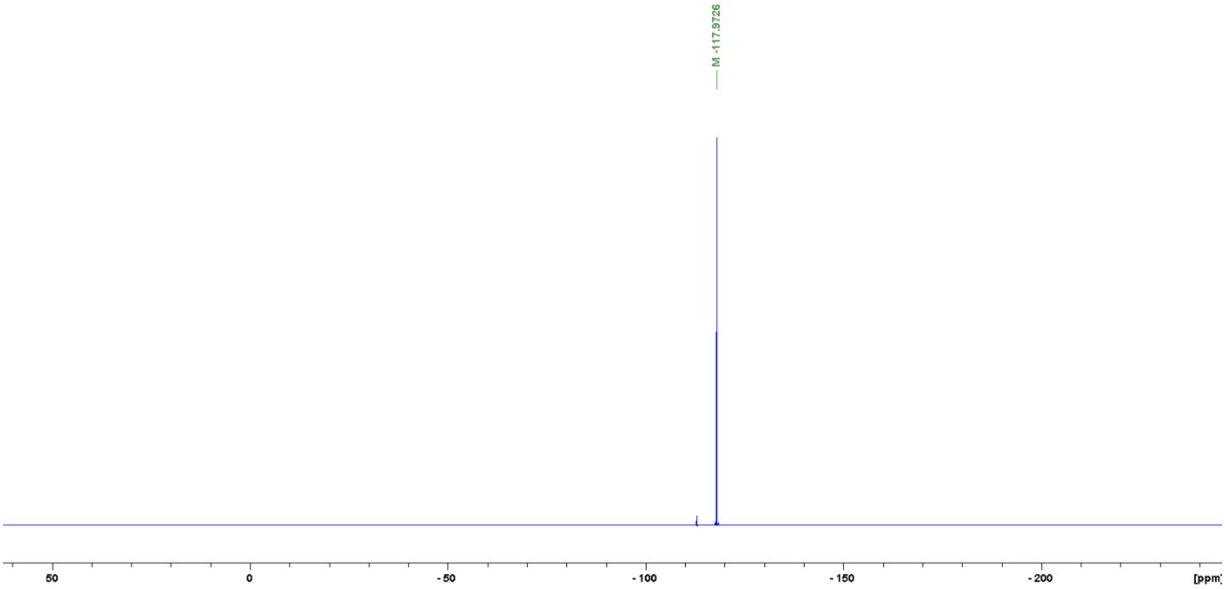

2-[(2-Bromophenyl)methyl]-4,4-dimethyl-1,2-oxazolidine-3,5-dione **2d**

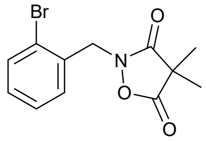

Prepared from **hydroxylamine 5d** using **GP3**. Colourless oil (194 mg, 65%).

^{1}H NMR (400 MHz, $CDCl_3$) δ 7.62 (dd, *J* = 1.0, 8.0 Hz, 1H), 7.33-7.39 (m, 2H), 7.24 (m, 1H), 5.08 (s, 2H), 1.49 (s, 6H).

^{13}C NMR (100 MHz, $CDCl_3$) δ 173.6, 172.0, 133.2, 132.9, 130.13, 130.12, 127.8, 123.4, 49.6, 41.8, 21.4.

HRMS (ESI) *m/z*: [M+H$^+$] calculated for $C_{12}H_{12}BrNO_3$: 298.0073; found: 298.0076.

Analytical data are consistent with those previously reported.[21]

^{1}H NMR of **2d** in $CDCl_3$:

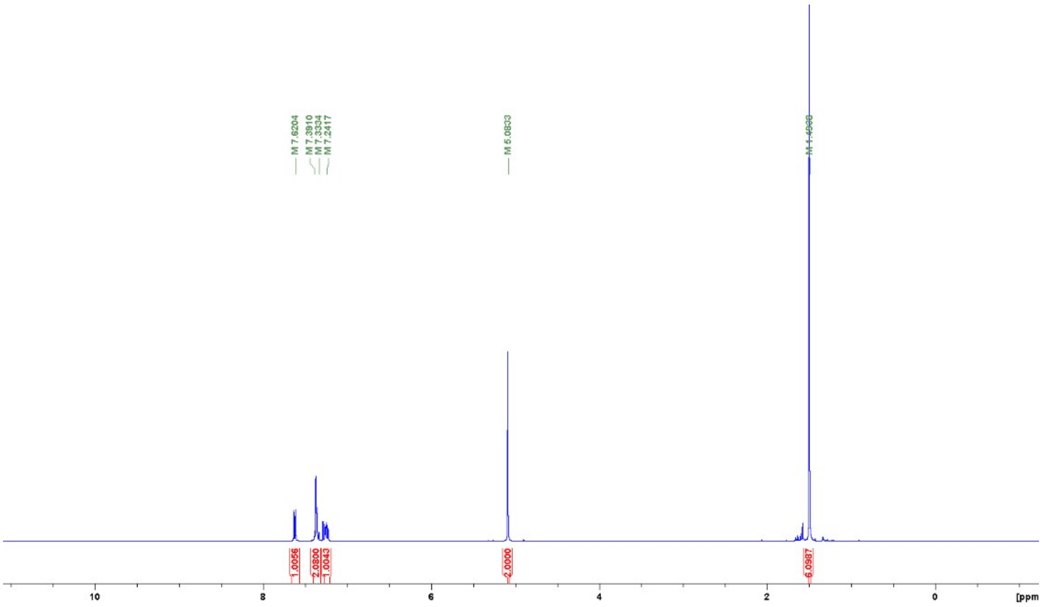

^{13}C NMR of **2d** in $CDCl_3$:

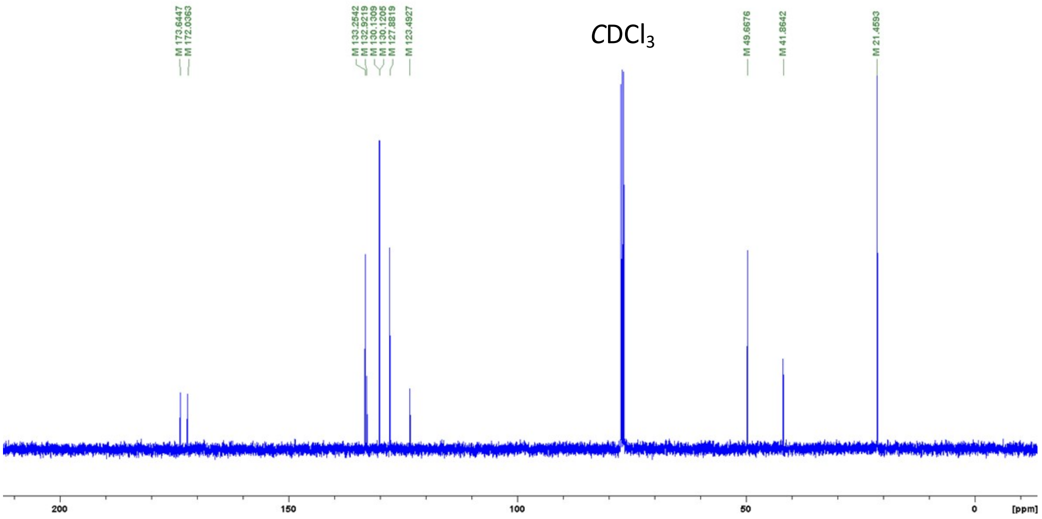

M 173.6447
M 172.0363
M 133.2542
M 132.9219
M 130.1309
M 130.1205
M 127.8819
M 123.4927
CDCl3
M 49.6676
M 41.8642
M 21.4593
200
150
100
50
0
[ppm]

2-[(4-Chlorophenyl)methyl]-4,4-dimethyl-1,2-oxazolidine-3,5-dione **2e**

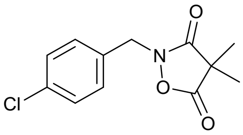

Prepared from **hydroxylamine 5e** using **GP3**. Colourless oil (188 mg, 74%).

¹H NMR (400 MHz, $CDCl_3$) δ 7.37 (d, *J* = 8.5 Hz, 2H), 7.30 (d, *J* = 8.5 Hz, 2H), 4.88 (s, 2H), 1.42 (s, 6H).

¹³C NMR (100 MHz, $CDCl_3$) δ 173.5, 172.7, 134.7, 132.2, 130.0, 129.1, 49.1, 41.9, 21.1.

HRMS (ESI) *m/z*: [M+H⁺] calculated for $C_{12}H_{12}ClNO_3$: 254.0578; found: 254.0579.

¹H NMR of **2e** in $CDCl_3$:

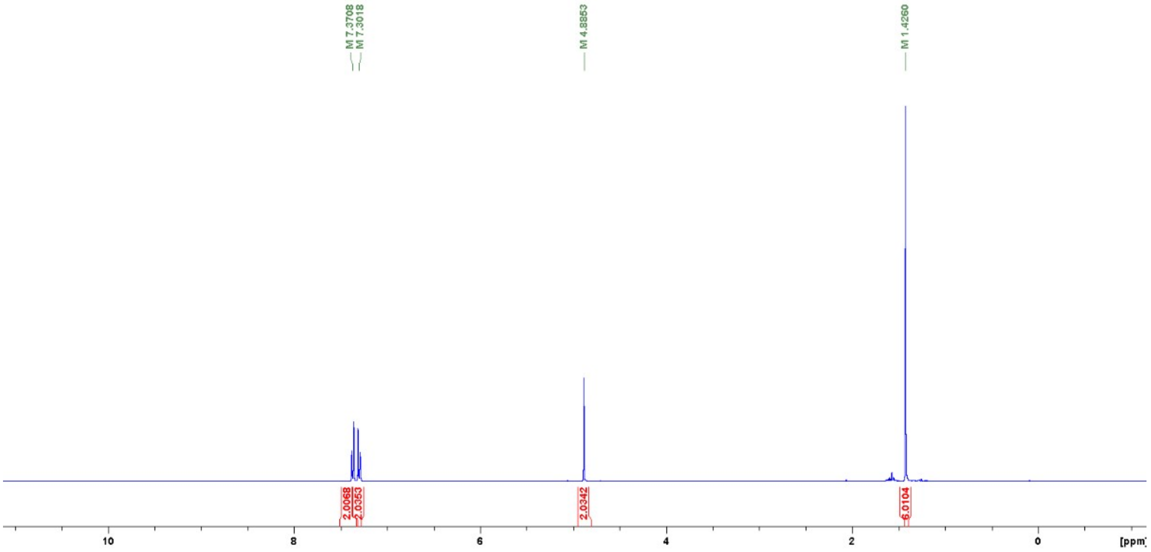

¹³C NMR of **2e** in $CDCl_3$:

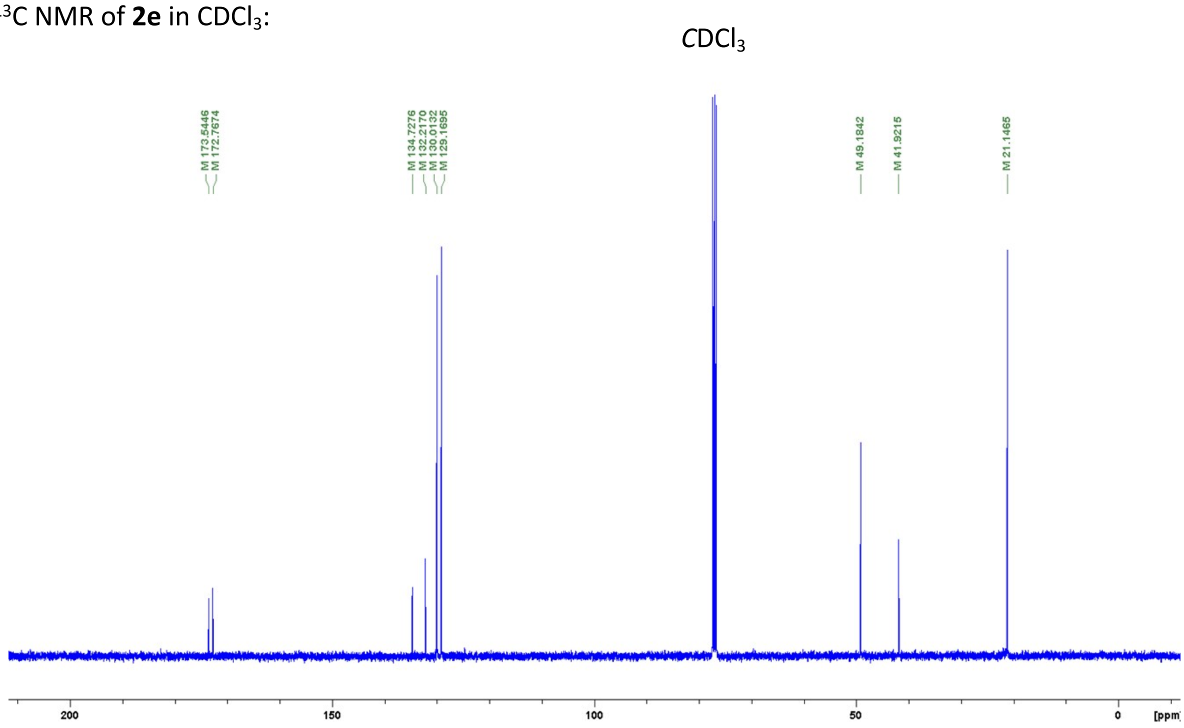

2-[(4-Fluorophenyl)methyl]-4,4-dimethyl-1,2-oxazolidine-3,5-dione **2f**

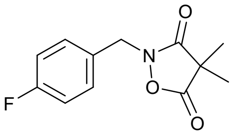

Prepared from **hydroxylamine 5f** using **GP3**. Pale yellow oil (180 mg, 76%).

^{1}H NMR (400 MHz, $CDCl_3$) δ 7.33 (m, 2H), 7.06 (m, 2H), 4.87 (s, 2H), 1.40 (s, 6H).

^{13}C NMR (100 MHz, $CDCl_3$) δ 173.6, 172.7, 162.7 (d, *J* = 147.8 Hz), 130.5 (d, *J* = 8.3 Hz), 129.6 (d, *J* = 3.5 Hz), 115.8 (d, *J* = 21.7 Hz), 49.1, 41.9, 21.1.

^{19}F NMR (400 MHz, $CDCl_3$) δ -112.9.

HRMS (ESI) *m/z*: [M+H^{+}] calculated for $C_{12}H_{12}FNO_3$: 238.0874; found: 238.0870.

^{1}H NMR of **2f** in $CDCl_3$:

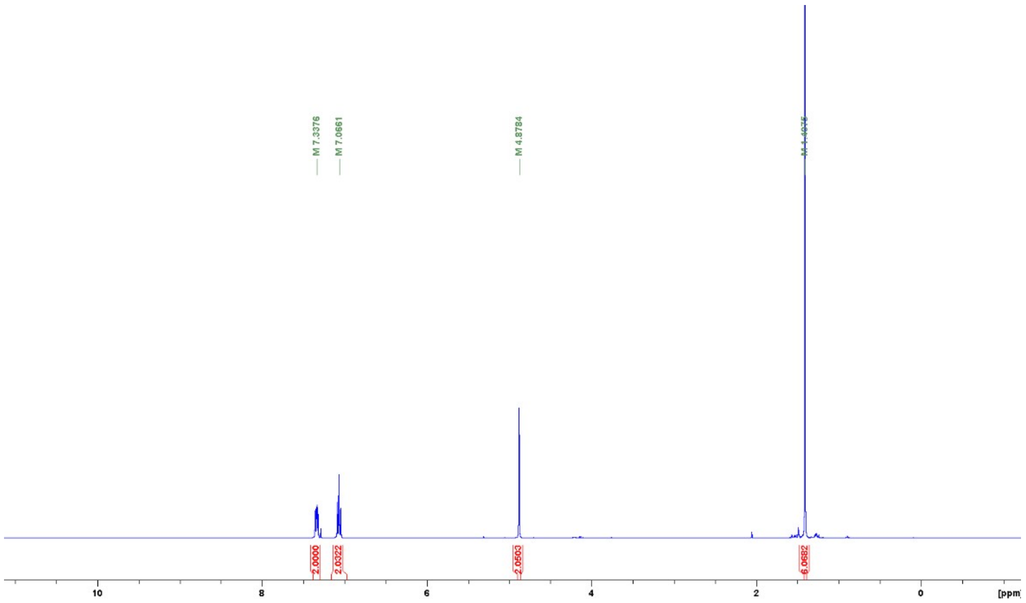

^{13}C NMR of **2f** in $CDCl_3$:

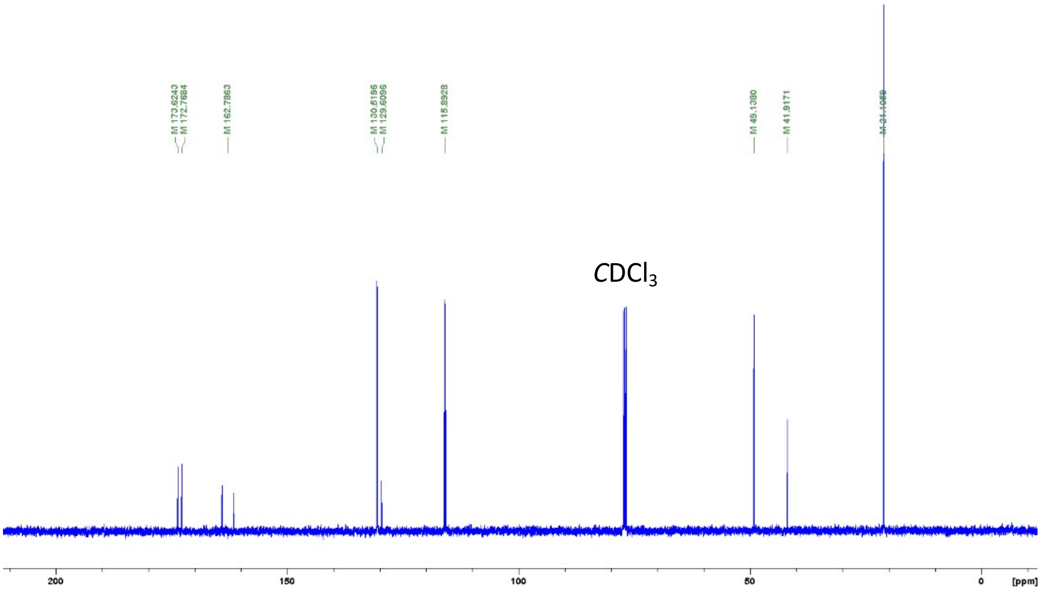

^{13}C DEPT-135 NMR of **2f** in $CDCl_3$:

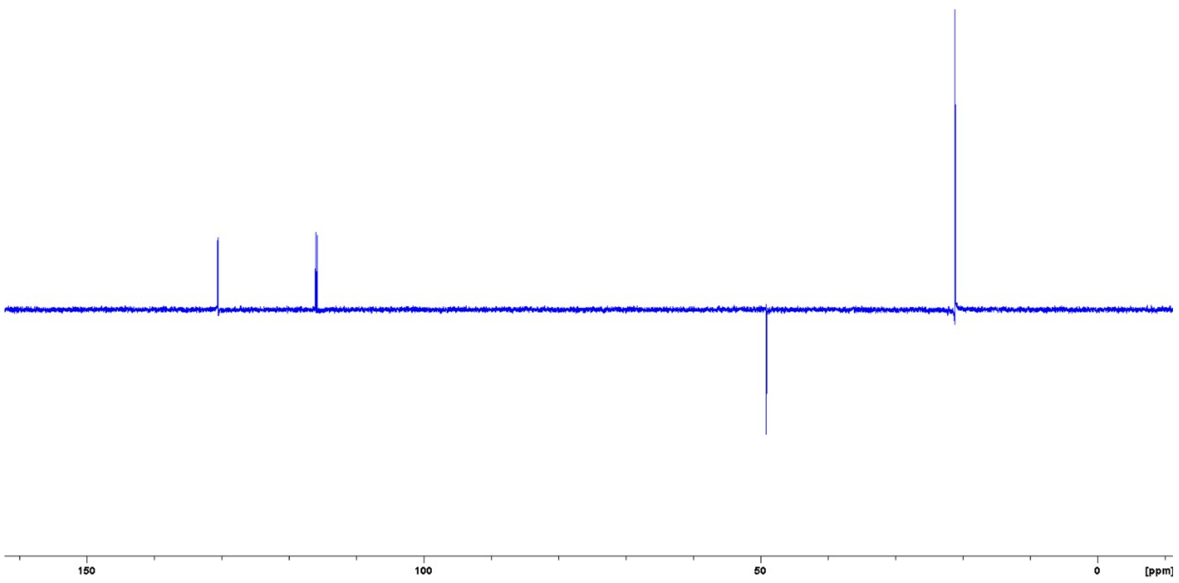

^{19}F NMR of **2f** in $CDCl_3$:

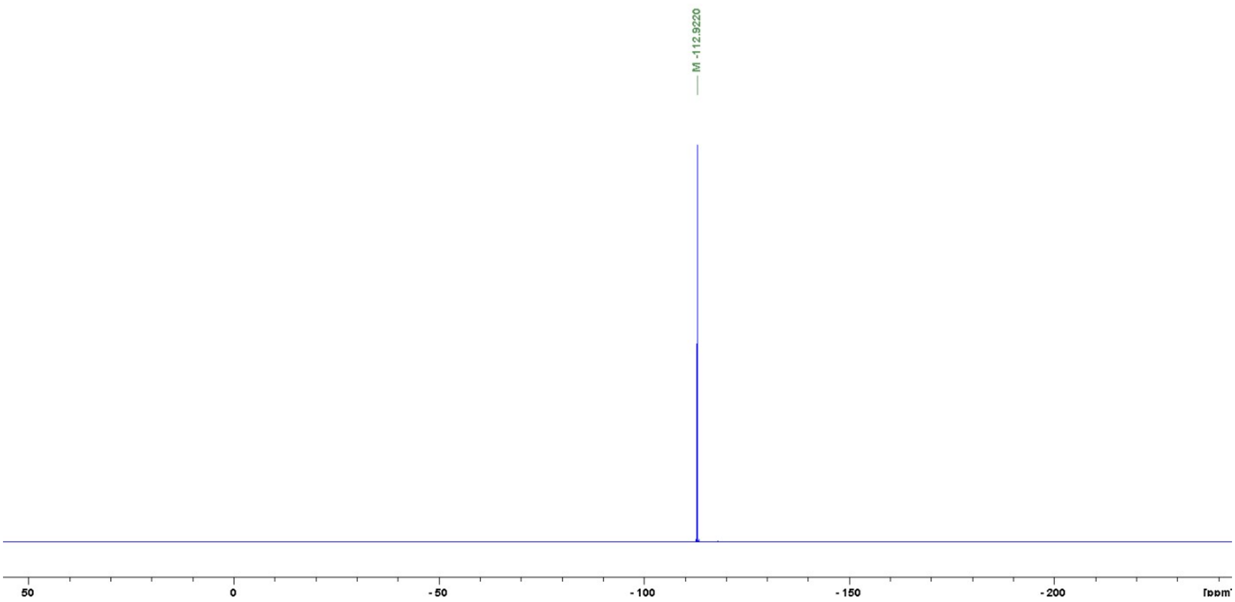

2-[(4-Bromophenyl)methyl]-4,4-dimethyl-1,2-oxazolidine-3,5-dione **2g**

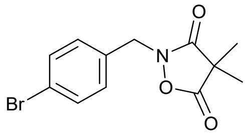

Prepared from **hydroxylamine 5g** using **GP3**. Colourless oil (164 mg, 55%).

^{1}H NMR (400 MHz, $CDCl_3$) δ 7.51 (d, *J* = 8.4 Hz, 2H), 7.23 (d, *J* = 8.4 Hz, 2H), 4.86 (s, 2H), 1.42 (s, 6H).

^{13}C NMR (100 MHz, $CDCl_3$) δ 173.5, 172.7, 132.7, 132.1, 130.2, 122.8, 49.2, 41.8, 21.1.

HRMS (ESI) *m/z*: [M+H$^+$] calculated for $C_{12}H_{12}BrNO_3$: 298.0073; found: 298.0071.

^{1}H NMR of **2g** in $CDCl_3$:

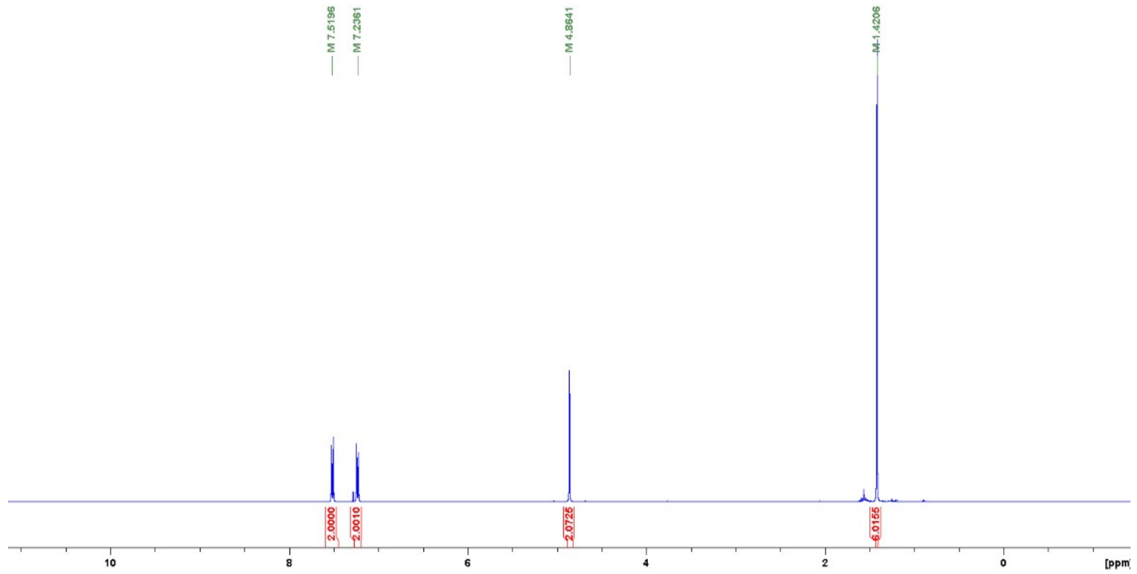

^{13}C NMR of **2g** in $CDCl_3$:

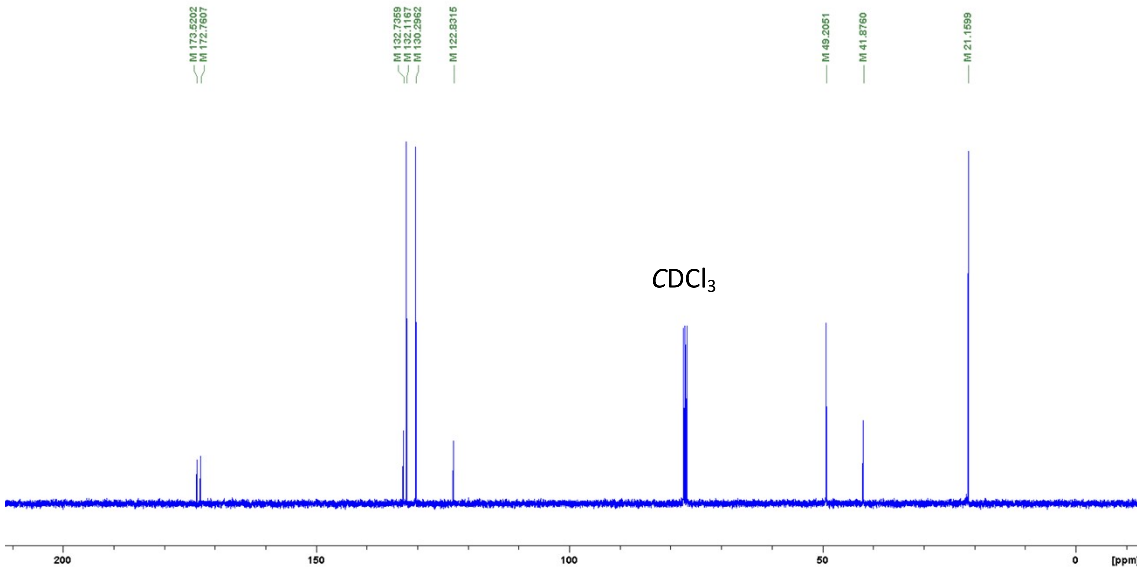

2-[(2-Methylphenyl)methyl]-4,4-dimethyl-1,2-oxazolidine-3,5-dione **2h**

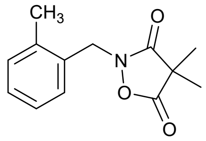

Prepared from **hydroxylamine 5h** using **GP3**. Colourless oil (198 mg, 85%).

^{1}H NMR (400 MHz, $CDCl_3$) δ 7.20-7.32 (m, 4H), 4.96 (s, 2H), 2.41 (s, 3H), 1.45 (s, 6H).

^{13}C NMR (100 MHz, $CDCl_3$) δ 173.7, 171.8, 136.7, 131.7, 130.7, 129.6, 128.8, 126.4, 47.4, 41.8, 21.2, 19.2.

HRMS (ESI) *m/z*: [M+H$^+$] calculated for $C_{13}H_{15}NO_3$: 234.1125; found: 234.1134.

^{1}H NMR of **2h** in $CDCl_3$:

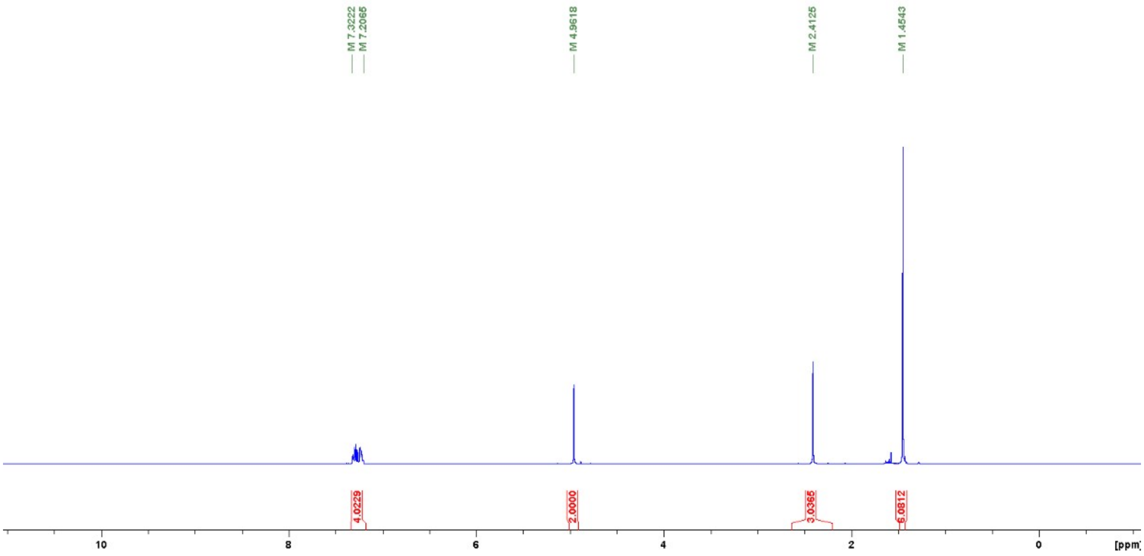

^{13}C NMR of **2h** in $CDCl_3$:

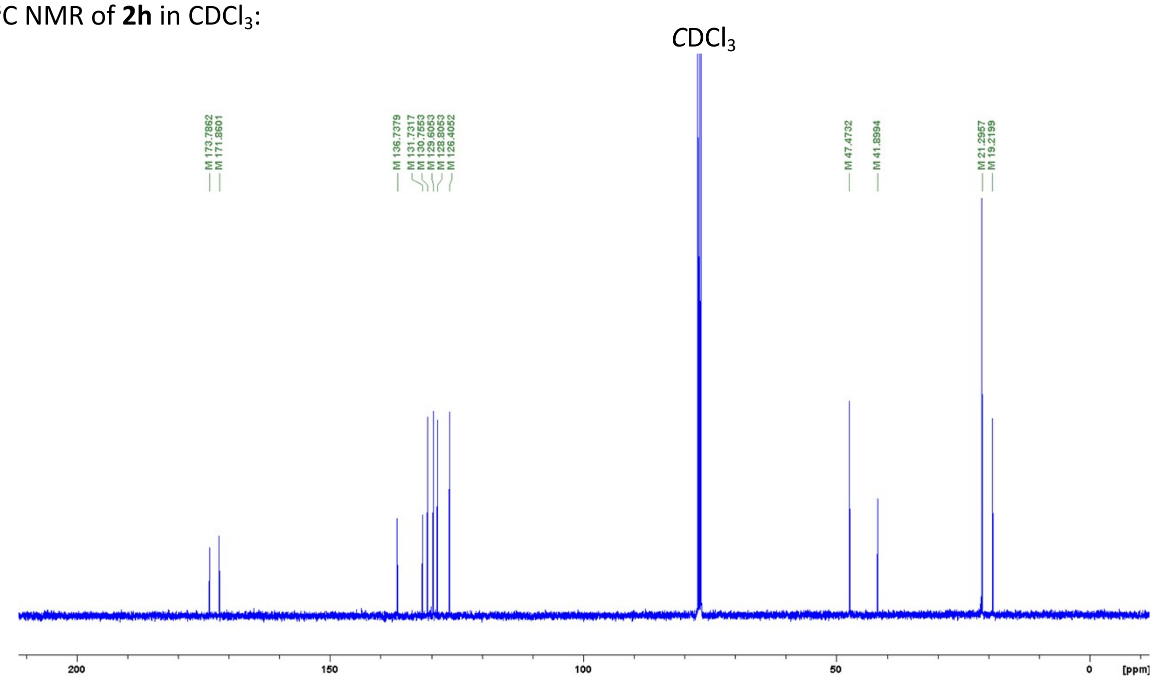

2-[(4-tert-Butylphenyl)methyl]-4,4-dimethyl-1,2-oxazolidine-3,5-dione **2i**

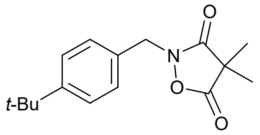

Prepared from **hydroxylamine 5i** using **GP3**. Colourless oil (193 mg, 70%).

^{1}H NMR (400 MHz, $CDCl_3$) δ 7.40 (d, J = 8.4 Hz, 2H), 7.28 (d, J = 8.4 Hz, 2H), 4.89 (s, 2H), 1.43 (s, 6H), 1.34 (s, 9H).

^{13}C NMR (100 MHz, $CDCl_3$) δ 173.8, 172.5, 151.6, 130.7, 128.3, 125.8, 49.5, 41.9, 34.6, 31.3, 21.1.

HRMS (ESI) *m/z*: [M+H$^+$] calculated for $C_{16}H_{21}NO_3$: 276.1594; found: 276.1604.

^{1}H NMR of **2i** in $CDCl_3$:

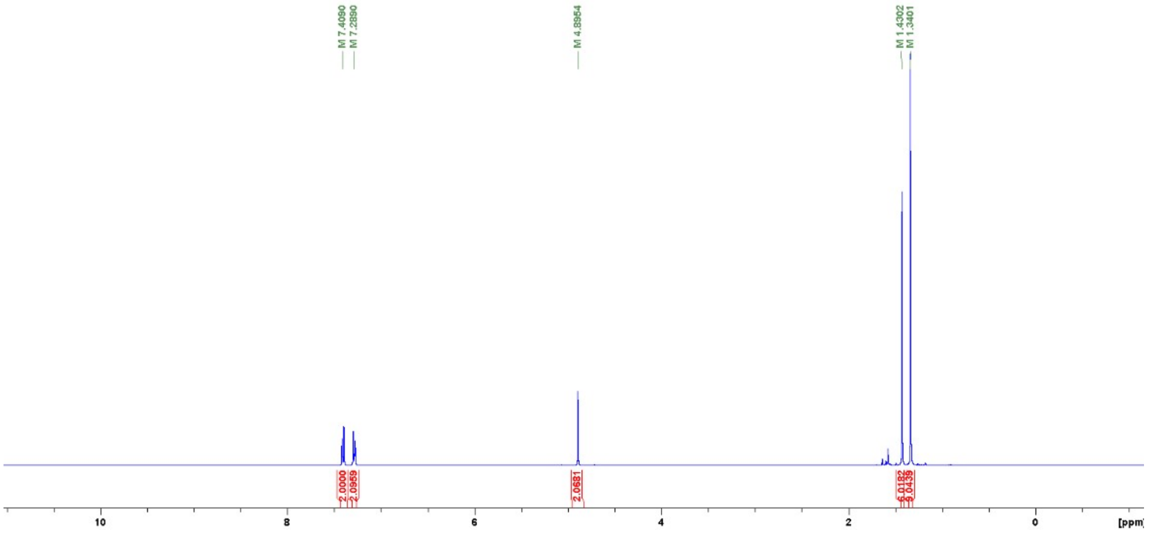

^{13}C NMR of **2i** in $CDCl_3$:

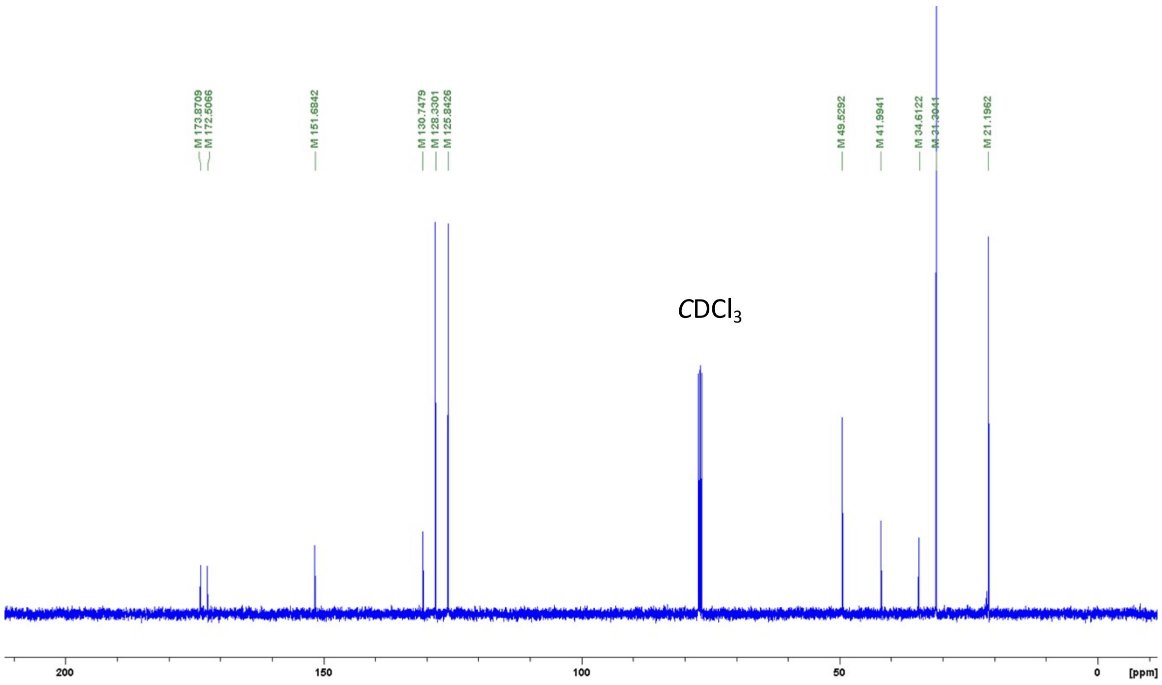

Potassium 2-[benzyl(hydroxy)carbamoyl]-2,2-dimethylacetate **3b**

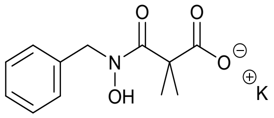

Prepared from **2b** using **GP4**. White solid (135 mg, 98%). **m.p.** 170-173 ^{o}C.

^{1}H NMR (400 MHz, CD_3OD) δ 7.25-7.37 (m, 5H), 4.81 (s, 2H), 1.40 (s, 6H).

^{13}C NMR (100 MHz, CD_3OD) δ 180.6, 175.9, 136.8, 127.9, 127.6, 126.7, 52.4, 50.8, 23.2.

HRMS (ESI) *m/z*: [M+H^{+}] calculated for $C_{12}H_{15}NO_4$: 238.1074; found: 238.1075.

^{1}H NMR of **3b** in CD_3OD:

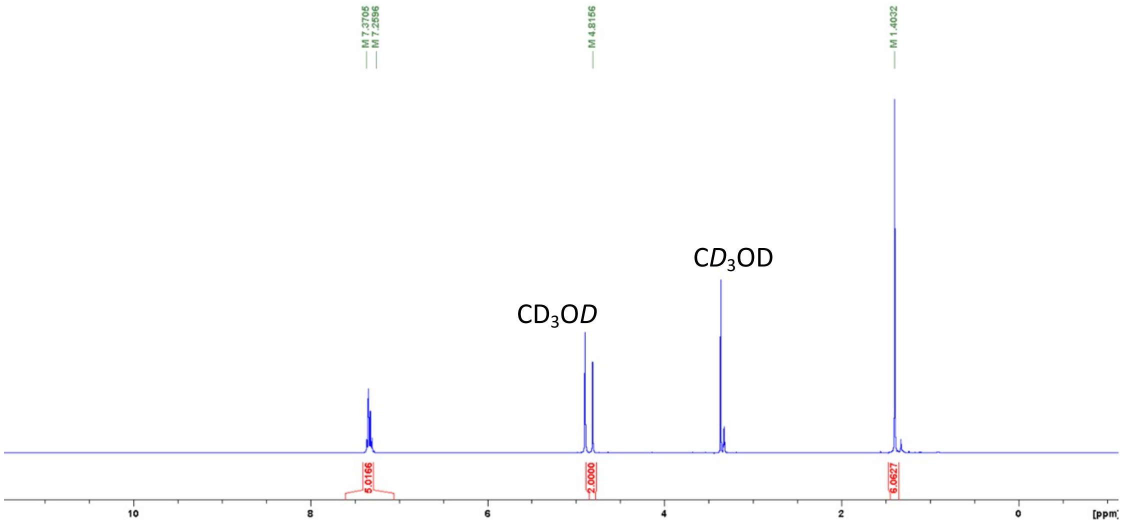

^{13}C NMR of **3b** in CD_3OD:

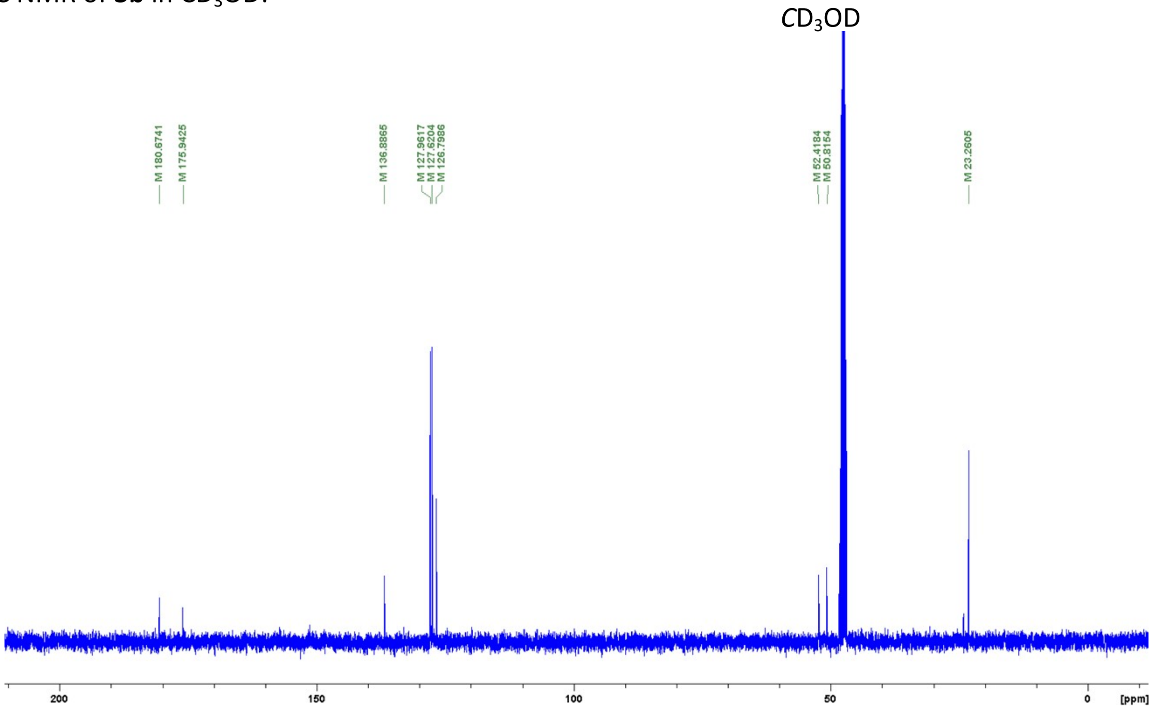

Potassium 2-{[(2-fluorophenyl)methyl](hydroxy)carbamoyl}-2,2-dimethylacetate **3c**

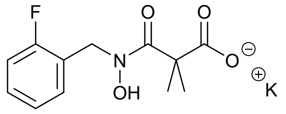

Prepared from **2c** using **GP4**. White solid (145 mg, 99%). **m.p.** 177-179 °C.

^{1}H NMR (400 MHz, CD_3OD) δ 7.47 (m, 1H), 7.28 (m, 1H), 7.15 (m, 1H), 7.06 (m, 1H), 4.89 (s, 2H), 1.40 (s, 6H).

^{13}C NMR (100 MHz, CD_3OD) δ 180.6, 176.1, 160.9 (d, *J* = 243.8 Hz), 129.7 (d, *J* = 4.3 Hz), 128.6 (d, *J* = 8.3 Hz), 123.8 (d, *J* = 3.6 Hz), 123.7 (d, *J* = 14.5 Hz), 114.5 (d, *J* = 20.9 Hz), 50.8, 46.1 (d, *J* = 5.3 Hz), 23.2.

^{19}F NMR (400 MHz, CD_3OD) δ -122.2.

HRMS (ESI) *m/z*: [M+H$^+$] calculated for $C_{12}H_{14}FNO_4$: 256.0979; found: 256.0989.

^{1}H NMR of **3c** in CD_3OD:

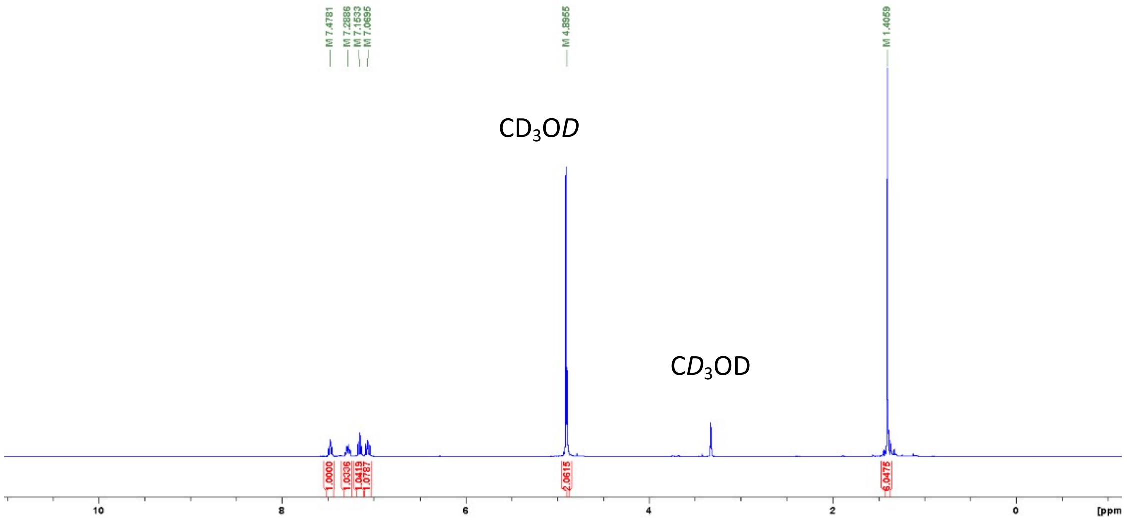

^{13}C NMR of **3c** in CD_3OD:

*C*D$_3$OD

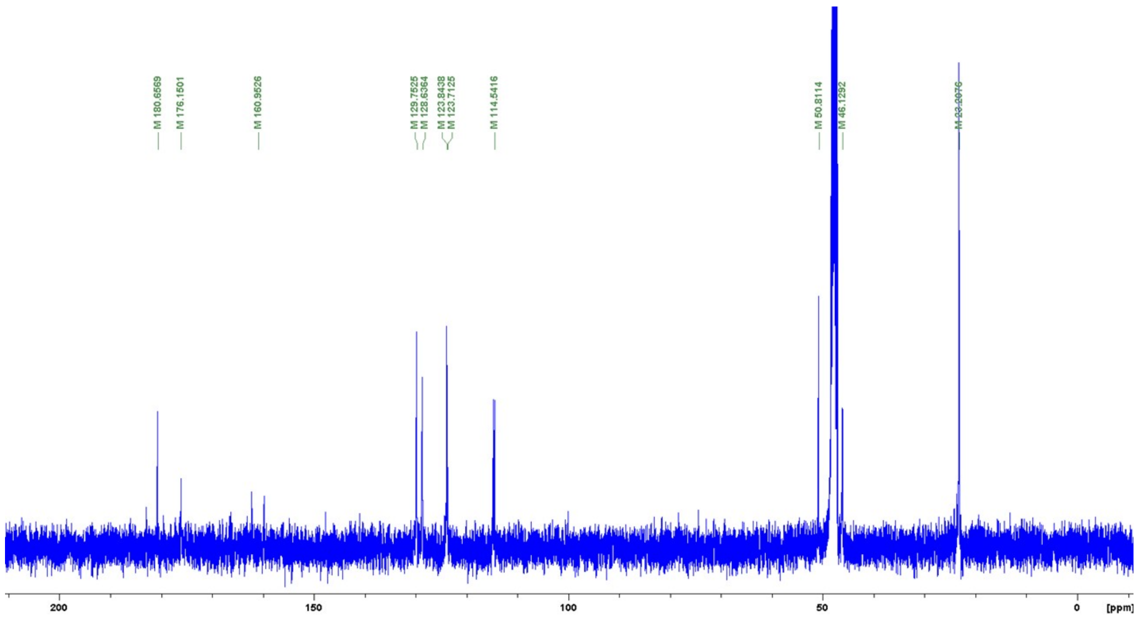

^{13}C DEPT-135 NMR of **3c** in CD_3OD:

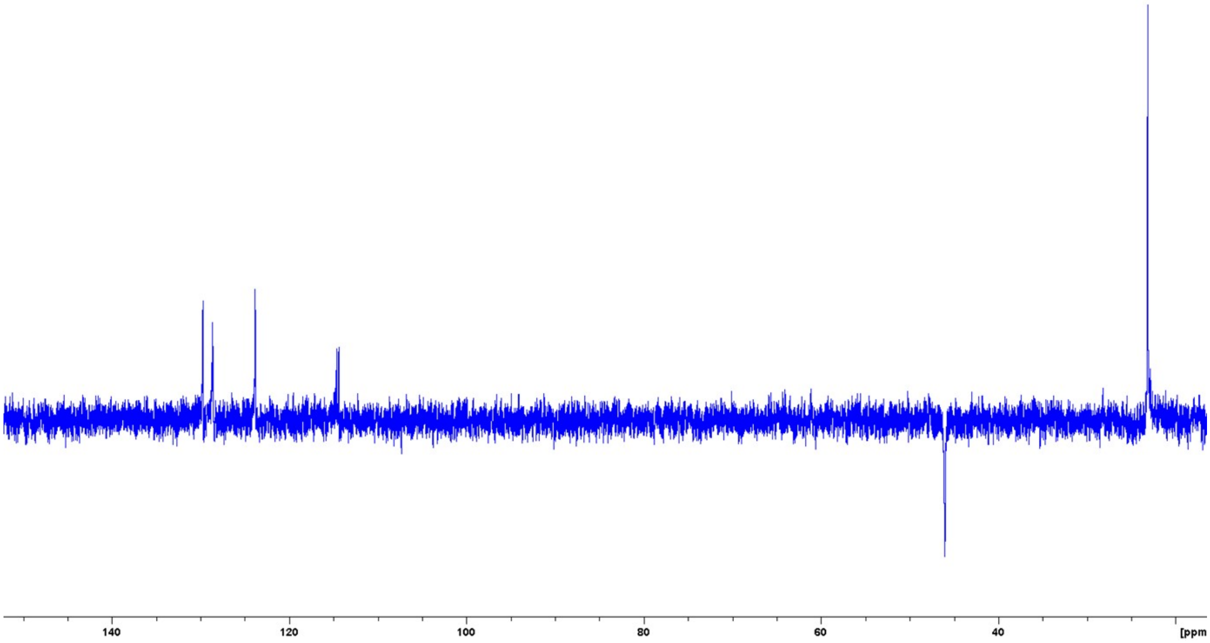

^{19}F NMR of **3c** in CD_3OD:

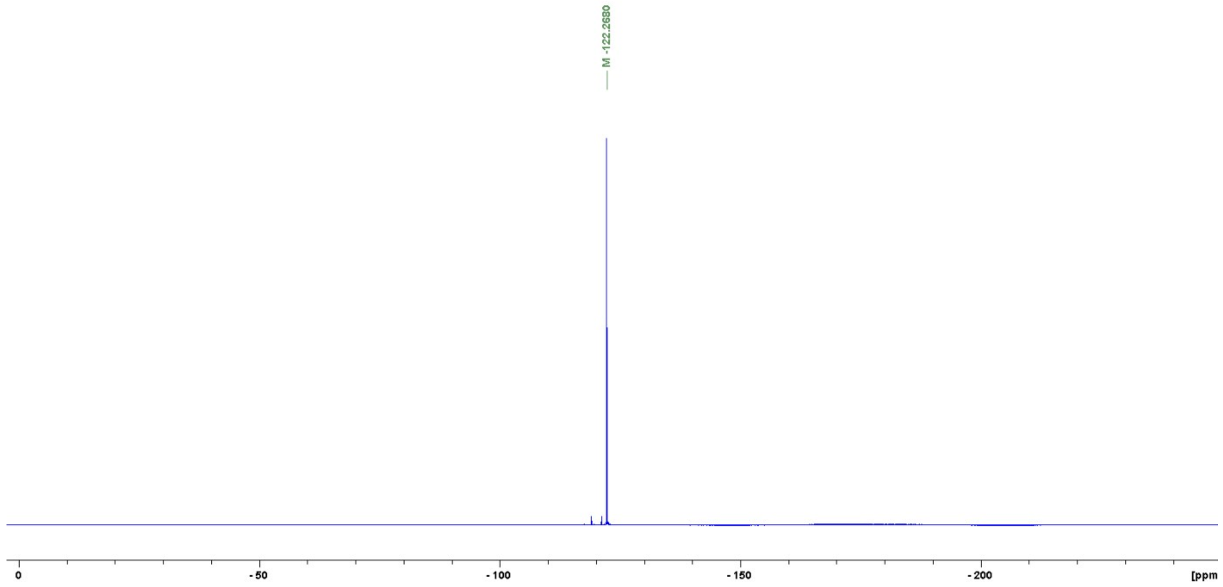
M -122.2680
0
-50
-100
-150
-200
[ppm

Potassium 2-{[(2-bromophenyl)methyl](hydroxy)carbamoyl}-2,2-dimethylacetate **3d**

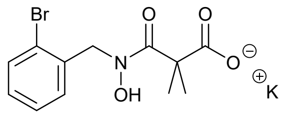

Prepared from **2d** using **GP4**. White solid (174 mg, 98%). **m.p.** 180-183 °C.

^{1}H NMR (400 MHz, CD_3OD) δ 7.56 (dd, *J* = 1.1, 7.8 Hz, 1H), 7.51 (dd, *J* = 1.5, 7.8 Hz, 1H), 7.35 (td, *J* = 1.1, 7.8, 7.8 Hz, 1H), 7.17 (td, *J* = 1.5, 7.8, 7.8 Hz, 1H), 4.90 (s, 2H), 1.44 (s, 6H).

^{13}C NMR (100 MHz, CD_3OD) δ 180.4, 176.3, 135.6, 132.1, 128.7, 128.3, 127.2, 122.3, 52.7, 50.9, 23.2.

HRMS (ESI) *m/z*: [M+H$^+$] calculated for $C_{12}H_{14}BrNO_4$: 316.0179; found: 316.0177.

^{1}H NMR of **3d** in CD_3OD:

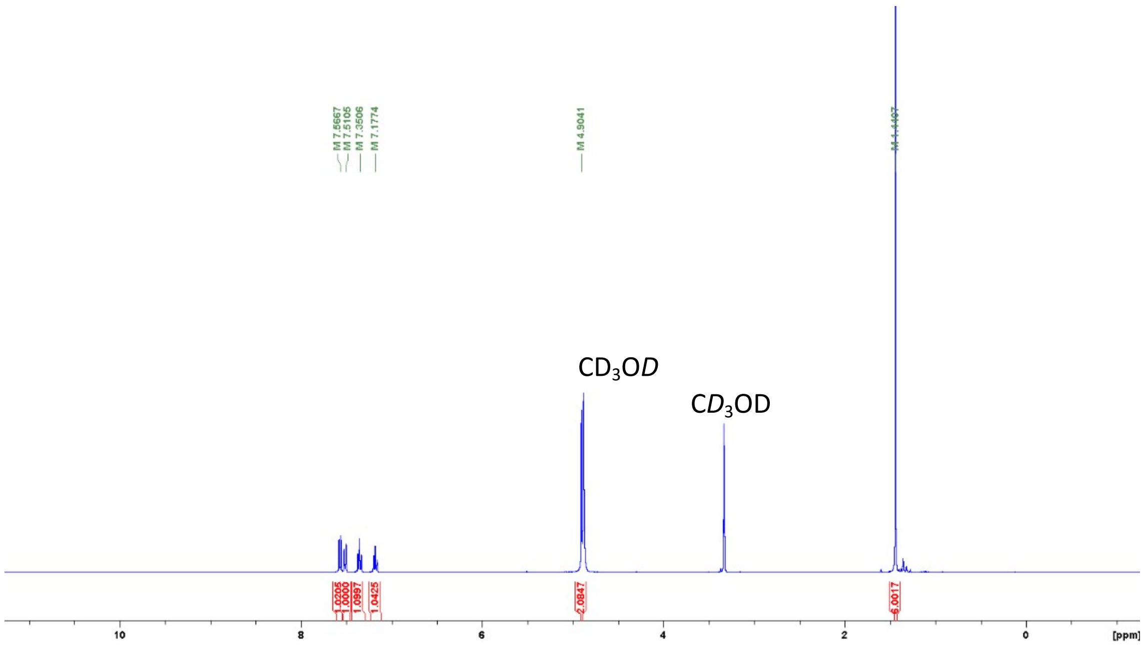

^{13}C NMR of **3d** in CD_3OD:

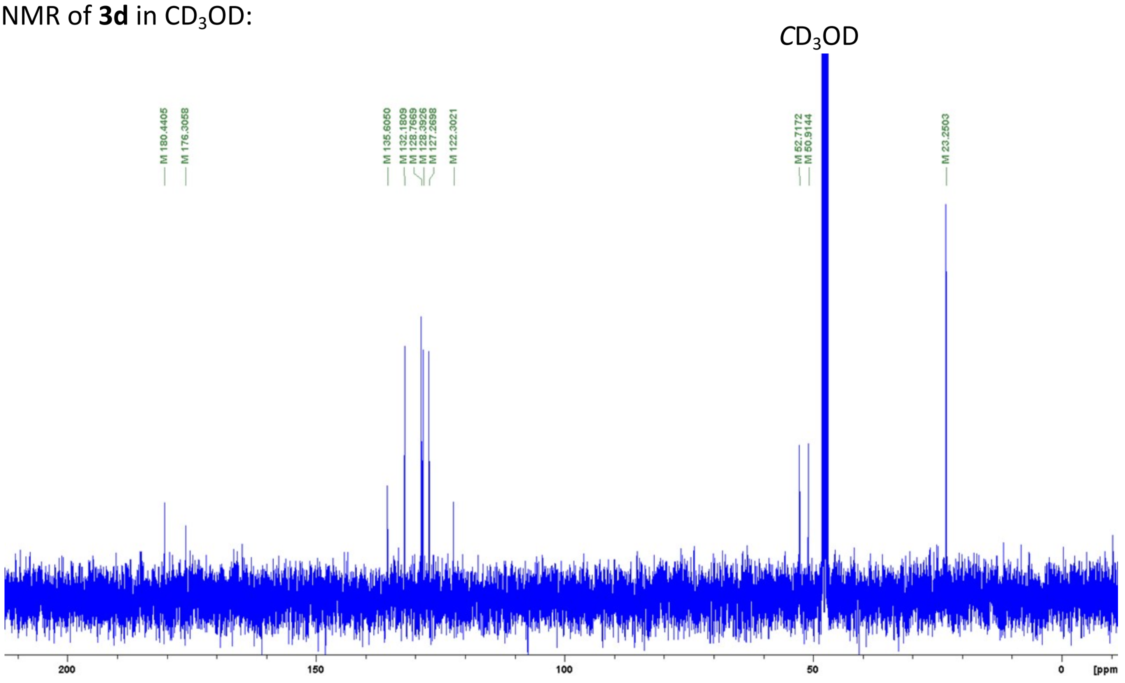

Potassium 2-{[(4-chlorophenyl)methyl](hydroxy)carbamoyl}-2,2-dimethylacetate **3e**

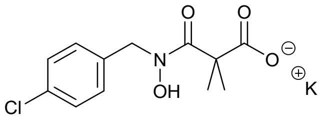

Prepared from **2e** using **GP4**. White solid (151 mg, 98%). **m.p.** 188-189 ^{o}C.

^{1}H NMR (400 MHz, CD_3OD) δ 7.32-7.35 (m, 4H), 4.79 (s, 2H), 1.39 (s, 6H).

^{13}C NMR (100 MHz, CD_3OD) δ 180.5, 176.2, 135.7, 132.6, 129.2, 128.0, 51.8, 50.8, 23.1.

HRMS (ESI) *m/z*: [M+H^{+}] calculated for $C_{12}H_{14}ClNO_4$: 272.0684; found: 272.0686.

^{1}H NMR of **3e** in CD_3OD:

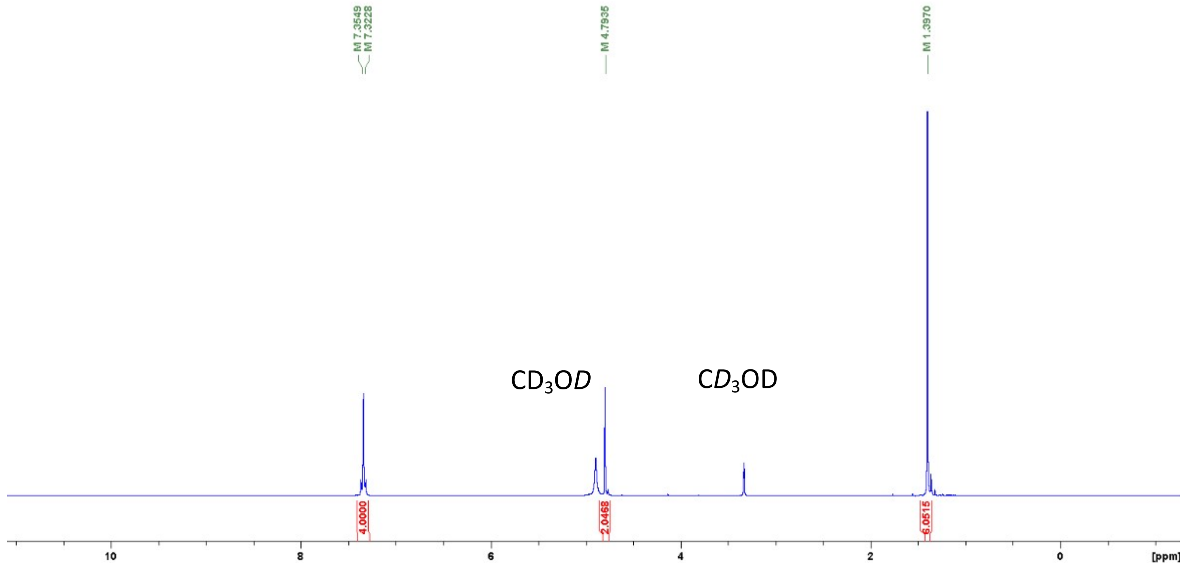

^{13}C NMR of **3e** in CD_3OD:

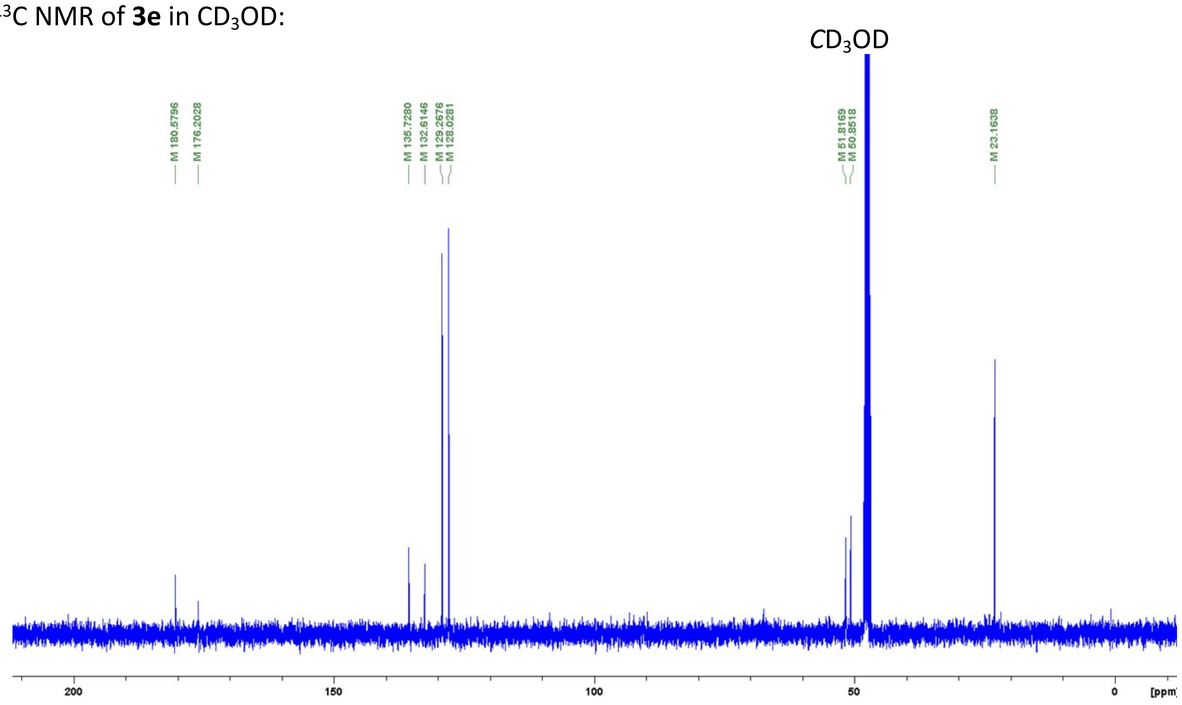

Potassium 2-{[(4-fluorophenyl)methyl](hydroxy)carbamoyl}-2,2-dimethylacetate **3f**

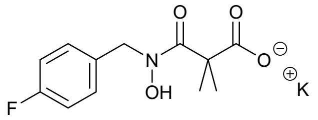

Prepared from **2f** using **GP4**. White solid (136 mg, 93%). **m.p.** 173-176 °C.

^{1}H NMR (400 MHz, CD_3OD) δ 7.37 (m, 2H), 7.04 (m, 2H), 4.79 (s, 2H), 1.39 (s, 6H).

^{13}C NMR (100 MHz, CD_3OD) δ 180.7, 175.7, 162.1 (d, *J* = 241.7 Hz), 132.9 (d, *J* = 3.3 Hz), 129.5 (d, *J* = 8.4 Hz), 114.5 (d, *J* = 21.7 Hz), 51.8, 50.6, 23.1.

^{19}F NMR (400 MHz, CD_3OD) δ -119.0.

HRMS (ESI) *m/z*: [M+H$^+$] calculated for $C_{12}H_{14}FNO_4$: 256.0980; found: 256.0989.

^{1}H NMR of **3f** in CD_3OD:

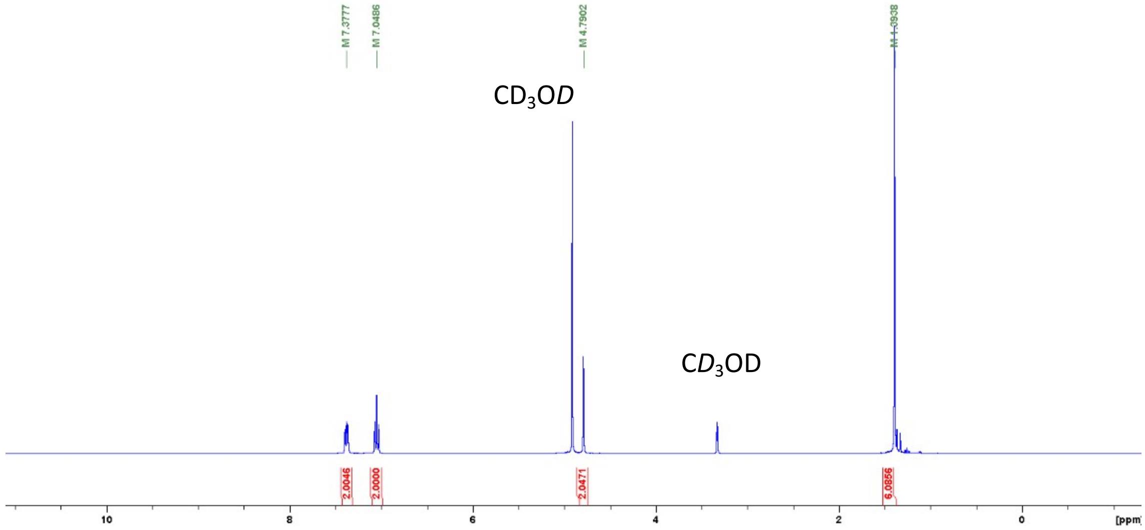

^{13}C NMR of **3f** in CD_3OD:

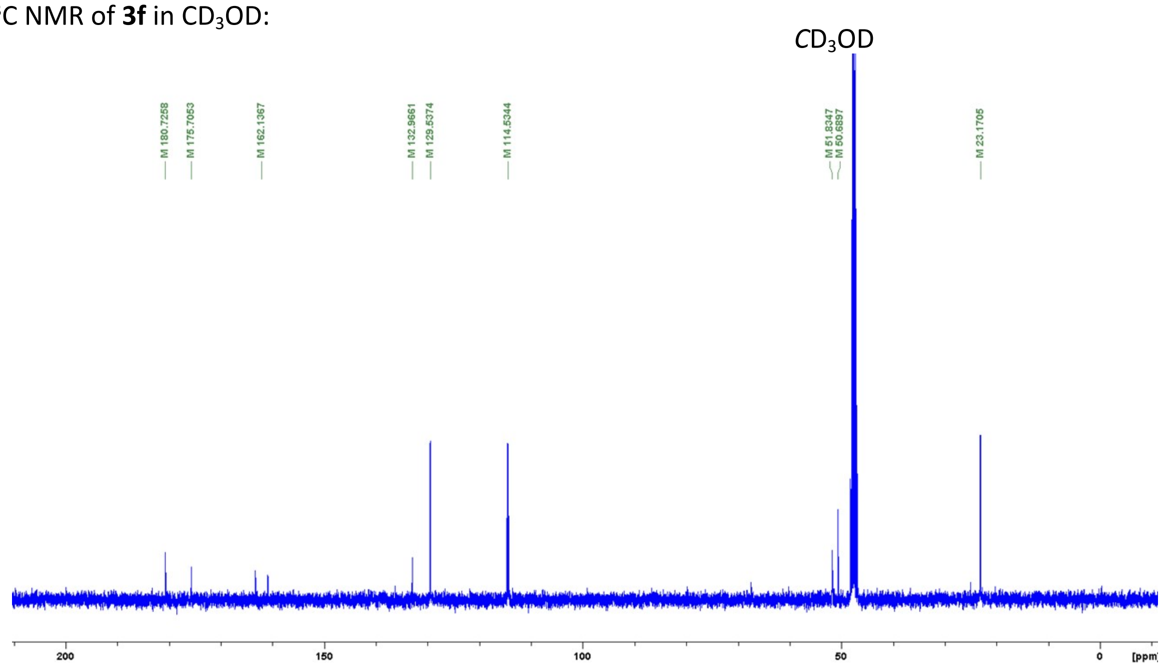

^{13}C DEPT-135 NMR of **3f** in CD_3OD:

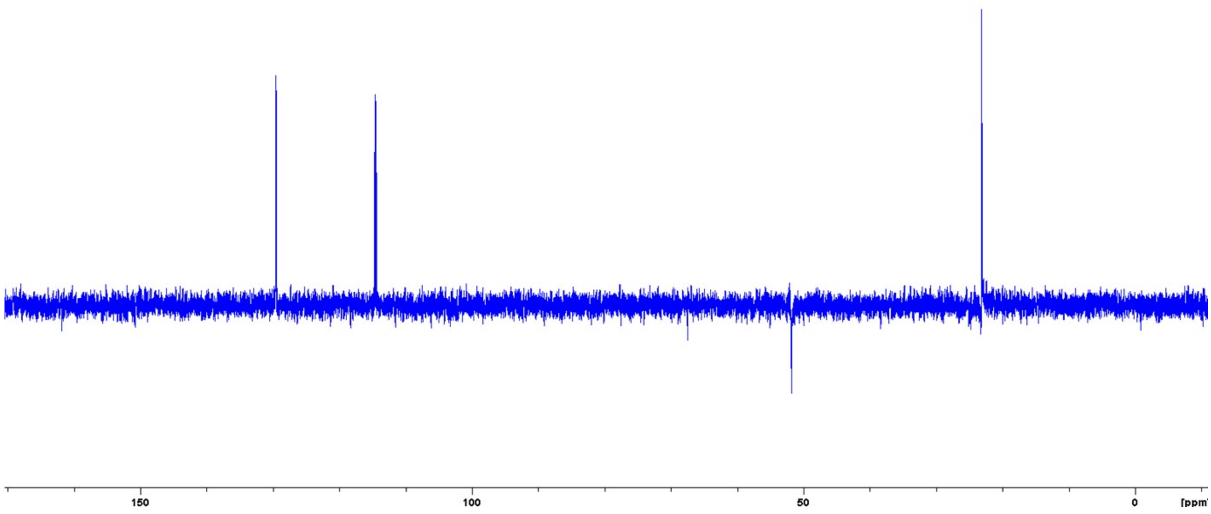

^{19}F NMR of **3f** in CD_3OD:

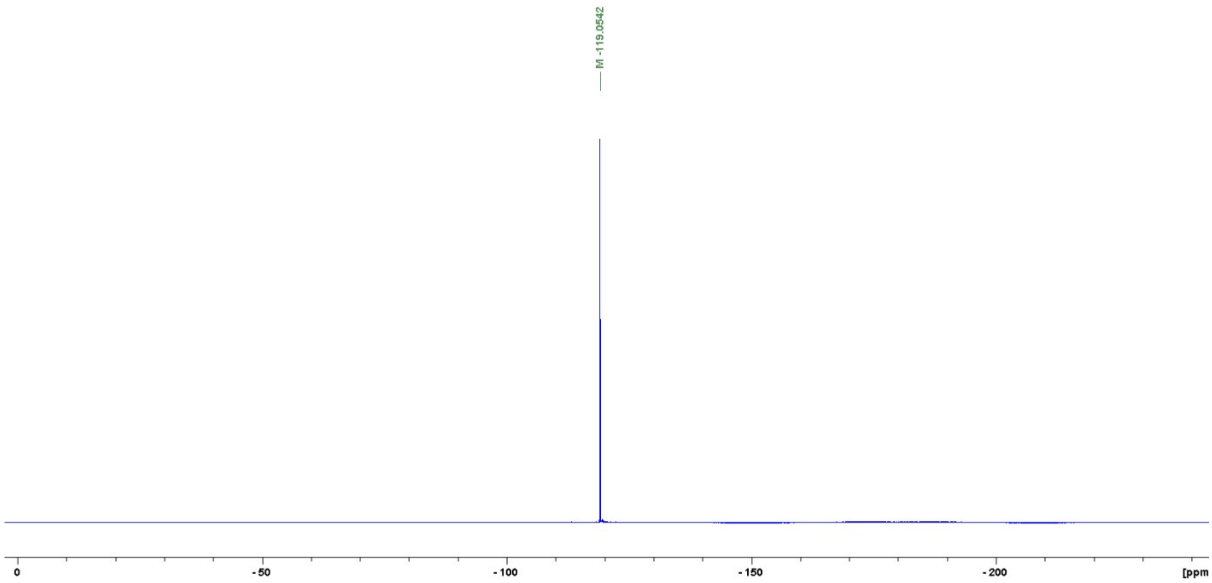

Potassium 2-{[(4-bromophenyl)methyl](hydroxy)carbamoyl}-2,2-dimethylacetate **3g**

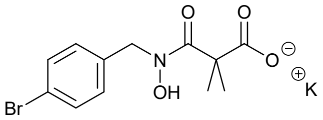

Prepared from **2g** using **GP4**. White solid (170 mg, 96%). **m.p.** 191-192 °C.

^{1}H NMR (400 MHz, CD_3OD) δ 7.47 (d, *J* = 8.6 Hz, 2H), 7.28 (d, *J* = 8.6 Hz, 2H), 4.78 (s, 2H), 1.39 (s, 6H).

^{13}C NMR (100 MHz, CD_3OD) δ 180.6, 176.2, 136.1, 131.1, 129.7, 120.7, 51.8, 50.7, 23.1.

HRMS (ESI) *m/z*: [M+H$^+$] calculated for $C_{12}H_{14}BrNO_4$: 316.0179; found: 316.0178.

^{1}H NMR of **3g** in CD_3OD:

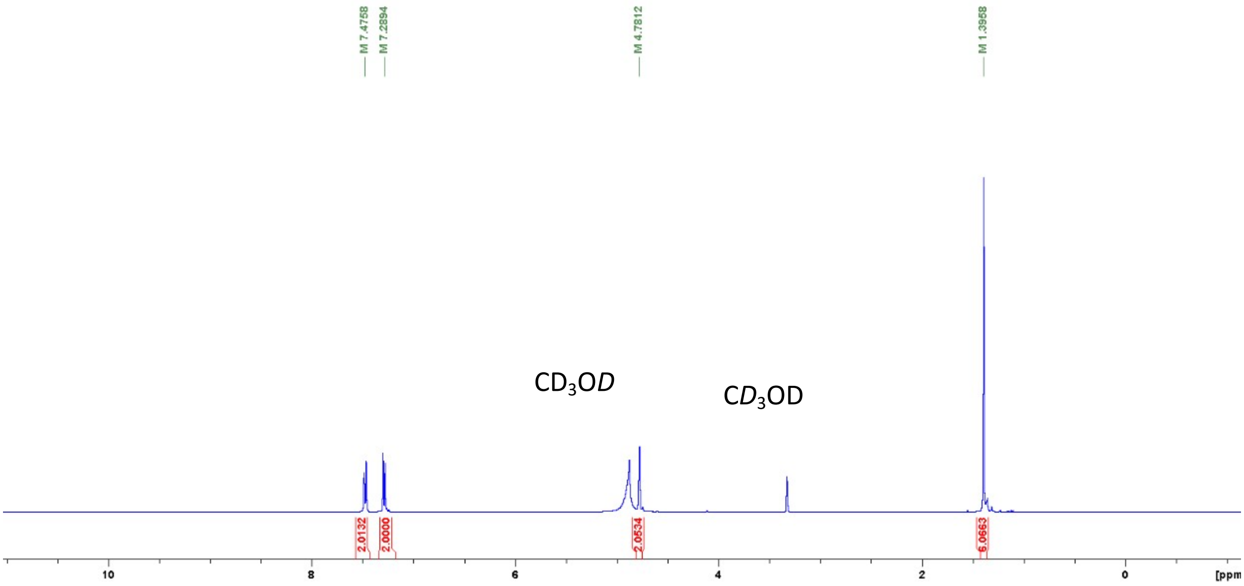

^{13}C NMR of **3g** in CD_3OD:

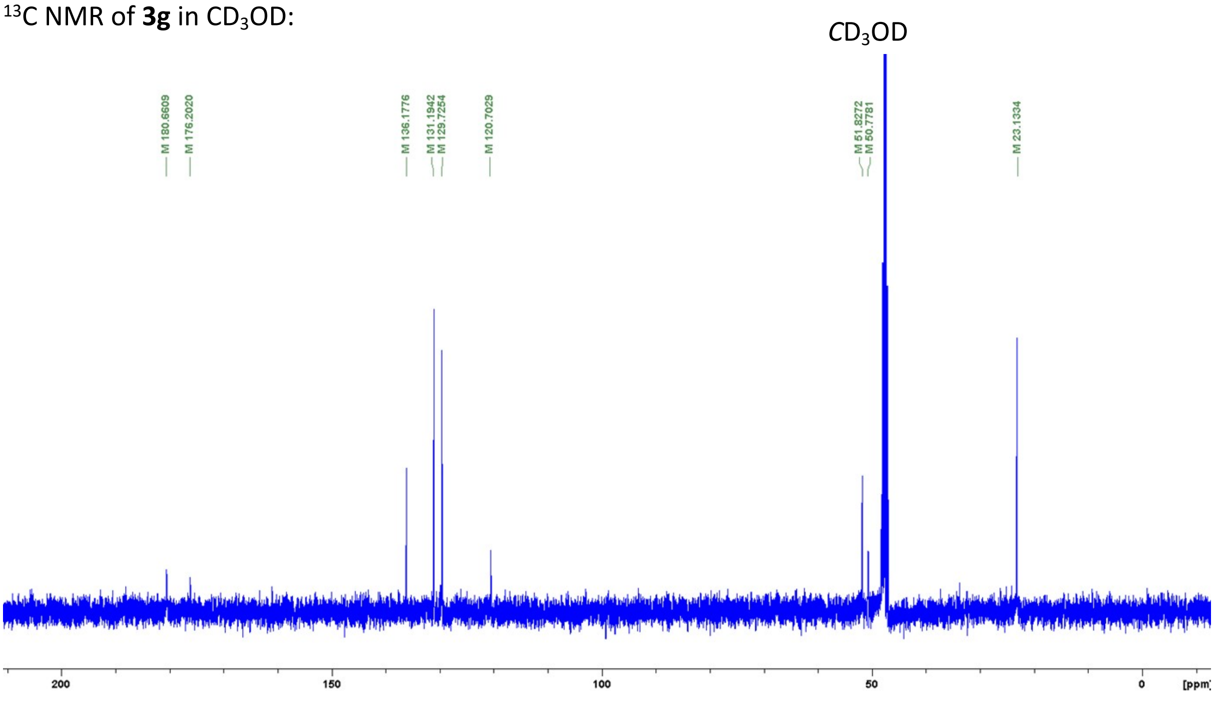

Potassium 2-{[(2-methylphenyl)methyl](hydroxy)carbamoyl}-2,2-dimethylacetate **3h**

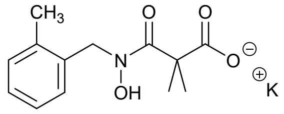

Prepared from **2h** using **GP4**. White solid (140 mg, 97%). **m.p.** 165-168 °C.

^{1}H NMR (400 MHz, CD_3OD) δ 7.16-7.33 (m, 4H), 4.83 (s, 2H), 2.32 (s, 3H), 1.40 (s, 6H).

^{13}C NMR (100 MHz, CD_3OD) δ 180.6, 175.9, 136.2, 134.3, 129.7, 128.4, 127.0, 125.5, 50.8, 50.3, 23.2, 17.8.

HRMS (ESI) *m/z*: [M+H$^+$] calculated for $C_{13}H_{17}NO_4$: 252.1230; found: 252.1227.

^{1}H NMR of **3h** in CD_3OD:

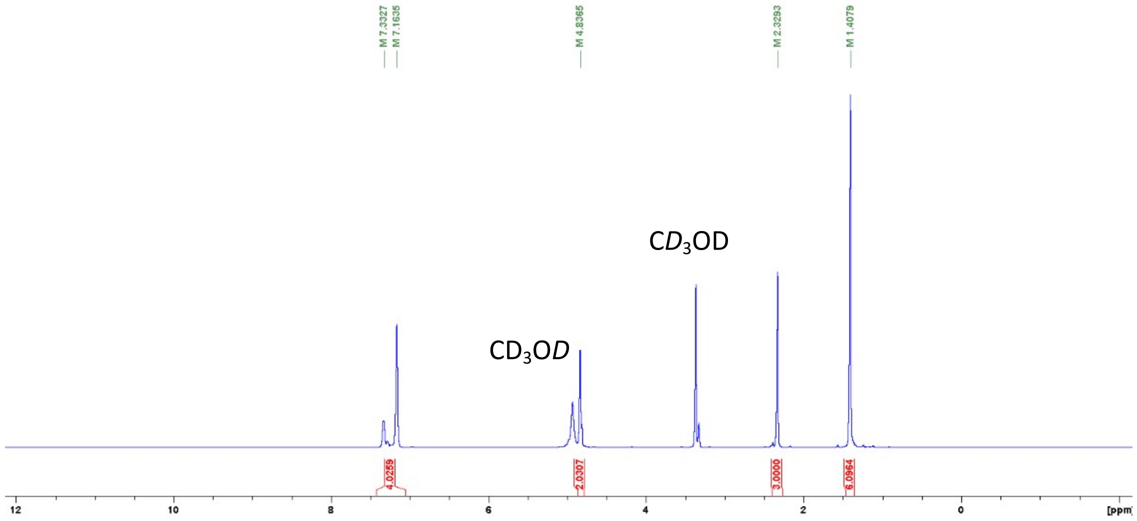

^{13}C NMR of **3h** in CD_3OD:

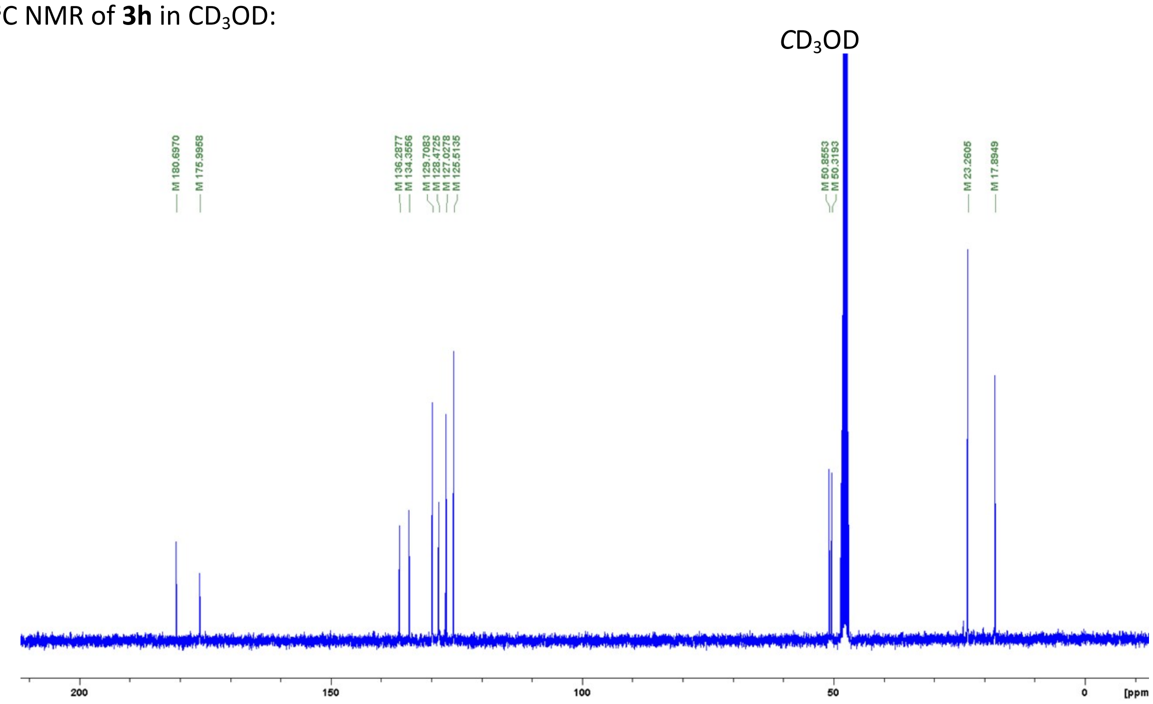

Potassium 2-{[(4-tert-butylphenyl)methyl](hydroxy)carbamoyl}-2,2-dimethylacetate **3i**

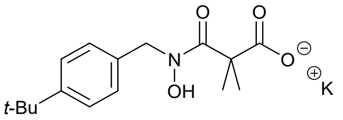

Prepared from **2i** using **GP4**. White solid (161 mg, 97%). **m.p.** 176-179 °C.

^{1}H NMR (400 MHz, CD_3OD) δ 7.37 (d, *J* = 8.4 Hz, 2H), 7.27 (d, *J* = 8.4 Hz, 2H), 4.78 (s, 2H), 1.40 (s, 6H), 1.32 (s, 9H).

^{13}C NMR (100 MHz, CD_3OD) δ 180.6, 175.9, 149.8, 133.8, 127.5, 124.8, 52.0, 50.9, 33.9, 30.4, 23.2.

HRMS (ESI) *m/z*: [M+H$^+$] calculated for $C_{16}H_{23}NO_4$: 294.1700; found: 294.1702.

^{1}H NMR of **3i** in CD_3OD:

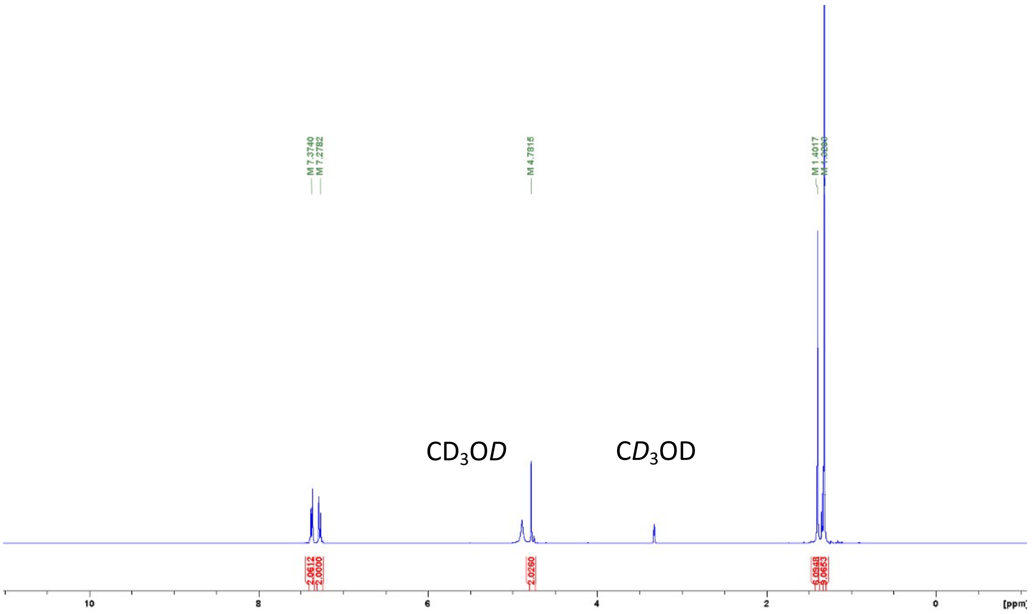

^{13}C NMR of **3i** in CD_3OD:

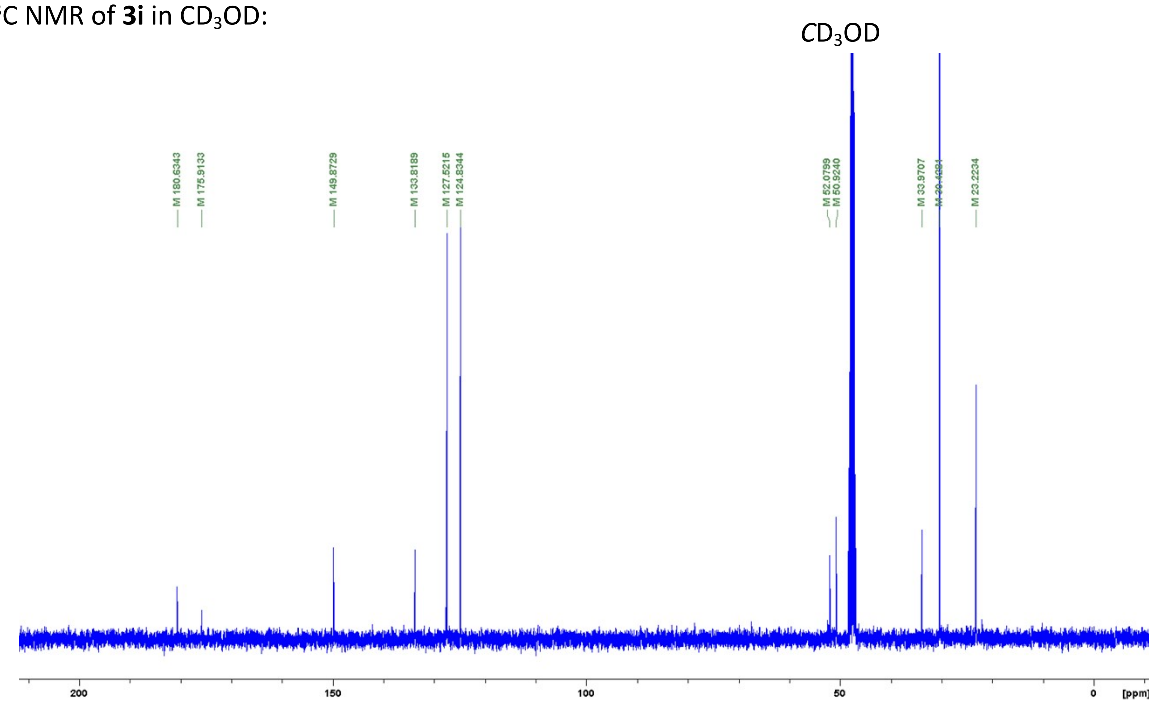

References

1 A. H. Y. Chan, I. Fathoni, T. Ho, K. J. Saliba and F. J. Leeper, *RSC Med. Chem.*, 2022, **13**, 817-821.

2 R. J. W. Allen and K. Kirk, *Mol. Biochem. Parasitol.*, 2010, **169**, 63–65.

3 E. T. Tjhin, C. Spry, A. L. Sewell, A. Hoegl, L. Barnard, A. E. Sexton, G. Siddiqui, V. M. Howieson, A. G. Maier, D. J. Creek, E. Strauss, R. Marquez, K. Auclair and K. J. Saliba, *PLoS Pathog.*, 2018, **14**, e1006918.

4 J. D. Johnson, R. A. Dennull, L. Gerena, M. Lopez-Sanchez, N. E. Roncal and N. C. Waters, *Antimicrob. Agents Chemother.*, 2007, **51**, 1926–1933.

5 A. H. Y. Chan, T. C. S. Ho, D. R. Parle and F. J. Leeper, *Org. Biomol. Chem.*, 2023, **21**, 1755-1763.

6 A. H. Y. Chan, T. C. S. Ho, K. Agyei-Owusu and F. J. Leeper, *Org. Biomol. Chem.*, 2022, **20**, 8855–8858.

7 B. Jahn, N. S. W. Jonasson, H. Hu, H. Singer, A. Pol, N. M. Good, H. J. M. O. den Camp, N. C. Martinez-Gomez and L. J. Daumann, *J. Biol. Inorg. Chem*, 2020, **25**, 199–212.

8 D. A. Walsh, R. H. Cooper, R. M. Denton, B. J. Bridges and P. J. Randle, *Biochemistry*, 1976, **157**, 41–67.

9 S. Mann, C. Perez Melero, D. Hawksley and F. J. Leeper, *Org. Biomol. Chem.*, 2004, **2**, 1732.

10 D. Zhu, S. Johannsen, T. Masini, C. Simonin, J. Haupenthal, B. Illarionov, A. Andreas, M. Awale, R. M. Gierse, T. van der Laan, R. van der Vlag, R. Nasti, M. Poizat, E. Buhler, N. Reiling, R. Müller, M. Fischer, J.-L. Reymond and A. K. H. Hirsch, *Chem. Sci.*, 2022, **13**, 10686–10698.

11 R. Hamid, S. Adam, A. Lacour, L. M. Gomez and A. K. H. Hirsch, BioRxiv, 2022, doi: 10.1101/2022.07.04.498669. This content is a preprint and has not been peer-reviewed.

12 A. H. Y. Chan, T. C. S. Ho, R. Irfan, R. A. A. Hamid, E. S. Rudge, A. Iqbal, A. Turner, A. K. H. Hirsch and F. J. Leeper, *Bioorg. Chem.*, 2023, **138**, 106602.

13 Y. Matsue, H. Mizuno, T. Tomita, T. Asami, M. Nishiyama and T. Kuzuyama, *J. Antibiot.*, 2010, **63**, 583–588.

14 V. M. Howieson, E. Tran, A. Hoegl, H. L. Fam, J. Fu, K. Sivonen, X. X. Li, K. Auclair and K. J. Saliba, *Antimicrob. Agents Chemother.*, 2016, **60**, 7146–7152.

15 F. Wohnsland and B. Faller, *J. Med. Chem.*, 2001, **44**, 923–930.

16 C. Zhu, L. Jiang, T.-M. Chen and K.-K. Hwang, *Eur. J. Med. Chem.*, 2002, **37**, 399–407.

17. D. Merk, F. Grisoni, L. Friedrich, E. Gelzinyte and G. Schneider, J. Med. Chem., 2018, 61, 5442–5447.

18 D. Hayashi, N. Kato, T. Kuzuyama, Y. Sato and J. Ohkanda, *Chem. Commun.*, 2013, **49**, 5535.

19 J. H. Chang, United States patent US4405357A, 1983.

20 X. Cao, B. He, F. Liu, Y. Zhang, L. Xing, N. Zhang, Y. Zhou, C. Gong and W. Xue, *RSC Adv.*, 2023, **13**, 6459–6465.

21 F. Cheng, D. Li, J. Li, Y. Tang, Y. Wu and S. Xu, *Org. Lett.*, 2023, **25**, 2555–2559.

22 M. J. Konz, United States patent US4302238, 1981.
